# The Essential Complexity of Auditory Receptive Fields

**DOI:** 10.1371/journal.pcbi.1004628

**Published:** 2015-12-18

**Authors:** Ivar L. Thorson, Jean Liénard, Stephen V. David

**Affiliations:** 1 Oregon Hearing Research Center, Oregon Health & Science University, Portland, Oregon, United States of America; 2 Department of Mathematics, Washington State University, Vancouver, Washington, United States of America; University of California at Berkeley, UNITED STATES

## Abstract

Encoding properties of sensory neurons are commonly modeled using linear finite impulse response (FIR) filters. For the auditory system, the FIR filter is instantiated in the spectro-temporal receptive field (STRF), often in the framework of the generalized linear model. Despite widespread use of the FIR STRF, numerous formulations for linear filters are possible that require many fewer parameters, potentially permitting more efficient and accurate model estimates. To explore these alternative STRF architectures, we recorded single-unit neural activity from auditory cortex of awake ferrets during presentation of natural sound stimuli. We compared performance of > 1000 linear STRF architectures, evaluating their ability to predict neural responses to a novel natural stimulus. Many were able to outperform the FIR filter. Two basic constraints on the architecture lead to the improved performance: (1) factorization of the STRF matrix into a small number of spectral and temporal filters and (2) low-dimensional parameterization of the factorized filters. The best parameterized model was able to outperform the full FIR filter in both primary and secondary auditory cortex, despite requiring fewer than 30 parameters, about 10% of the number required by the FIR filter. After accounting for noise from finite data sampling, these STRFs were able to explain an average of 40% of A1 response variance. The simpler models permitted more straightforward interpretation of sensory tuning properties. They also showed greater benefit from incorporating nonlinear terms, such as short term plasticity, that provide theoretical advances over the linear model. Architectures that minimize parameter count while maintaining maximum predictive power provide insight into the essential degrees of freedom governing auditory cortical function. They also maximize statistical power available for characterizing additional nonlinear properties that limit current auditory models.

## Author Summary

Understanding how the brain solves sensory problems can provide useful insight for the development of automated systems such as speech recognizers and image classifiers. Recent developments in nonlinear regression and machine learning have produced powerful algorithms for characterizing the input-output relationship of complex systems. However, the complexity of sensory neural systems, combined with practical limitations on experimental data, make it difficult to apply arbitrarily complex analyses to neural data. In this study we pushed analysis in the opposite direction, toward simpler models. We asked how simple a model can be while still capturing the essential sensory properties of neurons in auditory cortex. We found that substantially simpler formulations of the widely-used spectro-temporal receptive field are able to perform as well as the best current models. These simpler formulations define new basis sets that can be incorporated into state-of-the-art machine learning algorithms for a more exhaustive exploration of sensory processing.

## Introduction

Encoding models provide a powerful, objective means to evaluate our understanding of how sensory neural systems represent complex natural stimuli [1, 2]. An encoding model describes any time-varying neural signal (single- or multiunit activity [3, 4], local field potential [5], hemodynamic activity [6], or behavior [7]) as a function of the input stimulus, and it can predict the neural response to an arbitrary novel stimulus, including complex natural sounds. Prediction accuracy provides a quantitative measure of how well a model describes sensory-evoked activity; a completely accurate model should predict neural responses to any stimulus without error. More accurate models of sensory neural activity provide insight into algorithms that can be integrated into automated systems, such as speech recognizers and image classifiers.

In the auditory system, the linear spectro-temporal receptive field (STRF), implemented as a finite impulse response (FIR) filter, is the established “standard model” for neural representation [2, 4, 8–13]. This filter forms the core of generalized linear models (GLMs) applied to the auditory system [14, 15], and models sharing the same analytical form as the FIR STRF have been developed for studying visual [16–18], somatosensory [19, 20], and olfactory systems [21]. Despite its widespread use, careful assessments of how well the linear STRF actually describes auditory neural activity are limited [22]. A few studies have shown that the linear STRF can explain only a limited portion of sound-evoked activity in cortex, especially for complex natural stimuli [9, 23]. Others have argued that nonlinear variants of the classical linear STRF can improve predictive power [3, 24–33]. These nonlinear variants of the STRF show improved predictive power under specific experimental conditions. However, the more complex models are difficult to estimate reliably when experimental data are limited [1, 18, 22], especially for natural stimuli [12, 23, 34]. Difficulties associated with fitting and testing have prevented any single alternative from replacing the linear STRF as a new standard.

The challenges encountered when evaluating alternatives to the FIR STRF highlight the trade-off between model *performance*, how accurately it predicts neural activity, and *complexity*, the degrees of freedom governing the stimulus-response relationship [35, 36]. In order to completely describe a system’s function, an encoding model must account for all the degrees of freedom of the actual system. If the system is not well understood, some degrees of freedom in a model are likely to be mismatched to the system’s function. Any mismatched complexity does not provide additional explanatory power, but it does introduce noise into model parameter estimates. Because this complexity does not improve performance, there should exist a model with fewer degrees of freedom that can perform as well as the more complex model.

In this study we focus on the problem of complexity. Rather than simply seeking the model that performs best, we identify the simplest possible model that attains a minimum level of performance. Specifically, we ask, can we produce a low-dimensional approximation of the linear STRF that performs as well as the full FIR STRF? The idea of improving STRF performance by dimensionality reduction has been proposed previously. Isolated studies have shown benefits of low-rank approximations of the STRF [28, 31, 37, 38]. In the visual system, several studies have also proposed low-dimensional, system-specific parameterizations [18, 29, 39–43]. Despite the many parameterizations that have been proposed, however, direct comparisons between them have been limited, especially for natural stimuli. Thus it remains difficult to identify the important features of these different models.

We approached the complexity problem directly by systematic comparison of a large set of alternative parameterizations. We generated a collection of models that instantiate a variety of low-dimensional approximations to the FIR STRF. We then compared their performance on single-unit data collected from primary auditory cortex during presentation of natural vocalizations. By exploring the performance of this family of models, we were able to identify the minimal essential components required by linear STRFs that best described the data and to study the relationship between the amount of data available and optimal model complexity.

We found that the standard FIR STRF is suboptimal according to the complexity criterion. Instead, a much simpler model, which defines the STRF as a product of three Gaussian-tuned spectral filters and biphasic temporal filters, outperformed the FIR STRF, while requiring only about 10% of the parameters (29 vs. 276 free parameters). These results indicate that, for the average A1 neuron, a model with about 30 free parameters is able to capture its linear filter properties. The total degrees of freedom of a comprehensive nonlinear model is likely to be higher, but our minimally complex linear STRF provides a starting point for developing better-performing nonlinear models.

## Results

### Predictive model framework

**Fig 1:**
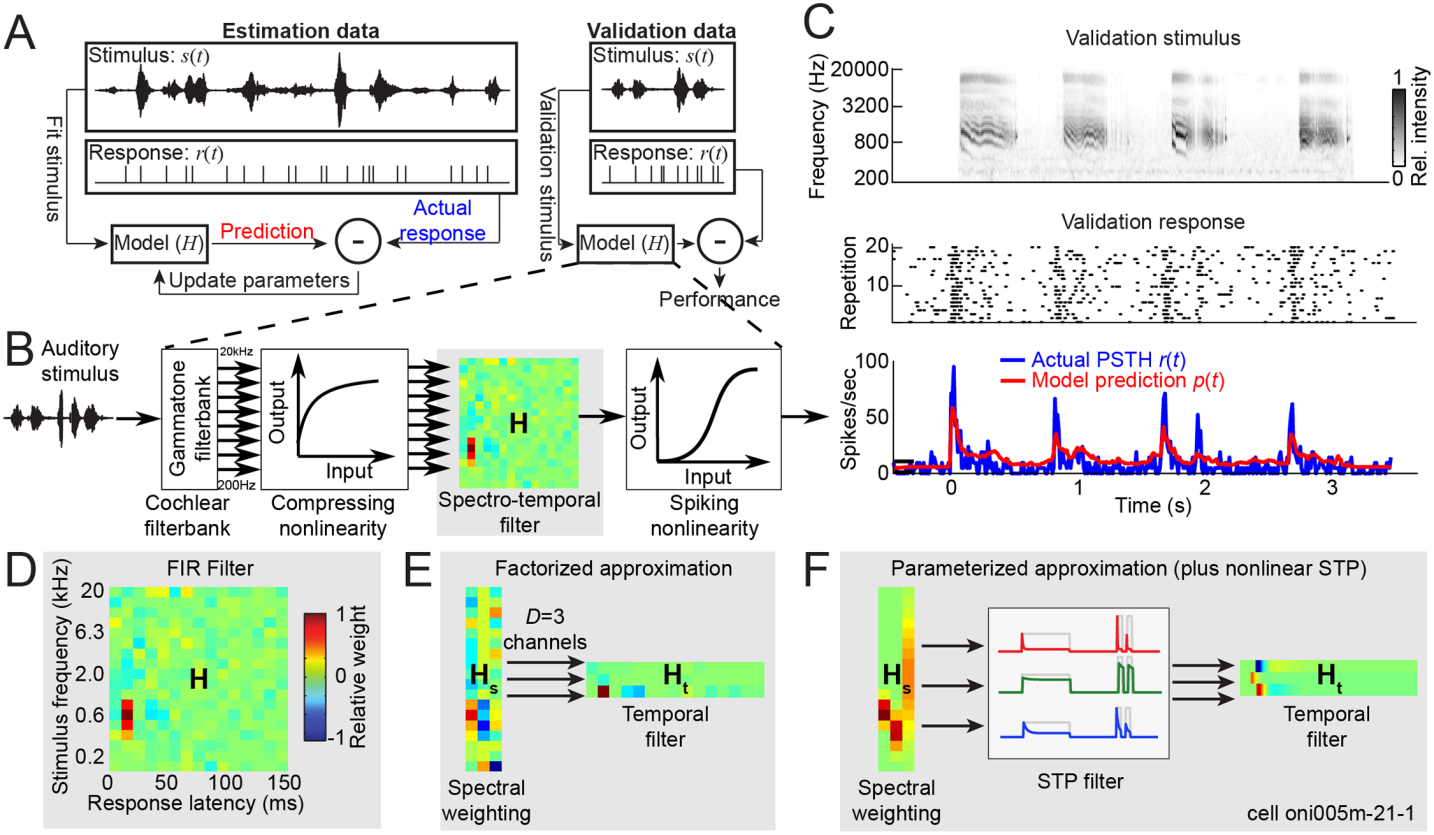
Model estimation and validation procedures. **A.** Data for each neuron were split into an estimation data set, used to fit model parameters, and a validation data set, used only for testing prediction accuracy. **B.** Models were defined by a sequence of functions mapping stimulus to predicted response. This study focused on alternative parameterizations of the spectro-temporal filter module, and other modules were kept fixed for most comparisons. **C.** Spectrogram of example 3-second ferret vocalization used for testing prediction accuracy (top). Raster response of one neuron to 20 repetitions (middle) and PSTH binned at 100 Hz (bottom, blue). Predicted PSTH response is overlaid in red. **D.** The STRF is typically implemented as a multichannel finite impulse response (FIR) filter, requiring one parameter for each frequency and time lag. In this heat map, red areas indicate stimulus frequencies and time lags associated with increased neuronal spike rate and blue areas with decreased rate. **E.** Factorized STRF approximation is generated by the outer product of spectral- and temporal filter matrices, reducing the total parameter count. **F.** Parameterized models generate factorized matrices from parametric tuning curves, further reducing parameter count. Example Gaussian spectral and P3Z1 temporal parameterizations are shown. Factorized and parameterized models also permit the insertion of nonlinear modules, such as a filter mimicking short-term plasticity (STP), after projection onto the spectral channels.

We recorded single-unit neural activity from the auditory cortex (A1) of awake, passively listening ferrets during presentation of natural ferret vocalizations. The same set of 42 3-second vocalizations was presented during recordings from all neurons (*N* = 176). We then fit a large number of encoding models with different architectures to data from each neuron and compared their performance. Data for each neuron were grouped into an estimation data set (40 vocalizations), which was used for fitting, and a validation data set (2 vocalizations), which was used only to test how well each fit predicted responses to a novel stimulus (Fig 1A). Our primary performance metric was prediction correlation, *i.e.*, the correlation coefficient (Pearson’s *R*) between the actual peri-stimulus time histogram (PSTH), *r*(*t*), and the PSTH predicted by the model, *p*(*t*) (Fig 1C). Other commonly used performance metrics showed the same pattern of results (*e.g.*, log-likelihood and mutual information, see below).

Models were structured as a sequence of signal transformations, or functional *modules*, corresponding to the block diagram in Fig 1B,

$$\begin{array}{c} x_{0}\left(t\right)\overset{f_{1}\left(\cdot \right)}{\to }x_{1}\left(t\right)\overset{f_{2}\left(\cdot \right)}{\to }\cdots \overset{f_{n}\left(\cdot \right)}{\to }y\left(t\right) \end{array}$$ (1)

where the output, *x*_*i*_(*t*), of each module, *f*_*i*_(⋅), provides the input into the subsequent module. The final module produced the predicted time-varying spike rate, *y*(*t*). In most models tested, this sequence consisted of three modules, a cochlear filterbank [26, 44], followed by a linear spectro-temporal filter [8, 9, 11, 12], and finally an output nonlinearity to account for spike generation thresholds [13, 17].

**Fig 2:**
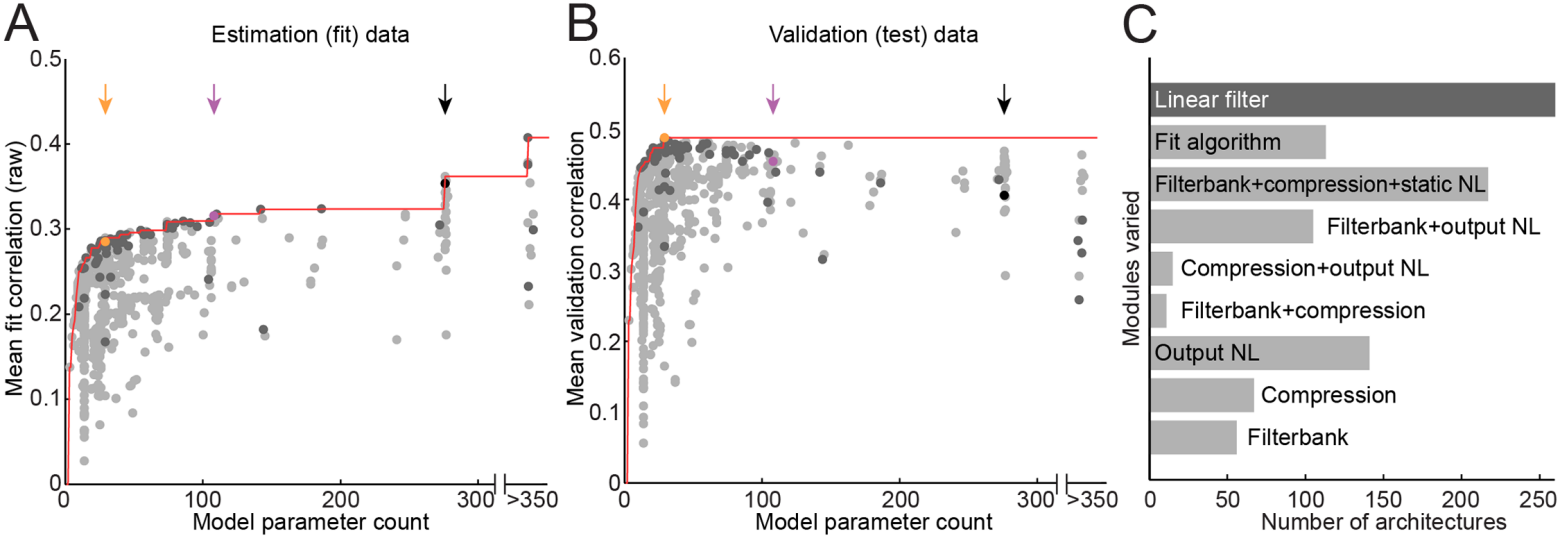
Model complexity vs. performance. Pareto plots compare model parameter count (horizontal axis) versus prediction correlation (vertical axis) for each linear STRF architecture, averaged over *N* = 176 A1 neurons. The Pareto front (red line) indicates the best prediction correlation for models with parameter count at or below the current abscissa. Dark gray points indicate models at the focus of this study, varying in only in the linear filter module and sharing the same input compression, spike nonlinearity, and fit algorithm. Black, purple, and orange points/arrows indicate the FIR, factorized, and parameterized models, respectively, explored in detail in later sections. **A.** For estimation data, prediction correlation tends to increase for more complex models. **B.** For validation data, performance reaches its maximum at just 29 parameters, suggesting that the increase in the estimation data for higher parameter counts reflects overfitting to noise by the more complex models. **C.** Summary of model architecture variants. Each bar shows the number of architectures evaluated using modules and fitting algorithms that differed from the core set of modules and procedures detailed in subsequent figures. For example, “Filterbank+output NL” indicates the number of models tested with a filterbank other than the second-order gammatone (Eq 11) and output nonlinearity other than the double exponential sigmoid (Eq 14).

Alternative model architectures were compared by replacing one or more modules in Eq 1, while keeping the others the same. Thus the impact of the choice for each module on model performance could be tested individually (see Fig 2C). Using this empirical approach, we selected optimal modules for the cochlear filterbank (Eqs 11–13) and output nonlinearity (Eq 14) for the same linear filter module (FIR filter, see below, Eq 3). These modules were then held constant while we compared performance for the different formulations of the linear filter module that follow.

Models were fit using an iterated coordinate descent (a.k.a. boosting) algorithm [34]. On each iteration, the algorithm cycled through each module sequentially and performed a few steps of coordinate descent within that module before moving on to the next one (see Methods). We have previously demonstrated that this coordinate descent algorithm is able to accurately recover linear STRFs in simulation [30, 34].

Because datasets are finite, the performance of any model will be limited by sampling noise. This noise impacts the analysis at two stages: producing error in the estimation of model parameters and in validation of prediction accuracy [18, 22, 45]. Accounting for the first problem is a nuanced issue: more complex models that require a large number of parameters are more susceptible to noise than simpler models. We address the issue of finite estimation data in a later section (see Parameterized models perform similarly to FIR models in the limit of infinite data, below). To account for the latter problem, measures of prediction correlation were normalized by a factor reflecting response reliability in the validation stimulus (Eq 23, [45]). This factor was fixed for an individual neuron’s validation data. Thus it does not affect the performance of one model relative to another. Numerically, this correction increased prediction correlations in A1 by a mean of 20% (ranging from 3% to 39% for individual neurons).

### Pareto front describes a trade-off between model performance and complexity

Model complexity is often factored into cost functions for model fitting, in order to positively weigh simpler models [35, 46]. Our goal was to study in depth the relationship between model complexity and performance. Thus, rather than combining them into a single cost function, we studied the trade-off between these criteria in detail, exploring the family of solutions that are optimal with respect to both. This optimal set of solutions is known as the Pareto front [36, 47]. Formally, all items belonging to this front are non-dominated in the Pareto sense [47] which means that for all pairs of models on the front, one is less complex while the other fits more closely to the data. All models below the Pareto front are non-optimal: there is at least one model on the front that is both less complex and more accurate.

We generated Pareto plots for the 1061 different linear STRF architectures tested, comparing model parameter count against average prediction correlation for estimation data (Fig 2A) and validation data (Fig 2B). Most models lie under the Pareto front (red line) and are suboptimal relative to models that are less complex, better performing, or both. More complex models tend to perform better for estimation data, but they do not necessarily predict novel validation data more accurately. The differences between estimation and validation plots illustrate the problem of overfitting when available estimation data are finite. Among the more complex models, the FIR STRF falls below the Pareto front for the validation data (black point, Fig 2B). Instead, best performance in the current dataset is achieved by a model requiring just 29 parameters (orange point).

In the following sections, we discuss in detail the subset of 260 architectures in which only the linear filtering module was varied, while all other modules (cochlear filterbank, input nonlinearity, output nonlinearity) and the fitting algorithm were held constant (dark gray points, Fig 2A and 2B). Our focus is on identifying model architectures that fall on or near the Pareto front, making them optimal for a given level of complexity. The remaining models were generated by manipulating one or more modules other than the linear filter (Fig 2C). Varying the other modules had less dramatic effects on model complexity and performance, but they provide a dense sampling of the complexity-performance space. A complete list of architectures evaluated is included in the supplementary materials (S1 Table).

### STRF parameterization improves model predictive power

#### Finite impulse response (FIR) STRF

Classically, the STRF has been implemented as a multi-channel FIR filter (Fig 1D, [8]). At its core, the FIR STRF is simply a matrix of weights associated with different sound frequencies and time lags that predicts the time-varying neural firing rate by convolution with the stimulus spectrogram. The STRF has proven to be a useful tool for characterizing the feature selectivity of auditory neurons [4, 8, 10] and how that selectivity changes across the auditory hierarchy [48]. In addition, the FIR STRF has been used as a tool to study changes in sensory representation reflecting the modulatory effects of learning and attention [49, 50]. For a stimulus spectrogram, **x**(*t*) = [*x*_1_(*t*) *x*_2_(*t*) ⋯ *x*_*c*_(*t*)], with *C* channels binned each *τ* ms, the FIR filter, **H**, with a maximum memory of *U* time bins is a *C* × *U* matrix

$$\begin{array}{c} H=\left[\begin{array}{cccc} h_{11} & h_{12} & \cdots & h_{1U}\\ h_{21} & h_{22}\\ \vdots & & \ddots \\ h_{C1} & & & h_{CU} \end{array}\right] \end{array}$$ (2)

The time-varying output *y*_FIR_ of the filter is then the convolution with the stimulus in time and sum across frequencies,

$$\begin{array}{ccc} y_{\text{FIR}}\left(t\right) & = & b+\sum_{i=1}^{U}\sum_{f=1}^{C}h_{fi}x_{f}\left(t-\left(i-1\right)\tau \right) \end{array}$$ (3)

Positive values of coefficients *h*_*fi*_ indicate components of the stimulus that correlate with increased output, and negative values with decreased output. The constant term, *b*, accounts for the possibility of nonzero output even when the input is zero. Unless otherwise specified, in the following results, **x** is produced by passing the raw sound waveform through a cochlear filterbank with *C* = 18 channels, logarithmically spaced over 200–20,000 Hz [44]. The filterbank output is log-compressed (Eq 13) before input to the linear spectro-temporal filter. The duration of the filter is 150 ms (*i.e.,* *U* = 15 for *τ* = 10 ms temporal bins), requiring *C* × *U* = 270 parameters. From the linear filter, the signal finally passes through a static output nonlinearity that accounts for spike threshold and saturation (Eq 14). An additional 6 parameters for the baseline response, input compression and output nonlinearity make a total of 276 for the entire FIR STRF. We explored the impact of varying *C* (see below) and *U* (S1 Table), and found no improvement for smaller or larger values of either parameter. Moreover, changing spectral or temporal resolution had no impact on the relative performance of the different model architectures compared below.

**Fig 3:**
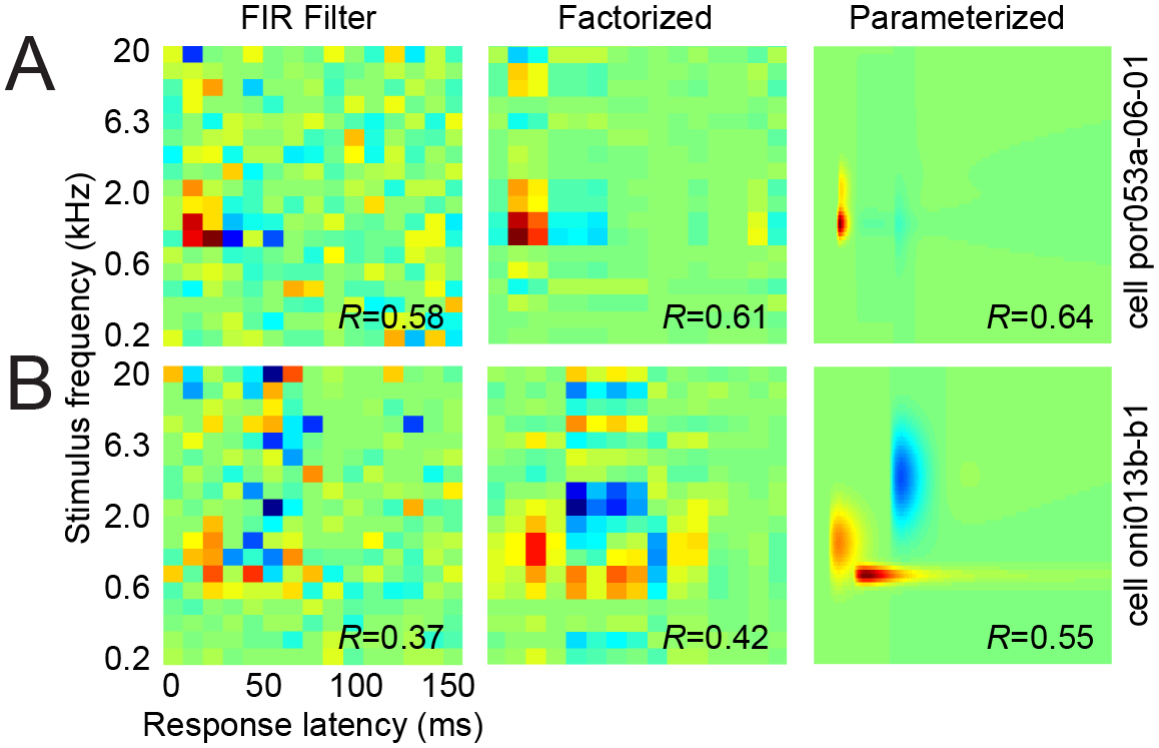
Example STRF fits for different model architectures. **A.** STRF weight matrices (*H*) for the FIR, factorized, and parameterized models fit to the same neuron. Prediction correlation (Pearson’s *R*) is indicated in the corner of each STRF. For factorized and parameterized models, the STRF is computed from the outer product of the spectral and temporal filters that specify those models, **H** = **H**_*s*_ **H**_*t*_. Dimensionality (*D*) of the spectral and temporal filters constrains frequency-time separability of the STRF, but it does not restrict tuning bandwidth (*i.e.*, spectral tuning can span more than *D* channels, as in this example). The factorized and parameterized models exhibit less spurious noise, are easier to interpret, and show improved prediction accuracy over the full FIR. **B.** Example STRFs for a second neuron exhibit more complex tuning and basis functions in the parameterized model can account for tuning to distinct spectro-temporal features.

Fits using the full FIR implementation of the STRF typically show localized spectro-temporal regions of large excitatory and/or inhibitory filter weights, indicating a best frequency (BF) and response latency for each neuron (Fig 3, left panels). Although we did not explicitly compare tuning for different stimuli in the current study, the BF measured from the STRF is typically similar to the BF measured from other stimuli, such as pure tones [5] or broadband noise [9]. Additional features of the STRF, away from BF and peak latency, often do depend on the stimulus used for STRF estimation, and thus it is more challenging to determine if these features reflect off-peak tuning or estimation noise.

#### Factorization of the FIR matrix

The first strategy we explored for reducing the number of parameters required for the linear STRF was a factorized model (Fig 1E, [37, 38]). This model follows the same sequence of modules as the FIR STRF, but the linear filter, **H**, is approximated as the product of a *C* × *D* spectral weighting matrix, **H**_*s*_, and a *D* × *U* temporal filtering matrix, **H**_*t*_,

$$\begin{array}{c} H=H_{s}H_{t} \end{array}$$ (4)

A model with dimensionality *D* = 1 is often referred to as a space-time separable model, a common strategy for dimensionality reduction [20, 28, 51]. Varying *D* impacts the complexity of the spectro-temporal filter [37, 38], and we explored the effect of different values of *D* on model performance. Factorization is closely related to reduced-rank approximations of the STRF, except that the factorized dimensions are not required to be orthogonal. For the linear model, this distinction is trivial and has no effect on theoretical performance. However, when nonlinear terms are introduced between the spectral and temporal filtering stages, non-orthogonal spectral dimensions allow for additional model functionality (see STP STRF, below).

The factorized **H** can be interpreted as breaking down the linear filter module into a sequence of two modules (Eq 1, Fig 1E). First, a set of spectral filters, **h**_*s*_*j*__ (rows of **H**_*s*_), maps the *C*-dimensional input spectrogram into a *D*-dimensional subspace, **s**(*t*) = [*s*_1_(*t*) *s*_2_(*t*) ⋯ *s*_D_(*t*)],

$$\begin{array}{c} s_{j}\left(t\right)=\sum_{f=1}^{C}h_{s_{j}}\left(f\right)x_{f}\left(t\right) \end{array}$$ (5)

Second, the signal in each dimension of this new spectral subspace is convolved with a temporal filter, ****h**_*t*_*j*__** (columns of **H**_*t*_), before summing across spectral channels,

$$\begin{array}{c} y_{\text{FAC}}\left(t\right)=b+\sum_{i=1}^{U}\sum_{j=1}^{D}h_{t_{j}}\left(i\right)s_{j}\left(t-\left(i-1\right)\tau \right) \end{array}$$ (6)

Factorization reduces the number of parameters required to define **H** to *D* × (*C* + *U*), which is usually much less than *C* × *U*. Although small values of *D* constrain the spectro-temporal complexity of the STRF, they do not limit spectral tuning bandwidth, as a single spectral channel can have arbitrarily broad tuning bandwidth. Similarly, the factorized temporal filter can integrate across many time lags.

**Fig 4:**
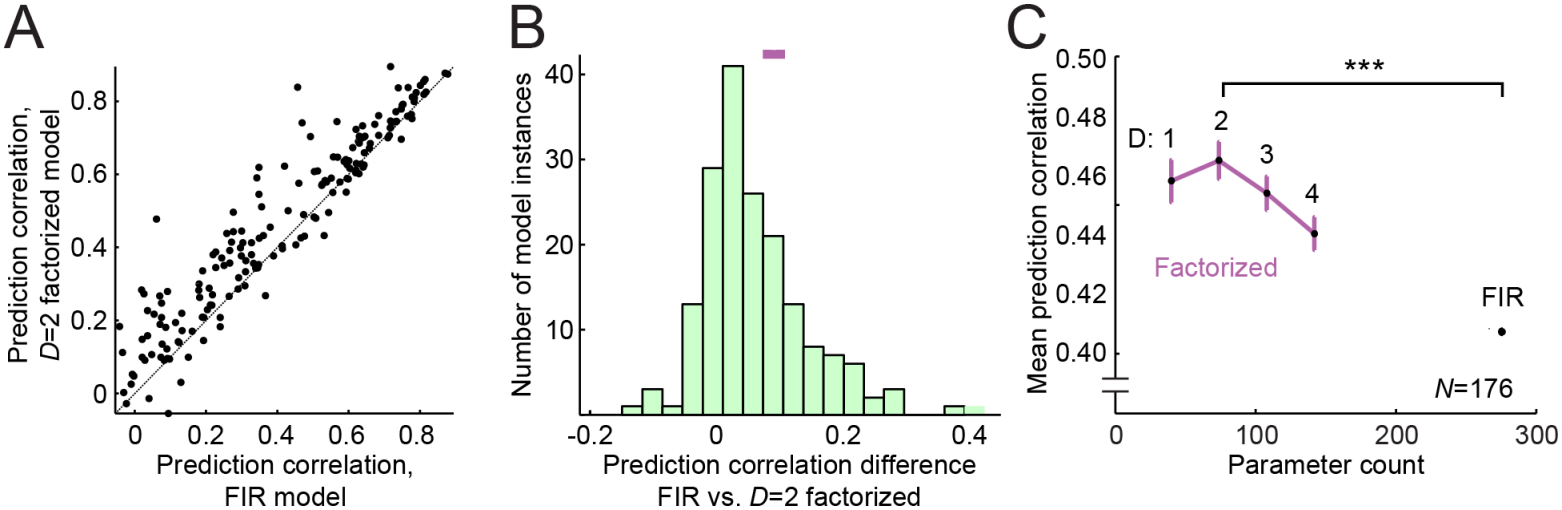
Factorized model performance. **A.** Scatter plot of *D* = 2 factorized- versus FIR model prediction correlation per neuron shows that the factorized model predicts more accurately for most neurons. **B.** Histogram of difference in prediction correlation *D* = 2 factorized and FIR models for each neuron. Error bar at top shows 1 SEM on the difference between model predictions, illustrating the procedure for measuring error bars in the comparisons of average model performance that follow (see Methods). **C.** Pareto plot compares model complexity (parameter count) versus mean prediction correlation for the FIR model and factorized models, plotted as in Fig 2 (channel count, *D* = 1…4). Error bars calculated as in B, relative to the FIR STRF. Despite having fewer parameters, the factorized models perform consistently better than the FIR (*p* < 0.001, sign test, *N* = 176). The *D* = 2 factorized model performs best, with about a 15% average increase in correlation over the FIR STRF, despite requiring about one-quarter of the parameters.

We compared performance of the factorized model for different values of *D*. The same fitting algorithm was used as for the FIR STRF, but iterating separately on the spectral- and temporal filter modules (see Methods). Across the entire vocalization dataset, factorization with *D* = 2 spectral channels produced the highest mean prediction correlation and performed significantly better than the FIR STRF (Fig 4A and 4B, mean *R* = 0.464 vs. 0.406, *p* < 0.0001, sign test). Depending on the convergence of synaptic inputs, there is a theoretically optimal number of dimensions *D* that describe spectro-temporal selectivity [37]. Beyond that number, STRF performance should asymptote to the performance of the full FIR STRF. In practice, however, factorized models for all values of *D* tested surpassed the FIR STRF performance, indicating that the reduced number of parameters also improves model performance by reducing estimation noise (Fig 4C).

#### Factorized models motivate parameterization for further dimensionality reduction

To explore models with further reduced complexity, we considered ways to approximate spectral and temporal tuning with even fewer parameters than the factorized model. In the factorized model, the columns of **H**_*s*_ and the rows of **H**_*t*_ describe selectivity in the spectral and temporal domains, respectively. These spectral and temporal tuning functions are nonparametric, in the sense that they are not constrained to have a specific functional form. We identified candidate parameterizations of these curves by measuring their average shape across the neural population for the *D* = 2 factorized model.

**Fig 5:**
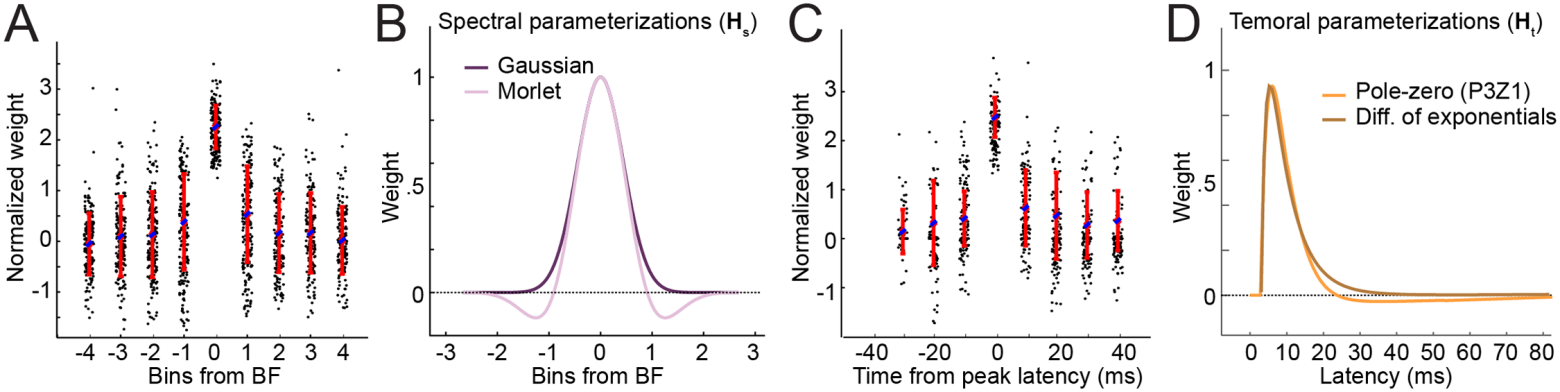
Parameterization of spectral and temporal tuning. **A.** Values of factorized spectral weighting matrix **H**_*s*_, centered about their peak and normalized by variance across all weights. **B.** Based on the Gaussian-like mean weights and presence of negative values in the sidebands, we parameterized spectral filters using either a Gaussian function or Morlet wavelet. **C.** Values of the temporal weighting matrix **H**_*t*_ binned at 100 Hz and aligned at peak latency. The -40 ms bin was cropped because very few neurons had peak latency longer than 40 ms. The asymmetric rapid onset and slower fall-off are not well-described by a Gaussian. **D.** Temporal filters were parameterized using either a difference of exponentials or a pole-zero filter.

When spectral filters were aligned at best frequency (BF, Fig 5A), sensitivity in neighboring frequency bands covaried with the response at BF, producing approximately Gaussian tuning. Weights for off-BF bins were often negative, suggesting that sideband inhibition could be useful to include in a parameterized model. After subtracting the mean, we also performed principal components analysis to identify any additional patterns in the distribution of **H**_*s*_ fits, but the resulting principal components were quite small (29%, 13% of the variance) and did not have any clear structure. Based on the average tuning curve, we hypothesized that **H**_*s*_ might be well parameterized by one of two functions (Fig 5B): a Gaussian function, parameterized by a mean frequency and tuning bandwidth, or a Morlet wavelet, which also permits inhibitory sidebands.

Temporal filters were aligned at their peak latency before averaging (Fig 5C). They tended to have a longer tail following the peak latency, compared to a relatively rapid rise before the peak. Thus they were not well characterized by a Gaussian. The first two principal components of **H**_*t*_ were again small (31%, 14% of variance) but resembled high-pass filters (*i.e.*, temporal differentiators), consistent with sensitivity to changes in stimulus intensity within a spectral frequency band. We therefore hypothesized that **H**_*t*_ might be well-parameterized by either a difference of exponentials, describing a fast rise followed by a slow decay [29], or a more general linear filter that could generate peaked impulse responses yet could also be weakly high pass (Fig 5D).

#### Gaussian spectral parameterization

We parameterized the spectral channel matrix **H**_*s*_ using a single Gaussian function per channel (Fig 5B). Thus the weights for column, *j*, and frequency bin, *i*, are specified by two parameters, center frequency *f*_0_ and bandwidth *σ*,

$$\begin{array}{c} h_{s_{j}}\left(i\right)=\frac{1}{\sigma \sqrt{2\pi }}\exp\left(-\frac{{\left(f_{i}-f_{0}\right)}^{2}}{2\sigma^{2}}\right) \end{array}$$ (7)

**Fig 6:**
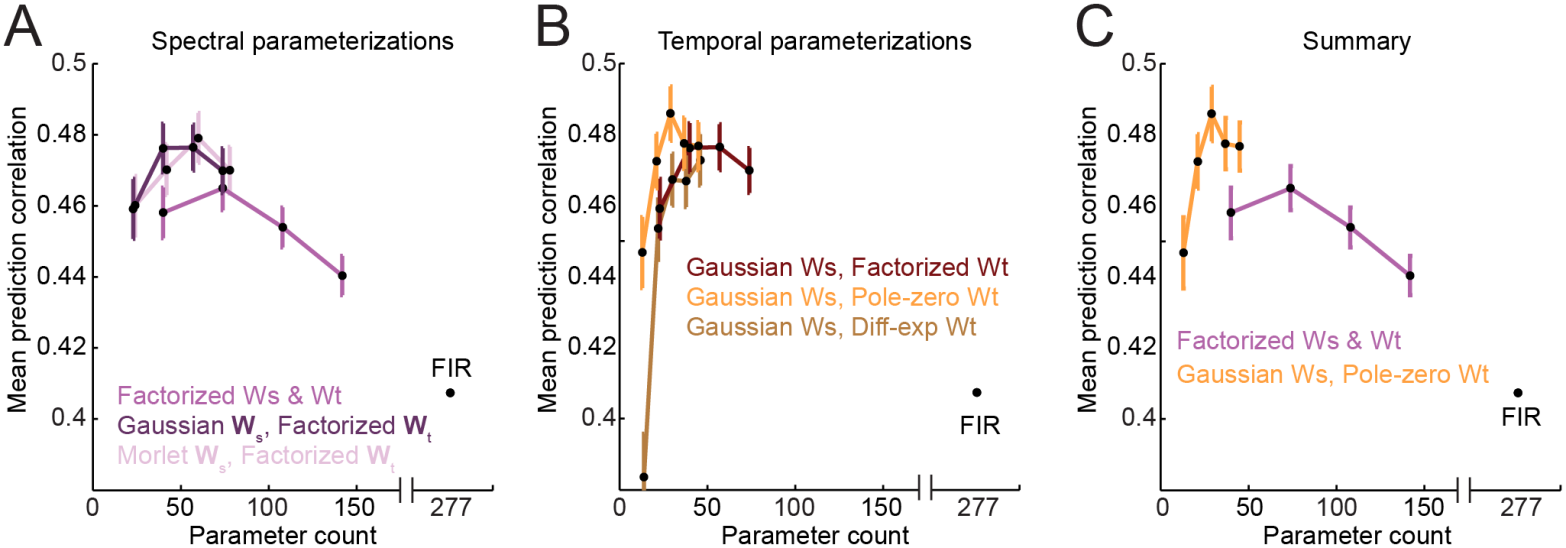
Parameterized model performance. **A.** Average performance of spectrally parameterized (Gaussian and Morlet) models (*W*_*s*_), using a factorized temporal filter (*W*_*t*_) and plotted as in Fig 4C. For a given spectral channel count, *D*, the improvement over the factorized model is significant for both parameterizations (*p* < 0.01, sign test), despite requiring fewer parameters. **B.** Average performance of temporally parameterized models, using Gaussian spectral parameterization and plotted as in A. The P3Z1 pole-zero parameterization performed significantly better than the difference of exponentials (*p* < 0.01, sign test), although neither showed a significant difference from the factorized temporal filter. **C.** Summary of performance for the factorized model and the best parameterized model (Gaussian *W*_*s*_, P3Z1 *W*_*t*_) for each channel count. Performance of the parameterized model is significantly better than the factorized (*p* < 0.01, sign test) and the FIR model (*p* < 0.001).

For *D* spectral channels, the Gaussian spectral filter requires only 2*D* parameters, compared to *C* × *D* parameters for the factorized model. When we replaced the factorized spectral filter with the Gaussian parameterization (while preserving the factorized temporal filter), model performance again improved, despite the further decrease in parameter count (Fig 6A). The *D* = 3 Gaussian spectral model showed the highest average correlation (mean *R* = 0.476), significantly surpassing the performance of the FIR STRF by about 10% (*p* < 0.0001) and the best factorized model by about 3% (*p* < 0.01, sign test).

#### Morlet spectral parameterization

The Morlet wavelet provided an alternative spectral parameterization similar to the Gaussian (Fig 5B), which could also account for sideband inhibition. The Morlet wavelet determines coefficients according to three parameters, center frequency, *f*_0_, bandwidth, *σ*, and sideband amplitude, *z*,

$$\begin{array}{c} h_{s_{j}}\left(i\right)=ℜ\left(\exp\left(\frac{-{\left(f_{i}-f_{0}\right)}^{2}}{\sigma^{2}}-iz\left(f_{i}-f_{0}\right)\right)\right) \end{array}$$ (8)

where ℜ(⋅) indicates taking the real component. Thus 3*D* parameters are required for the *D* spectral filters. The best Morlet parameterization (*D* = 3) performed significantly better than the factorized model (Fig 6A, mean *R* = 0.479, *p* < 0.001, sign test). However, its performance was not significantly different from the Gaussian parameterization. Because increasing the parameter count to account for spectral sidebands did not improve predictive power, we subsequently focused on the Gaussian spectral parameterization.

#### Difference of exponentials temporal parameterization

The difference of exponentials temporal filter produces a family of curves that resemble the mean of **H**_*t*_ (Fig 5D),

$$\begin{array}{c} h_{t_{j}}\left(i\right)=A_{1}\exp\left(-\frac{i-\theta_{1}}{\tau_{1}}\right)-A_{2}\exp\left(-\frac{i-\theta_{2}}{\tau_{2}}\right) \end{array}$$ (9)

where the output exp() is set to zero for *i* < *θ*_*n*_. This filter requires six parameters (*A*_1_, *τ*_1_, *θ*_1_, *A*_2_, *τ*_2_, *θ*_2_) per spectral channel and thus a total of 6*D* parameters, compared to *D* × *U* for the factorized temporal filter. The *D* = 3 difference of exponentials parameterization performed nearly as well as the Gaussian spectral model (mean *R* = 0.467, *p* > 0.05, sign test), indicating that this filter captures many of the features of the factorized temporal filter (Fig 6B).

#### Pole-zero temporal parameterization

**Fig 7:**
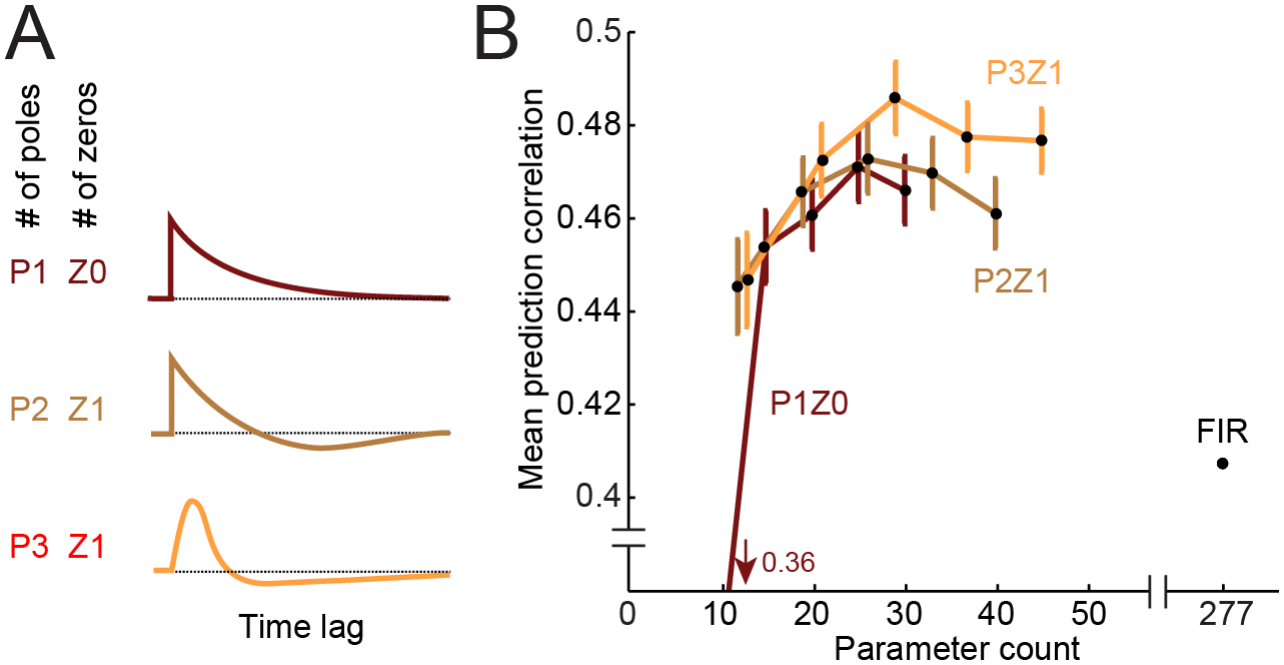
Pole-zero IIR filters. **A.** Examples of parameterized temporal filters with varying numbers of poles (*P*) and zeros (*Z*). Curve at top shows a very simple kernel with only one pole and no zeros (P1Z0). The curves below show more complex kernels requiring more parameters. **B.** Pareto plot comparing performance of the different pole-zero parameterizations, each for *D* = 1…5 spectral channels (Gaussian parameterization), plotted as in Fig 4C. The simplest temporal kernel requires more spectral channels to approach the performance of more complex kernels. The *D* = 3, P3Z1 kernel showed the trend for best performance overall.

Many physical systems are well-described by linear ordinary differential equations, or infinite impulse response (IIR) filters. This parameterization can be defined in the frequency domain using *pole-zero* (PZ) notation. We compared performance of parameterizations with variable numbers of poles and zeros (Fig 7A). The number of poles determines the shape of the filter, and the number of zeros determines the number of zero crossings. We also included parameters for filter gain and latency. Thus, a 3-pole, 1-zero filter (P3Z1) is defined in the frequency domain (*s*),

$$\begin{array}{c} G_{P3Z1}\left(s\right)=A\exp\left(-l\cdot s\right)\frac{s-z_{1}}{\left(s+p_{3}\right)\left(s+p_{2}\right)\left(s+p_{3}\right)} \end{array}$$ (10)

where *A* is magnitude, *l* is delay, *p*_1_, *p*_2_, *p*_3_ are the poles, and *z*_1_ is the zero (6 parameters per spectral channel). PZ filters provide a more general formulation of temporal dynamics than the difference of exponentials. In fact, it is possible to construct a *D* = 2, P1Z0 model that is exactly equivalent to a *D* = 1 difference of exponentials model. Here we constrained poles and zeros to be real, which restricted the impulse response to being a sum of decaying exponentials. Including complex poles and zeros doubles the number of parameters, and the neural data did not show the oscillatory responses that this would characterize.

We tested all possible combinations of 1 to 5 poles and zeros over *D* = 1 − 5 spectral channels to determine possible candidate kernels (Fig 7B). The best model used P3Z1 parameterization (*D* = 3, mean *R* = 0.485, Fig 6B). This model performed as well as the *D* = 3 Gaussian model (*p* > 0.05, sign test) and significantly better than the difference of exponentials parameterization (*p* < 0.01, sign test). The simplest one- or two-pole filters could not fully describe temporal encoding properties. Instead, a more complex temporal filter was required, and a combination of Gaussian spectral and P3Z1 temporal filter for *D* = 3 spectral channels gave the best performance among parameterizations of the linear STRF (Fig 6C).

### Parameterized models perform similarly to FIR models in the limit of infinite data

Parameterized STRFs are approximations of the FIR STRF. Thus, in theory, the FIR STRF should perform as well as or better than any parameterized STRF. In practice, however, data available for estimation are finite, and simpler models can be estimated more accurately than the full FIR STRF. Thus simpler models are able to perform better than the FIR STRF in our analysis (Fig 6). The results so far demonstrate a clear practical advantage of the factorized and parameterized models, but they do not answer the question of whether any simpler model fully accounts for the linear STRF. Such a question can only be answered by comparing the relative performance of these models in the limit of infinite estimation data [18, 22].

Extrapolating performance to infinite estimation data is challenging because there is no widely agreed upon model of variability in sensory-evoked neural activity. We made a simplifying assumption that prediction error from estimation noise is additive and inversely proportional to the square root of the number of samples used to estimate the STRF, *T* (see Methods, Eq 31, [18, 45]). When these assumptions hold, then the effect of noise on model variance explained (square of prediction correlation, *R* ^2^) also decreases proportionally to *T*. We varied *T* by subsampling the available estimation data (10%–75%) and measured the average *R*_*T*_ across neurons for models fit with the different data subsets. We then fit the free parameters in Eq 31 to determine the theoretical limit on performance for each model, *R*_inf_.

We measured the asymptotic performance limit of four model architectures, ranging from high to low complexity: the full FIR model (FIR, 276 parameters), *D* = 3 factorized model (Factorized x3, 109 parameters), *D* = 3 Gaussian spectral/P3Z1 temporal parameterization (P3Z1x3, 29 parameters), and *D* = 1 Gaussian spectral/P3Z1 temporal parameterization (P3Z1x1, 13 parameters). We removed very noisy data and focused on the subset of 124 neurons that produced reliable auditory-evoked responses (SNR > 0.005, see Methods, Eq 21).

**Fig 8:**
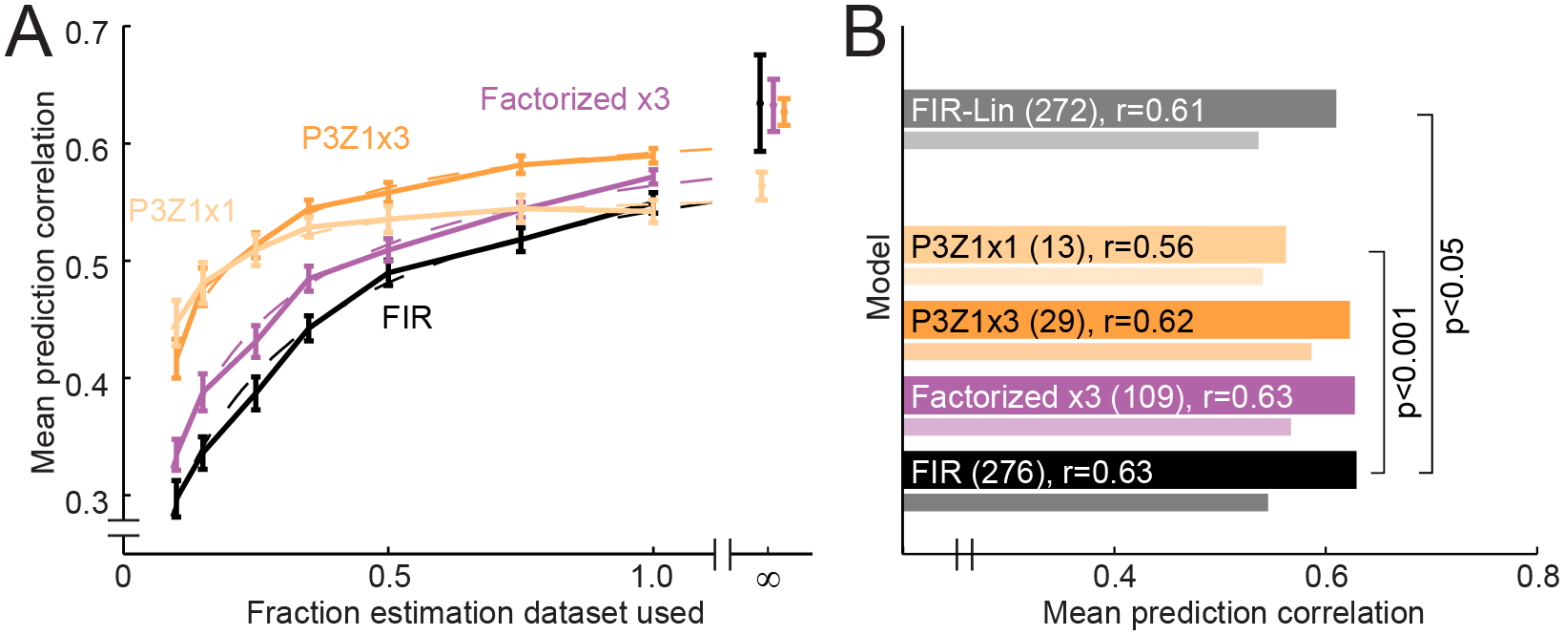
Theoretical performance in the limit of infinite estimation data. **A.** Curves compare average performance of models of varying complexity as a function of the fraction of estimation data used for fitting (*N* = 124 neurons with response SNR > 0.005). Error bars indicate the standard error on the mean difference between each model’s performance and average performance of all models estimated using 100% of available data. If only 10% of estimation data are used (12 sec of stimulation), the best-predicting model is the *D* = 1 P2Z1 model (peach), requiring only 13 parameters. For this estimation set, the FIR STRF (black, 276 parameters) performs substantially worse. As the quantity of available data increases, more complex models perform better, although the FIR model still lags behind the factorized (purple) and P3Z1 parameterized (orange) models of intermediate complexity. Fits to theses curves are plotted with dashed lines (Eq 31), and asymptotic performance is indicated at far right. Note that prediction correlation plotted for 100% of estimation data is higher than in Fig 6 because only high-SNR neurons are included here. **B.** Average asymptotic performance of each model in the limit of infinite estimation data. Actual performance for each model with 100% of estimation data is plotted with the thinner bars. The FIR STRF with no spike nonlinearity is also included (gray) for comparison with previous asymptotic analysis [22].

For all models, performance improved as more estimation data became available (Fig 8A). As expected, the lower-dimensional models performed better for small data sets and neared asymptotic performance sooner than higher-dimensional models. Consistent with this observation, performance of the FIR STRF showed the greatest improvement in the asymptote (*R*_inf_ = 0.63, Fig 8B). However, performance of the Factorized x3 (*R*_inf_ = 0.63) and P3Z1x3 models (*R*_inf_ = 0.62) was not significantly different from the FIR STRF (jackknifed *t*-test). Thus within the precision we could achieve with this analysis, both models captured the essential features of the FIR STRF. Error bars on asymptotic performance are relatively large, especially for the FIR STRF, so a strong conclusion about relative performance of these models is difficult. However, asymptotic performance of the P3Z1x1 model was significantly worse than the other models (*R*_inf_ = 0.56, *p* < 0.001), indicating a failure of this very simple model to capture the full linear model.

For comparison with a previous analysis [22], we also measured asymptotic performance for the FIR STRF with no output nonlinearity. This model performed better than the standard FIR STRF for smaller estimation sets, presumably due to its reduced complexity, but its advantage diminished for larger datasets. Asymptotic performance was slightly lower than the standard FIR STRF that included an output nonlinearity (*R*_inf_ = 0.61, *p* < 0.05, Fig 8B).

### Additional benefits of STRF parameterization

In addition to outperforming the FIR model in finite data conditions, reduced-dimensionality factorized and parameterized STRFs demonstrated several other benefits over the FIR STRF, which we detail below. For brevity in this section, *factorized model* refers specifically to the *D* = 2 factorized model, and *parameterized model* refers to the *D* = 3 Gaussian spectral parameterization with P3Z1 temporal parameterization. These models were chosen because they represent the best-performing models, respectively, among the factorized and parameterized models tested (Fig 6C).

#### Parameterization permits higher spectral and temporal resolution STRFs

**Fig 9:**
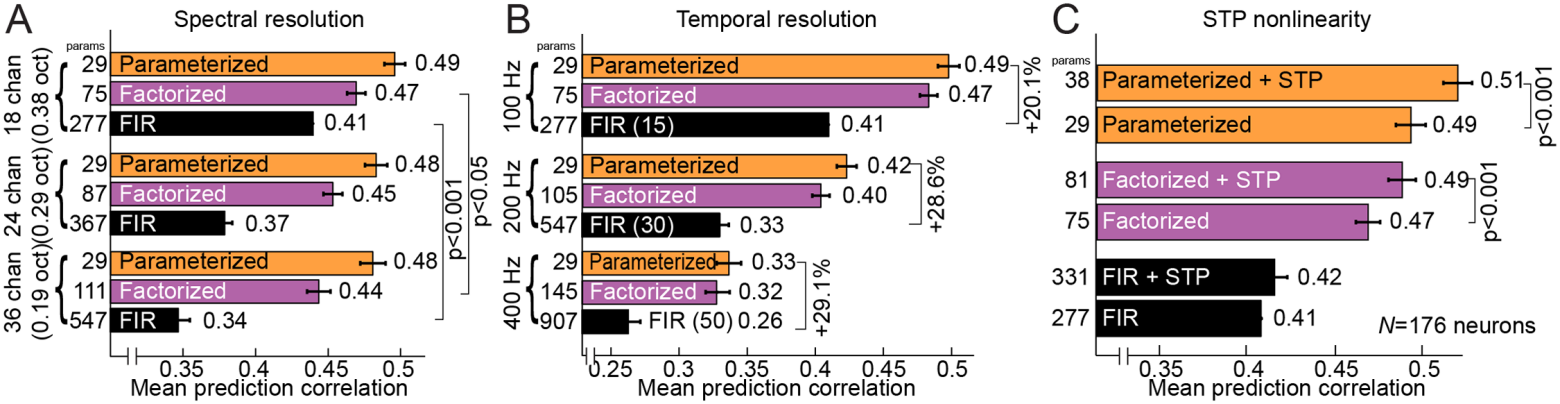
Effects of spectral and temporal resolution on model performance. **A.** As the number of cochlear filterbank channels increases from *C* = 18 to 24 or 36, there is no significant change in performance of the parameterized model. However, performance of the factorized and FIR models is significantly decreased (sign test). **B.** As temporal resolution is increased from 100 to 200 or 400 Hz, the average prediction correlation of all models decreases. However, FIR model performance degrades more than the factorized and parameterized models. Numbers in parentheses denote the number of temporal bins *U* used. **C.** Average performance of models with and without nonlinear short-term plasticity (STP) incorporated into the STRF. STP does not significantly improve performance of the FIR STRF, but it does improve performance of the factorized and parameterized models.

The dimensionality of the smooth, continuous parameterized basis functions is independent of spectral and temporal sampling resolution, similar to spline basis functions used in other encoding models [29, 33]. Thus, unlike the FIR models, these models do not require more parameters as spectral or temporal granularity is increased. To test the impact of increasing resolution, we compared performance of the FIR, factorized and parameterized models for increasing spectral (24 or 36 channels instead of 18) and temporal resolution (200- or 400 Hz sampling instead of 100 Hz). At higher spectral resolution, performance of the FIR models decreased, reflecting greater estimation noise from the larger number of parameters (*p* < 0.001, sign test, Fig 9A). At the same time, performance of the parameterized models remained stable with higher spectral resolution.

Increasing temporal resolution decreased performance of all models, as expected for the greater temporal bandwidth of the predicted PSTH [45] and the tuning of some A1 neurons for fast temporal modulations [27]. The general decrease in performance may also reflect fast temporal nonlinearities such as the spike refractory period that are incorporated into some implementations of the GLM [14, 31]. Although performance decreased for all models, the relative decrease for the FIR model was greater than for the others (Fig 9B). Thus the parameterized models are consistently less sensitive to effects of increased temporal resolution.

#### Parameterization improves performance of models with additional nonlinear terms

Parameterization need not be limited to the *linear* STRF. By minimizing the number of parameters required to account for linear response properties, this strategy preserves statistical power for incorporating additional *nonlinear* terms. To test the feasibility of adding nonlinear terms to the parameterized model, we incorporated a module that mimicked the effects of short-term synaptic plasticity on each spectral channel prior to temporal filtering (STP, Fig 1F, [52]). This architecture permits distinct, nonlinear adaptation for each spectral channel and may explain some properties of stimulus-specific adaptation [53]. Incorporating nonlinear STP into encoding models improves prediction accuracy for responses to narrowband noise stimuli [30], and we expected that it would also improve model performance for the more spectrally complex vocalizations.

In our general model framework, STP is incorporated by inserting an extra module into the sequence of transformations that maps input stimulus to output spike rate (Eq 1). The STP module mimicked the effects of short-term depression and/or facilitation prior to the linear temporal filter (Fig 1F). Each STP synapse was specified by three free parameters: baseline presynaptic activity level (in the absence of an auditory stimulus), probability of vesicle release and rate of vesicle recovery (Eq 17). We first tested the effect of incorporating STP into the FIR STRF. For the *C* = 18 channel FIR, STP required 54 additional parameters, but it did not significantly improve performance (Fig 9C), presumably because the many additional parameters lead to overfitting. However, adding STP to the factorized and parameterized models did significantly improve predictions (Fig 9C, parameterized model: *R* = 0.510 vs. 0.485, *p* < 0.001, sign test). These results suggest that the STP module introduced useful new degrees of freedom, reflecting properties of A1 neurons that are not captured by the linear model.

#### Parameterization tolerates unreliable sensory responses

In auditory cortex, the reliability of auditory-evoked responses varies from neuron to neuron [22, 45]. Thus for a fixed data set size, such as the vocalization data in the current study, the amount of information available for measuring sensory coding properties is also variable across neurons. To account for differences in response reliability, we computed a signal-to-noise ratio for each neuron, based on the variability of single-trial responses to repetitions of the same vocalization (SNR, Eq 21). The SNR provided a simple measure of the fraction of single-trial neural activity that could be accounted for by the stimulus.

**Fig 10:**
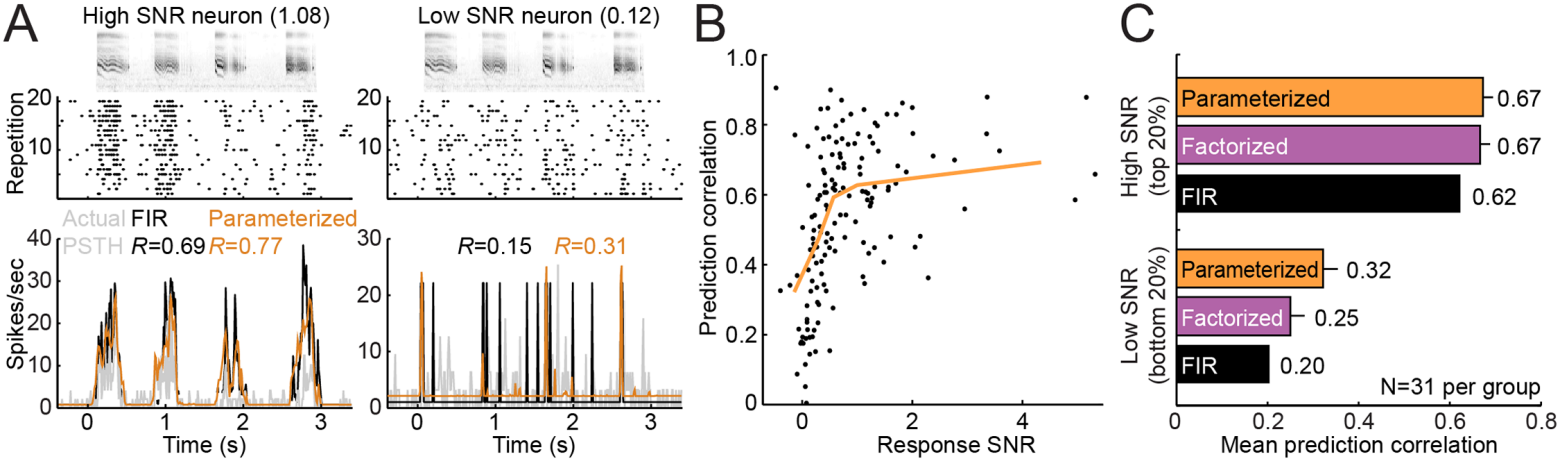
Model performance versus neuronal signal-to-noise. **A.** Example data for two neurons with high (left) or low (right) response SNR. A validation stimulus (spectrogram, top) was presented over 20 repetitions (raster response, middle). Both the FIR and parameterized STRFs were able to predict activity of the high-SNR neuron more accurately than the low-SNR (predicted versus actual PSTHs, bottom). However, the relative improvement for the parameterized STRF was greater for the low-SNR neuron (prediction correlations shown, *R*). **B.** Scatter plot compares signal-to-noise ratio (SNR, estimation data) against prediction correlation (validation data) by the parameterized model for each A1 neuron. Orange line plots the average for data grouped in quintiles according to SNR, showing a correlation (*R* = 0.45). **C.** Average prediction correlation for the FIR, factorized, and parameterized models separately for top and bottom quintile of neurons, ranked by response SNR. The factorized and parameterized models show greater improvement over the FIR model for the low-SNR neurons than the high-SNR neurons.

Neurons with low SNR were more susceptible to fitting error and produced STRFs with lower predictive power (Fig 10A). We measured prediction correlation (validation data) as a function of SNR (estimation data) and found a positive correlation (Fig 10B). We expected noise from overfitting to be particularly severe for models such as the FIR STRF that require a large number of parameters. To test this prediction, we sorted neural data according to SNR and compared performance of the FIR, factorized and parameterized models in the top and bottom quintiles (Fig 10C). In the top quintile, performance of all three models was nearly the same, and the FIR model performed only slightly worse than the others (*p* < 0.05, sign test). In the bottom quintile, all models performed worse, but the average prediction correlation for the parameterized model was about 50% greater than the FIR model (*p* < 0.0001, sign test). Thus when SNR is low, the FIR model is particularly susceptible to overfitting compared to parameterized models.

### Broader applicability

#### Results are not specific only to a brain region or stimulus

**Fig 11:**
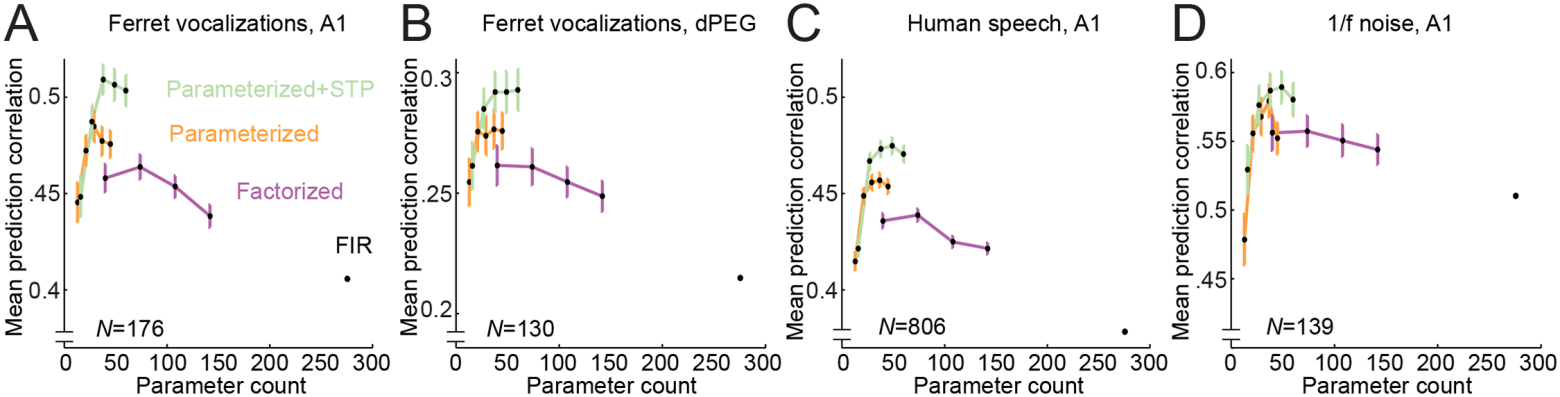
Comparison of model architectures across brain areas and stimulus domains. **A.** Pareto plot summarizes performance of the FIR, factorized, parameterized and parameterized-STP models for A1 vocalization data. Error bars indicate SEM for the difference from performance by the FIR STRF, as in Fig 4C. **B.** Comparison of model performance for vocalization data recorded in ferret belt auditory cortex (dPEG), plotted as in A. Overall performance is lower than in A1, but performance rankings are the same: *D* = 2 factorized > FIR model (*p* < 0.001, sign test), *D* = 3 parameterized > *D* = 2 factorized model (*p* < 0.01), *D* = 3 parameterized-STP > *D* = 3 parameterized model (*p* < 0.001). Thus results of the model comparison generalize across areas in the auditory processing hierarchy. **C.** Comparison of model performance for human speech data recorded in A1. The parameterized-STP models again show the strongest performance with successive significant improvements for each model architecture (*p* < 0.001, sign test, for all comparisons), indicating that the results generalize across different natural stimuli. **D.** Comparison of model performance for 1/*f* noise data recorded in A1. The models again show the same pattern of relative performance, indicating that the results also generalize to synthetic noise stimuli.

Because of the large number of model comparisons in the current study, our best parameterization could be overly specific to the A1 vocalization dataset (Fig 11A). The question remains whether the performance of the factorized and/or parameterized models generalizes to different brain areas and different stimuli. To test for generalization across brain areas, we compared model performance on data collected with the same vocalization stimuli from a secondary (belt) auditory cortical area (dorsal posterior ectosylvian gyrus, dPEG [54, 55]). Overall, prediction correlation was lower than for A1, as expected for a more central brain area (Fig 11B). The dimensionality of the best-performing models also differed slightly. However, the factorized and parameterized models again showed the same pattern of improved performance over the FIR STRF.

To test for generalization across stimuli, we compared model performance for a dataset collected from a different population of A1 neurons during presentation of continuous human speech (data reanalyzed from [9]) and during presentation 1/*f* spectro-temporal noise [56]. For the noise stimulus, first order spectral and temporal modulation power spectra were matched to natural stimuli, but higher order correlations were not. As in the case of vocalizations, we observed a pattern of greater prediction accuracy by the parameterized and factorized STRFs in both datasets (Fig 11C and 11D). In the case of 1/*f* noise, relative differences in model performance were not as large as for the natural stimuli, possibly because the noise did not contain as many sharp onsets as vocalizations. Despite the quantitative differences, these results confirm that the improved performance of the low-dimensional models is a general property across auditory cortical areas and across stimulus conditions.

#### Parameterized models provide more direct measures of neural circuit properties

**Fig 12:**
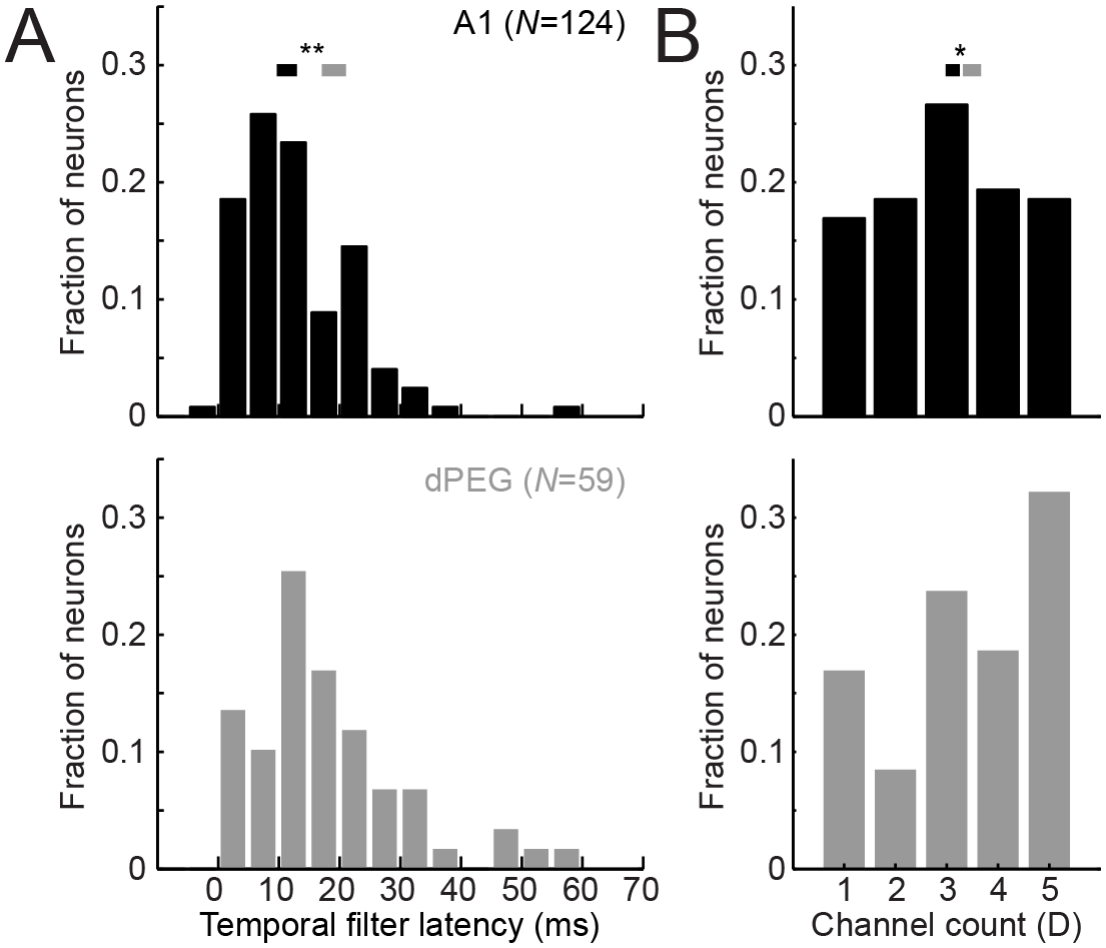
Direct interpretation of parameterized models. **A.** Histogram compares latency measured directly from the P3Z1 temporal filter for neurons in A1 and dPEG. Mean response latency is shorter in A1 (12.6 ms) than in PEG (17.9 ms, *p* < 0.001, Wilcoxon rank-sum test, *N* = 124 neurons with SNR > 0.005). **B.** Histogram of optimal spectral channel count for neurons in A1 and dPEG. Neurons were classified according to the spectral channel count (*D* = 1…5) of the P3Z1 parameterized model that produced the best prediction in the validation data. Mean optimal channel count in A1 (3.04) was lower than in dPEG (3.41, *p* < 0.05, rank-sum test)

Any encoding model captures information about function of the underlying biological circuit, but extracting this information is more straightforward for parameterized models. Parameterized model fits provide direct measures of sensory tuning such as response latency (*l* in Eq 10) and spectral tuning bandwidth (*σ* in Eq 7). We compared response latencies between A1 and dPEG for the parameterized model and found longer average response latency in dPEG (*p* < 0.001, Wilcoxon rank-sum test, Fig 12A). The tendency toward longer response latency in belt versus core fields is consistent with previous reports [54, 55]. This difference is typically attributed to the greater number of synapses (and associated delays) required for auditory signals to reach belt than core areas. However, nonlinearities in the temporal response can also shift response latency in the linear STRF, which may explain why some latencies in the current results are shorter than the minimum of 10–15 ms typically reported for cortex [30]. Thus differences between A1 and dPEG could reflect nonlinear response properties in addition to differences in accumulated synaptic delays.

The structure of parameterized models can also be studied at a higher level to assess the general complexity of STRFs. To illustrate this idea, we determined the number of spectral channels, *D* = 1 to *D* = 5, that produced the best-performing parameterized model for each neuron in A1 and PEG. The average best number of spectral channels is *D* = 3.04 for A1 and *D* = 3.41 for dPEG (Fig 12B, *p* < 0.05, rank-sum test). Optimal channel counts were determined from performance on validation data only. Thus this effect is not biased by the lower SNR typically observed in dPEG relative to A1 [55]). Instead, the larger average channel count indicates greater complexity in dPEG STRFs and that encoding models require greater degrees of freedom in dPEG than in A1. Identifying optimal channel counts also provides a form of relevancy determination that groups model parameters logically (*i.e.*, entire spectro-temporal dimensions) for inclusion or exclusion, rather than individual parameters of a much larger model [38, 46].

In practice, properties such as response latency and optimal spectral channel count can also be measured from FIR STRF coefficients [9]. However, this procedure requires a second stage of analysis in which tuning properties must be fit to the FIR parameters, an approach that requires more involved analysis and is susceptible to the effects of noise in the FIR parameter estimates. For a parameterized model, these properties are fit in a single procedure that maximizes predictive power. Either approach makes assumptions about tuning properties that may introduce bias their measurement, but that bias is made explicit in a single operation for a parameterized model rather than in the multiple stages of fitting and feature extraction required for more complex models.

#### Parameterized model performance holds under alternative prediction accuracy metrics

**Fig 13:**
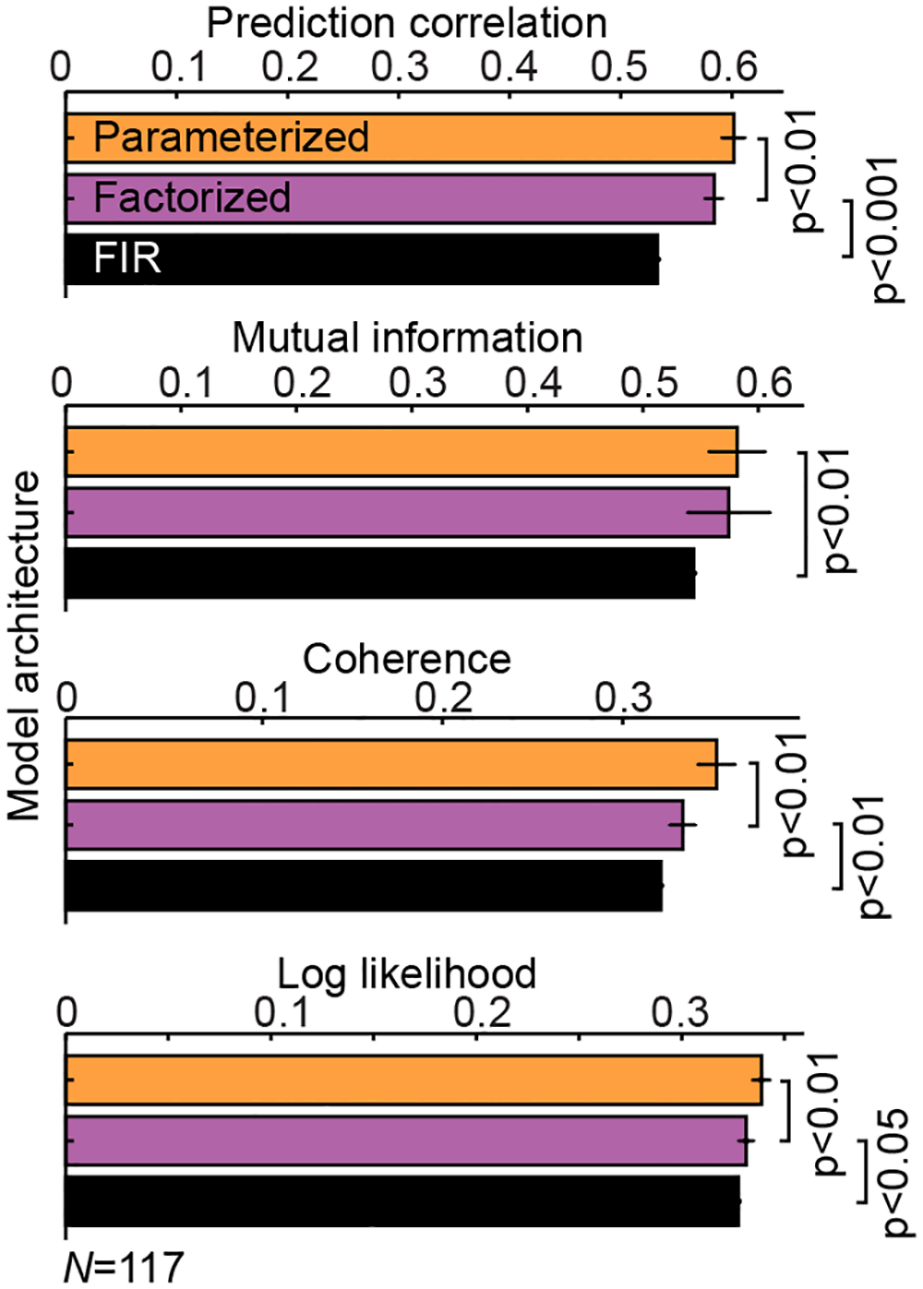
Different performance metrics yield the same model ranking. Average performance of the FIR, factorized and parameterized models evaluated using four different metrics of prediction accuracy. All four metrics produce the same rankings of relative model performance. Data are shown for *N* = 117 neurons with SNR > 0.01 because some performance metrics were susceptible to noise for low-SNR neurons.

Finally, a number of alternative metrics have been proposed for evaluating the performance of receptive field models, including mutual information (MI) [57], mean coherence (closely related to mutual information) [45], and negative log likelihood (NLOGL, maximum likelihood for a Poisson-spiking noise model) [14, 15, 29]. We compared the performance of the FIR, factorized and parameterized models according to these different metrics (Fig 13). There was some variability in scores across individual neurons, but the relative performance of the different models was largely consistent for all the metrics, particularly the improved performance of the parameterized model over the FIR.

## Discussion

### Reduced complexity of auditory encoding models without reduced performance

The finite impulse response (FIR) STRF represents the current standard model for stimulus-response filtering in the auditory system [2, 4, 8–13]. Our results agree with previous findings that, as a general architecture, the linear STRF accounts only partially for the neural response to natural sounds in A1 [9, 23]. However, we find that the same level of performance can be achieved by much simpler models. A model requiring fewer than 30 parameters not only matches performance of the FIR STRF (> 250 parameters) but actually outperforms it for large but finite datasets. The simplest parameterization that works optimally for a neural population provides insight into the neural circuitry underlying system function [36]. According to this logic, the average linear STRF of an A1 neuron can be captured by the sum of three channels with Gaussian spectral tuning and an IIR temporal filter.

When data are finite, a critical issue is that a simpler model with fewer free parameters will be less susceptible to estimation noise than a more complex model. Thus the simpler model may perform better, even if it fails to account for important degrees of freedom in the more complex one. Accounting for the impact of estimation noise on model performance is difficult, as it requires extrapolation to the condition where data are infinite [18, 22]. By assuming that estimation noise is additive, we found that a simple inverse relationship between estimation set size and prediction error accurately described performance for several different architectures (Fig 8A). In the limit of infinite data and under these assumptions, the FIR STRF did not perform significantly better than the simple parameterized model. These results should be confirmed with a larger dataset, but the current analysis suggests that the essential degrees of freedom for the linear STRF are much closer to 29 than to the 276 specified by the FIR STRF.

The average linear STRF in A1 may be described by about 30 parameters, but STRFs for individual neurons do vary substantially in their complexity. Some neurons require only one spectral channel for optimal performance while others require four or more channels (Fig 12B). The fact that only a minority of neurons were best described by a single dimension argues that most linear STRFs are not frequency-time separable [37, 51]. At the other extreme, even STRFs with four or five spectral channels required substantially fewer parameters than the standard FIR STRF.

This low dimensionality generalizes across other natural and synthentic stimuli in A1, but our analysis of data from the belt area dPEG indicates that more complex models are required for non-primary cortex (Fig 11). Moreover, even in A1, the full dimensionality of encoding models is likely to be greater than what is required to specify the linear STRF. As demonstrated by the enhanced performance of the nonlinear STP STRFs (Fig 11), introducing additional dimensionality that extends outside of the linear STRF architecture can improve model performance.

### Upper bounds on linear STRF performance

How well can the linear STRF actually describe sensory responses in A1? Issues surrounding finite sampling of experimental data again make it difficult to answer this question definitively [18, 45]. After implementing our estimation noise model, we found that the FIR STRF is able to account for 40% of A1 response variance on average (*i.e.*, variance explained is 100*R* ^2^ for *R* = 0.63, Fig 8). Factorized and parameterized STRFs very nearly matched performance of the FIR model (39% of response variance), indicating that these approximations capture the essential features of the more complex model, despite requiring only about 50% and 10% of the parameters, respectively. These measurements establish baseline performance by the linear STRF that must be surpassed by any more accurate model. At the Pareto frontier, a better model must either produce more accurate predictions or require fewer parameters and perform as well.

Only one previous study has attempted to answer this question rigorously, using activity driven by random chord stimuli in anesthetized mice [22]. Although we focused primarily on models that included an output nonlinearity [13, 14], we also computed asymptotic performance of STRFs without this nonlinear term in order to make a more direct comparison to the previous analysis of asymptotic performance. Without a spiking nonlinearity, the average FIR STRF was able to account for about 37% of response variance. This result falls in the range of 18–40% reported previously [22], although several factors make a direct comparison difficult. In the current study, recordings were performed in awake ferrets and used natural vocalizations rather than anesthetized mice and noise stimuli. Anesthesia can impact auditory neural activity [58, 59], and natural sounds evoke nonlinear response properties in a different functional domain than noise stimuli [9, 60].

The number of models tested here was relatively large, but they are still likely to be suboptimal compared to as-yet-untested parameterizations. The current study explored only two spectral parameterizations (Gaussian and Morlet functions) and the pole-zero family of IIR temporal filters. Numerous other basis functions could be considered, including Gabor wavelets [42, 61] or empirically-derived basis functions [29, 31, 33]. There is a clear trade-off between basis function complexity and the number of spectral dimensions needed. Better-performing temporal kernels like the P3Z1 filter reach their peak performance when *D* = 3, while simpler kernels like P1Z0 need *D* ≥ 4 to reach the same performance. Thus the interaction between channel count and basis function complexity will be relevant to identifying optimal parameterizations.

### Parameterization supports exploration of nonlinear and state-dependent models

The efficiency of estimating parameterized STRFs allows the introduction of new, nonlinear terms that can account for encoding properties that are not captured by the linear model [30, 31]. When nonlinear short-term plasticity was introduced to the FIR STRF, it did not change model performance, but when it was introduced to the parameterized model, it improved predictive power. Thus the benefits of nonlinear terms may only become apparent when sufficient statistical power is available in the current dataset.

The family of models used in this study incorporate static nonlinearities that are commonly part of STRFs. This include log-compression of the input spectrogram to account for basilar membrane mechanics [25, 26] and an output nonlinearity to account for spike threshold and saturation [13, 14]. Other studies have incorporated nonlinear terms into the core computation of the filter. Some use general Volterra series expansions to account for second- and higher-order nonlinearities [3, 27, 57, 62]. Others incorporate more specific terms aimed at capturing contextual influences [28, 29] or mimicking biological circuit elements [26, 31]. These additional nonlinear terms can be incorporated into the parameterized framework, potentially providing substantial improvements in predictive power.

Neurons also undergo plasticity at multiple timescales due to stimulus context [12, 30, 63, 64], changes in behavioral state [50, 65, 66], and learning [49, 67]. In many experimental settings, the quantity of data available in a single behavioral state may provide a critical limitation on statistical power. Low-dimensional parameterized models may be particularly beneficial for exploring changes in spectro-temporal response properties in these experimental settings.

### Parameterization as regularization

From a general analytical perspective, parameterization is similar to regularization during model estimation [1, 12, 46, 68]. In both cases, pre-existing knowledge or a hypothesis about the system’s function is used to constrain model fits. The idea that sensory receptive fields should vary smoothly in space and time has motivated the use of priors for smoothly varying STRFs [46, 68]. Similarly, the idea that receptive fields should have a relatively small number of non-zero parameters has motivated a sparse prior on model fits [14, 46]. Constraining the STRF to have analytical form of the factorized or parameterized models serves the same purpose of imposing a prior on the fit [38]. In the current study, the spectral and temporal parameterizations constrain both sparseness (limiting the model’s degrees of freedom) and smoothness (Gaussian spectral tuning and exponential temporal tuning).

To simplify model comparisons in this study, we used a single fit algorithm across all models. Thus it was not optimized specifically for the FIR STRF. Incorporating stricter stop criteria and sparseness constraints improve FIR STRF performance, but even after tuning the cost function, it did not match the performance of the parameterized model. The factorized and parameterized models were less sensitive to details of the fit algorithm such as the stop criterion, emphasizing the benefits of regularization effectively built into parameterization.

### Pareto fronts describe optimal trade-offs in model performance

Most real world optimization problems involve the simultaneous minimization of several objectives [69]. Thus when comparing different model architectures, it may be helpful to consider trade-offs separately along different dimensions [36, 70, 71]. The current study focused in particular on the trade-off between model prediction accuracy and parameter count. In general, however, such an approach can be used to define an *N*-dimensional Pareto front containing the best models according to numerous other measures, including alternative performance metrics (Fig 13, see also [72]), alternative model complexity metrics [73, 74], data required to fit (Fig 8), computational cost [75], or model plausibility [76].

Pareto fronts are extensively used in the context of multiobjective optimization for the formulation of heuristics [69]. Given the complexity of performing a search on the space of model architectures, we relied here on inspection of the Pareto front to guide model design. While developing new analytic models to test, we found it most helpful to generate new models by adding to or discarding from a model on the current Pareto front. Variants of non-Pareto-optimal models rarely improved performance or provided insight into the relevancy of new parameters. Of particular note, the FIR implementation falls far from the Pareto front (Fig 2B), making it difficult to test variants based on the FIR STRF.

## Methods

### Experimental procedures

Single-unit neural activity was recorded from five awake, passively listening ferrets. For the main analysis of responses to vocalizations, a total of 176 single units were recorded in primary auditory cortex (A1) and 130 units in belt auditory cortex (dPEG). For one analysis (Fig 11C and 11D), responses were analyzed for 808 A1 units recorded during the presentation of continuous speech (reanalyzed from a previous publication [9]) and for 139 A1 units recorded during the presentation of 1/*f* noise [56].

Data used in this study will be made publicly available online via the Neural Prediction Challenge (http://neuralprediction.berkeley.edu/).

#### Neurophysiological recordings

Prior to experiments, animals were implanted with a custom steel head post to allow for stable recording. While under anesthesia (ketamine followed by isoflurane) and under sterile conditions, the skin and muscles on the top of the head were retracted from the central 4 cm diameter of skull. Several stainless steel bone screws (Synthes, 6 mm) were attached to the skull, the head post was glued on the mid-line (3M Durelon), and the site was covered with bone cement (Zimmer Palacos). After surgery, the skin around the implant was allowed to heal. Analgesics and antibiotics were administered under veterinary supervision until recovery.

After animals fully recovered from surgery and were habituated to a head-fixed posture, a small craniotomy (1–2 mm diameter) was opened over A1. Neurophysiological activity was recorded using tungsten microelectrodes (1–5 M*Ω*, A.M. Systems). One to four electrodes positioned by independent microdrives (Alpha-Omega Engineering EPS) were inserted into the cortex. Electrophysiological activity was amplified (A.M. Systems 3600), digitized (National Instruments PCI-6259), and recorded using the MANTA open-source data acquisition software [77]. Recording site locations were confirmed as being in A1 based on tonotopy, relatively well-defined frequency tuning and short response latency [11].

Spiking events were extracted from the continuous raw electrophysiological trace by principal components analysis and *k*-means clustering [9]. Single unit isolation was quantified from cluster variance and overlap as the fraction of spikes that were likely to be from a single cell rather than from another cell. Only units with > 80% isolation were used for analysis.

Stimulus presentation was controlled by custom software written in Matlab (version 2012A, Mathworks). Digital acoustic signals were transformed to analog (National Instruments PCI-6259) and amplified (Crown D-75a) to the desired sound level. Stimuli were presented through a flat-gain, free-field speaker (Manger) 80 cm distant, 0-deg elevation and 30-deg azimuth contralateral to the neurophysiological recording site. Prior to experiments, sound level was calibrated using to a standard reference (Brüel & Kjær). Stimuli were presented at 60–65 dB SPL.

#### Auditory stimuli

For most experiments, stimuli used for estimation were ferret vocalizations, which were recorded in a sound-attenuating chamber using a commercial digital recorder (44-KHz sampling, Tascam DR-400). Recordings included infant calls (1 week to 1 month of age), adult aggression calls, and adult play calls. No animals that produced the vocalizations in the stimulus library were used in the current study. Neural activity was recorded during 4–6 repetitions of 40 randomly ordered 3-second stimuli, used for model estimation, and during 20 repetitions of two additional 3-second stimuli, used for model validation.

Because validation data always used the same two vocalization sequences, one possible concern is that the vocalization results might not generalize to other stimuli. To test whether the improved performance of reduced-parameter models generalized across stimulus conditions, model performance was also compared for A1 neural activity recorded during presentation of continuous human speech. The stimulus consisted of 4–5 repetitions of 30 3-second sentences from the TIMIT library [78], each uttered by a different speaker. Data for 28 sentences were used for model estimation, and data from the remaining two were used for validation. Activity was recorded from a different set of A1 neurons during presentation of continuous 1/*f* noise. The noise was generated by computing the spectrogram of a white noise signal, low-pass filtering the spectrogram to impose temporal and spectral modulations matched to natural stimuli, and then inverting the spectrogram into a time-varying acoustic signal [56]. Neurophysiological recording techniques and the experimental protocol were identical to those used for vocalizations, except the speech stimuli were presented through a calibrated, closed-field earphone (Etymotic ER2) contralateral to the recording site. Data collected using the speech stimulus have been published previously [9].

### Receptive field model framework

The relationship between the time-varying input auditory stimulus, *x*(*t*), and simultaneously recorded single-unit firing rate response, *y*(*t*), is described by the spectro-temporal receptive field (STRF [8, 9, 11, 12]) or, more generally, any function that maps *x* to *y*. In the current study, this mapping was cast as a sequence of functional modules, in which each function was applied to the output of the previous one (Eq 1, Fig 1C). The series of functions maps roughly to the physical elements that transmit auditory information to cortex. A detailed list of all models tested in this framework is included in S1 Table.

#### Cochlear filterbank

The first stage of each STRF consisted of a second-order gammatone filterbank that modeled spectral processing in the a cochlea [44]. The frequency domain transfer function, *G*_*i*_, for the *i*th filter (*i* = 1…*C*) was parameterized in terms of quality factor *Q* and center frequency *f*_*i*_:

$$\begin{array}{c} G_{i}\left(s\right)=\frac{K}{{\left[s^{2}+\left(\frac{\omega_{0}}{Q}\right)s+2\pi f_{i}\right]}^{4}} \end{array}$$ (11)

All filters had fixed *Q*:

$$\begin{array}{c} Q={\left({\left(\frac{f_{\text{high}}}{f_{\text{low}}}\right)}^{\frac{1}{C-1}}-1\right)}^{-1} \end{array}$$ (12)

For most analyses, the filterbank included *C* = 18 filters with *f*_*i*_ spaced logarithmically from *f*_low_ = 200 to *f*_high_ = 20,000Hz.

The complex output of each gammatone filter was transformed into a positive, real signal by taking the absolute value of its Hilbert transform. The signal was then smoothed and downsampled to match the temporal bin size of the PSTH, usually 100 Hz, but sometimes 200 or 400 Hz. We found that the second-order filters used here produce models with better prediction accuracy than the classical gammatone (S1 Table, [44]). However, it is likely that more detailed cochlear models would further improve performance [26].

To account for nonlinear level sensitivity in the auditory periphery, each spectrogram channel was then passed through a logarithmic compressor (LOG*n*), which required a single free parameter, *ϕ*_1_, that determined the amount of compression,

$$\begin{array}{ccc} y_{\text{LOG}n}\left(t\right) & = & \log\left(x\left(t\right)+\phi_{1}\right) \end{array}$$ (13)

The gammatone filterbank itself did not require any additional free parameters for model estimation.

#### Linear filter

The core of STRF models is the linear filter that performs a weighted sum of the spectrogram over frequency and time (Fig 3, [8, 9, 11, 12]). The details of the different formulations used in the current study are provided in the Results.

#### Output nonlinearity

After linear filtering, a static sigmoid nonlinearity produced a prediction of instantaneous spike rate that accounted for spike threshold and saturation. The spike nonlinearity was modeled as a double-exponential (DEXP) and required four parameters, *ϕ*_1 − 4_,

$$\begin{array}{c} y_{\text{DEXP}}\left(t\right)=\phi_{1}+\phi_{2}e^{-e^{\phi_{3}\left(x\left(t\right)-\phi_{4}\right)}} \end{array}$$ (14)

As in the case of the cochlear model, a large number of alternative spike nonlinearities are possible [13, 17]. Our priority for the current study was to keep the output nonlinearity constant across all variants tested for the linear filter. The double-exponential performed as well as other sigmoid functions and proved consistently stable in the fitting algorithm used here. A complete list of output nonlinearities tested is included in S1 Table.

#### Nonlinear short-term plasticity (STP)

To test performance of a nonlinear architecture with theoretically broader explanatory power than the linear STRF, we incorporated a new module prior to the temporal filtering module (Fig 1F). Each spectral channel (either output from the cochlear filterbank or from a spectral filterbank), provided input into a simulated synapse that could undergo either depression or facilitation [30, 52]. In this simple model, the number of presynaptic vesicles available for release is dictated by the fraction of vesicles released by previous stimulation, *ν*, and a recovery time constant, *τ*. For depression, *ν* > 0, and the fraction of available vesicles, *d*(*t*), is updated,

$$\begin{array}{c} d_{i}\left(t\right)=d_{i}\left(t-1\right)-\nu s_{i}\left(t-1\right)d_{i}\left(t-1\right)+\frac{1-d_{i}\left(t-1\right)}{\tau } \end{array}$$ (15)

For facilitation, *ν* < 0, and *d*(*t*) is updated,

$$\begin{array}{c} d_{i}\left(t\right)=d_{i}\left(t-1\right)-\nu s_{i}\left(t-1\right)\left[2-d_{i}\left(t-1\right)\right]+\frac{1-d_{i}\left(t-1\right)}{\tau } \end{array}$$ (16)

The input to the synapse is scaled by the fraction of available vesicles and output to the next module,

$$\begin{array}{c} s_{di}\left(t\right)=d_{i}\left(t\right)s_{i}\left(t\right) \end{array}$$ (17)

### Model estimation and validation

For most models, stimulus and response data were binned at 10 ms (100 Hz) and averaged across repetitions. Stimulus binning was applied after transformation to the spectrogram.

Data recorded from each neuron were divided into two subsets, one used only for model estimation (4–6 repetitions of 40 3-sec vocalization sequences) and the other for validation (20 repetitions of 2 3-sec sequences). Model parameters were fit using an iterated, greedy version of boosting that minimized mean-squared error prediction of the neural PSTH in the estimation dataset (details below). Each model was then evaluated based on its ability to predict the time-varying PSTH response in the reserved validation data set. Prediction accuracy was measured as the correlation coefficient (Pearson’s *R*) between the predicted and observed PSTH [12, 34]. The correlation coefficient provides a useful metric because it scales performance between 0 (completely random) and 1 (perfect correlation). Model performance can be variable across single neurons. Thus to compare models we focused on average performance across the entire set of neurons studied, using the nonparametric Wilcoxon signed rank test (sign test) to assess significant differences in performance. Error bars for average prediction correlation plots were computed on the difference between prediction correlation for each model and the FIR STRF fit to the same neuron. Computing error bars based on the difference per neuron removed variability in overall neural response SNR (*e.g.*, Figs 4A and 10B) and revealed model differences commensurate with the sign test.

#### Signal-to-noise ratio (SNR) of neural sensory responses

Because neural responses vary across repeated stimulus presentations, some fit error resulted from uncertainty in the response in the estimation data set [22]. Therefore, we evaluated the repeatability of each neuron’s response by computing a signal-to-noise ratio. We assume that the neural noise is additive so that the response for trial *i*, is

$$\begin{array}{c} r_{i}\left(t\right)=r_{\text{actual}}\left(t\right)+\epsilon_{i}\left(t\right) \end{array}$$ (18)

and the total response variance is the sum of the actual response and noise variance, $\sigma_{r}^{2}=\sigma_{\text{actual}}^{2}+\sigma_{\epsilon }^{2}$. Total variance is measured as the autocovariance of the single trial response averaged across trials,

$$\begin{array}{c} \sigma_{r}^{2}={\langle \text{cov}\left(r_{i},r_{i}\right)\rangle }_{i} \end{array}$$ (19)

Because *ϵ*_*i*_ is uncorrelated between trials, the actual response variance is the covariance between trials,

$$\begin{array}{c} \sigma_{\text{actual}}^{2}={\langle \text{cov}\left(r_{i},r_{j}\right)\rangle }_{i\neq j} \end{array}$$ (20)

and SNR is the ratio of actual response variance to noise variance,

$$\begin{array}{c} \text{SNR}=\frac{\sigma_{\text{actual}}^{2}}{\sigma_{\epsilon }^{2}}=\frac{\sigma_{\text{actual}}^{2}}{\sigma_{r}^{2}-\sigma_{\text{actual}}^{2}} \end{array}$$ (21)

This simple statistic correlates strongly with how well the estimation set data can be described by linear models, and provides a means for ranking neurons according to how well they can be modeled (Fig 10).

Although we included the entire set of 176 neurons in most comparisons of prediction accuracy, we excluded a subset with SNR < 0.005 (leaving 124 high-SNR neurons) for the analysis of asymptotic behavior (Fig 8) and tuning properties (Fig 12).

#### Prediction correlation adjusted for finite sampling of validation data

The validation PSTH is also susceptible to noise from finite sampling, and the practical limit on the correlation coefficient is less than the ideal value of 1.0. The effect of this non-predictable variability can be compensated for by normalizing the measured correlation coefficient by the trial-to-trial response correlation (TTRC) [45]. If we define TTRC as the mean correlation coefficient (Pearson’s *R*) between all unique trial pairs, *i* ≠ *j*,

$$\begin{array}{c} \text{TTRC}={\langle \text{corr}\left(r_{i}\left(t\right),r_{j}\left(t\right)\right)\rangle }_{i,j} \end{array}$$ (22)

then the corrected prediction correlation is the mean correlation between the predicted and the single-trial actual response, normalized by the TTRC,

$$\begin{array}{c} \rho_{\text{norm}}=\frac{1}{\sqrt{TTRC}}{\langle \text{corr}\left(r_{i},p\right)\rangle }_{i} \end{array}$$ (23)

For very small TTRC or for small number of repetitions, this approximation can be unstable, but for the 20-repetition validation datasets in the current study, this approximation was stable, adjusting prediction scores by 3–39% (mean 20%). Importantly, applying this correction allowed for accurate measures of prediction accuracy, but it had no impact on relative model performance, as it was computed independent of the model fit.

#### Prediction correlation adjusted for finite sampling of estimation data

To account for prediction error resulting from finite sampling of estimation data, we adapted a technique applied to the visual system for linear FIR STRFs [18]. We treated the observed neural activity as the sum of three components,

$$\begin{array}{c} r\left(t\right)=r_{\text{lin}}\left(t\right)+r_{\text{res}}\left(t\right)+\epsilon_{r}\left(t\right) \end{array}$$ (24)

where *r*_lin_ is the portion of stimulus-dependent activity that can be explained by the current model architecture, with optimal STRF estimate **H**_lin_, *r*_res_ is the residual stimulus-dependent portion that cannot be explained by the model, and *ϵ*_*r*_ is stimulus-independent activity that produces trial-to-trial variability. The components *r*_res_ and *ϵ*_*r*_ cannot be predicted by the current model architecture, **H**_lin_. Thus they should have no impact on optimal model estimates and should not be correlated with *r*_lin_ in the limit of infinite data. However, for finite data, they can be correlated with stimuli by chance, introducing error in the STRF estimate, **H**_est_ = **H**_lin_ + **H**_err_, and subsequent error in STRF predictions,

$$\begin{array}{c} p\left(t\right)=r_{\text{lin}}\left(t\right)+\epsilon_{p}\left(t\right). \end{array}$$ (25)

Given this formulation of the observed and predicted response, the squared prediction correlation is

$$\begin{array}{c} R^{2}=\frac{{\langle rp\rangle }^{2}}{\langle r^{2}\rangle \langle p^{2}\rangle }=\frac{{\langle \left(r_{\text{lin}}+\epsilon_{p}\right)\left(r_{\text{lin}}+r_{\text{res}}+\epsilon_{r}\right)\rangle }^{2}}{\langle {\left(r_{\text{lin}}+\epsilon_{p}\right)}^{2}\rangle \langle {\left(r_{\text{lin}}+r_{\text{res}}+\epsilon_{r}\right)}^{2}\rangle } \end{array}$$ (26)

Angled brackets, 〈…〉, indicate taking the mean over time. For simplicity, we omit *t* from each term and assume the individual signals have mean zero (subtracting the mean from each signal has no impact on prediction correlation). If we then assume that error in the validation data, *ϵ*_*r*_, is zero (having been accounted for by Eq 23) and that correlations between *r*_lin_, *r*_res_, and *ϵ*_*p*_ are negligible (because noise is additive), then the experimentally measured prediction correlation reduces to,

$$\begin{array}{c} R^{2}=\frac{\langle r_{\text{lin}}^{2}\rangle }{\langle r_{\text{lin}}^{2}\rangle +\langle r_{\text{res}}^{2}\rangle +\langle \epsilon_{p}^{2}\rangle } \end{array}$$ (27)

For linear STRFs estimated by reverse correlation, variance of the prediction error decreases proportionally to the number of samples used for model estimation, *T* [18],

$$\begin{array}{c} \langle \epsilon_{p}^{2}\rangle \propto {∥H_{\text{err}}∥}^{2}\propto \frac{1}{T} \end{array}$$ (28)

STRFs estimated by boosting show a similar dependence on estimation dataset size [34]. For data set size *T*, prediction correlation is then

$$\begin{array}{c} R_{T}^{2}=\frac{\langle r_{\text{lin}}^{2}\rangle }{\langle r_{\text{lin}}^{2}\rangle +\langle r_{\text{res}}^{2}\rangle +A/T} \end{array}$$ (29)

where *A* is a constant reflecting the response signal-to-noise level of the neuron. As *T* approaches infinity, the noise term disappears leaving the prediction correlation for the optimal linear model in the absence of noise,

$$\begin{array}{c} R_{\text{inf}}^{2}=\frac{\langle r_{\text{lin}}^{2}\rangle }{\langle r_{\text{lin}}^{2}\rangle +\langle r_{\text{res}}^{2}\rangle } \end{array}$$ (30)

Substituting Eq 30 into Eq 29 produces a model for the impact of estimation sampling on prediction correlation [18, 45],

$$\begin{array}{c} \frac{1}{R_{T}^{2}}=\frac{1}{R_{\text{inf}}^{2}}-\frac{A}{T} \end{array}$$ (31)

We measured prediction correlation, *R*_*T*_, for models estimated using variable sample set sizes *T*, and fit eq 31 to determine the limit on prediction correlation for infinite data, *R*_inf_. Although estimation noise is not likely to be completely additive, this model provides a good fit to the data (see Fig 8).

### Fitting algorithm

Our goal was to compare the ability of different analytical model structures to describe the neural data. Ideally, the details of the fitting algorithm used to fit the different models should not be relevant to this comparison, but in practice, there is no single algorithm that can be applied to different models without some bias [1]. Thus, the best fitting algorithm and model analytical structures are not separable in practice. We tested a variety of fit algorithms (Fig 2, S1 Table), but we focused on a single algorithm that performed best, on average, across all the models tested.

The fit algorithm consisted of nested iterations through each STRF module, initially optimizing each module with a conservative stop criterion. Once all modules had converged for the current stop criterion, its value was reduced and procedure was repeated for the smaller criterion. When fitting each module, two different coordinate descent algorithms were used. For non-parameterized modules (FIR filter, factorized spectral filter, and factorized temporal filter), a standard coordinate descent algorithm was used. For the remaining, parameterized modules (including the input filterbank and spike nonlinearities), greedy coordinate descent was used. The details of the fit algorithm are as follows:

1. Remove the spike nonlinearity module.
2. Set initial stop criterion to *ϵ* = 10^−3^ (NMSE cost function ranges from 0 to 1, see below).
3. For each module,
  - If module is non-parameterized, use non-greedy coordinate descent:
    - Set initial step size for all parameters to *δ* = 1
    - For each parameter *ϕ*_*i*_ ∈ {*ϕ*_1_, *ϕ*_2_, …}, evaluate the cost function for *ϕ*_*i*_ + *δ* and *ϕ*_*i*_ − *δ*
    - If no step improves performance, reduce *δ* by 50%.
    - If any step improves performance, update the parameter that decreases the cost function the most by *δ* and increase *δ* by 10%.
    - If improvement in cost function > *ϵ*, *δ* > 10^−6^, and fewer than 10 steps have been taken, repeat iteration for this module.
  - If module is parameterized, use greedy coordinate descent:
    - Set initial step size for each parameter to *δ*_*i*_ = 1
    - For each parameter *ϕ*_*i*_ ∈ {*ϕ*_1_, *ϕ*_2_, …},
      A Evaluate the cost function for *ϕ*_*i*_ + *δ*_*i*_ and *ϕ*_*i*_ − *δ*_*i*_
      B If neither step improves the cost function, reduce *δ* by 50%.
      C If the step helped, update *ϕ*_*i*_ by *δ*_*i*_ and increase *δ*_*i*_ by 10%.
      D If improvement in cost function >*ϵ*, *δ*_*i*_ > 10^−6^, and fewer than 10 steps have been taken, repeat iteration for this parameter.
4. After all modules cease to show cost function improvements > *ϵ*, decrease *ϵ* by 30%.
5. If *ϵ* > 10^−4^ (without spike nonlinearity) or *ϵ* > 10^−6^ (with spike nonlinearity), iterate through modules again.
6. Replace spike nonlinearity and repeat all of the above, starting with step 2.

In general, we found that fitting parameters separately within modules and iterating through modules with progressively smaller stop criteria helped avoid local minima. Fitting first without the spike nonlinearity also helped avoid local minima. The greedy algorithm increased the risk of overfitting complex models, but on average greatly improved predictions for models with nonlinear and parameterized modules. The non-greedy algorithm worked best for non-parameterized modules where all parameters are of similar scale.

#### Normalized mean squared error (NMSE)

Model parameters were optimized by minimizing a cost function based on normalized mean squared error (NMSE) between the predicted and actual neural response. For a time-varying response, *r*(*t*), averaged across repetitions of the same stimulus (mean over time, $\bar{r}$), and corresponding predicted response, *p*(*t*), the NMSE is,

$$\begin{array}{c} e_{\text{MSE}}\left(p,r\right)=\frac{\sum_{(t=1}^{T}{\left(p(t)-r(t)\right)}^{2}}{\sum_{t=1}^{T}{\left(r\left(t\right)-\bar{r}\right)}^{2}} \end{array}$$ (32)

A value of *e*_MSE_ = 1 indicates a random prediction, *e*_MSE_ = 0 indicates a perfect prediction. To reduce overfitting to noise, a shrinkage factor was applied to the NMSE [18]. Shrinkage scaled the NMSE according to its reliability across the estimation dataset. Standard error on the NMSE, *σ*_MSE_, was computed by a 10-fold jackknife [79]. The final NMSE was then scaled according to reliability,

$$\begin{array}{c} e\left(p,r\right)=1-\left[\left(1-e_{\text{MSE}}\right){\left|1-{\left(\frac{\sigma_{\text{MSE}}}{1-e_{\text{MSE}}}\right)}^{2}\right|}^{+}\right] \end{array}$$ (33)

where |⋯|^+^ indicates positive rectification. Thus if *σ*_MSE_ > >(1 − *e*_MSE_), *e* was shrunk toward a value of 1 (*i.e.*, less improvement in prediction).

For linear systems with Gaussian noise, the NMSE is equivalent to Bayesian maximum likelihood (ML) optimization. Because noise in neural systems is non-Gaussian, alternative error metrics have been proposed, such as those based on a Poisson noise model [15] or mutual information [57]. Changing the cost function affected relative model performance for individual neurons, but we did not observe any systematic effect of alternative metrics across the dataset Fig 13. Other datasets may be more sensitive to the specific cost function used, but we chose to use the NMSE here for its efficiency and relative stability.

#### Signal normalization

Not shown in Fig 1B are signal normalization computations, which occur inside each arrow connecting modules. Normalization prevents signals from taking arbitrarily high values and is thus helpful to avoid numerical issues during fitting. If *x*_*i*_(*t*) is the *i*th input, then *y*_*i*_(*t*) is the positive-definite output from the normalization module,

$$\begin{array}{ccc} \mu_{i} & = & \frac{1}{N}\sum_{t=1}^{T}x_{i}\left(t\right) \end{array}$$ (34)

$$\begin{array}{ccc} \sigma_{i} & = & \sqrt{\frac{1}{N-1}\sum_{t=1}^{T}{\left(x_{i}\left(t\right)-\mu_{i}\right)}^{2}} \end{array}$$ (35)

$$\begin{array}{ccc} m_{i}\left(t\right) & = & \frac{1}{\sigma_{i}}\left(x_{i}\left(t\right)-\mu \right) \end{array}$$ (36)

$$\begin{array}{ccc} z_{i} & = & \min(m_{i}\left(t\right),m_{i}\left(t+1\right),...) \end{array}$$ (37)

$$\begin{array}{ccc} y_{i}\left(t\right) & = & \frac{1}{\sigma_{i}}\left(x_{i}\left(t\right)-z_{i}\right) \end{array}$$ (38)

We normalized each channel by its mean power only over the estimation dataset and applied these same scaling terms to predictions on the validation set. This approach did not introduce additional free parameters to the final model, as the scaling terms could be merged post-hoc into other fit parameters. However, it provided substantial benefit to performance on the validation set.

#### Fit algorithm performance

**Fig 14:**
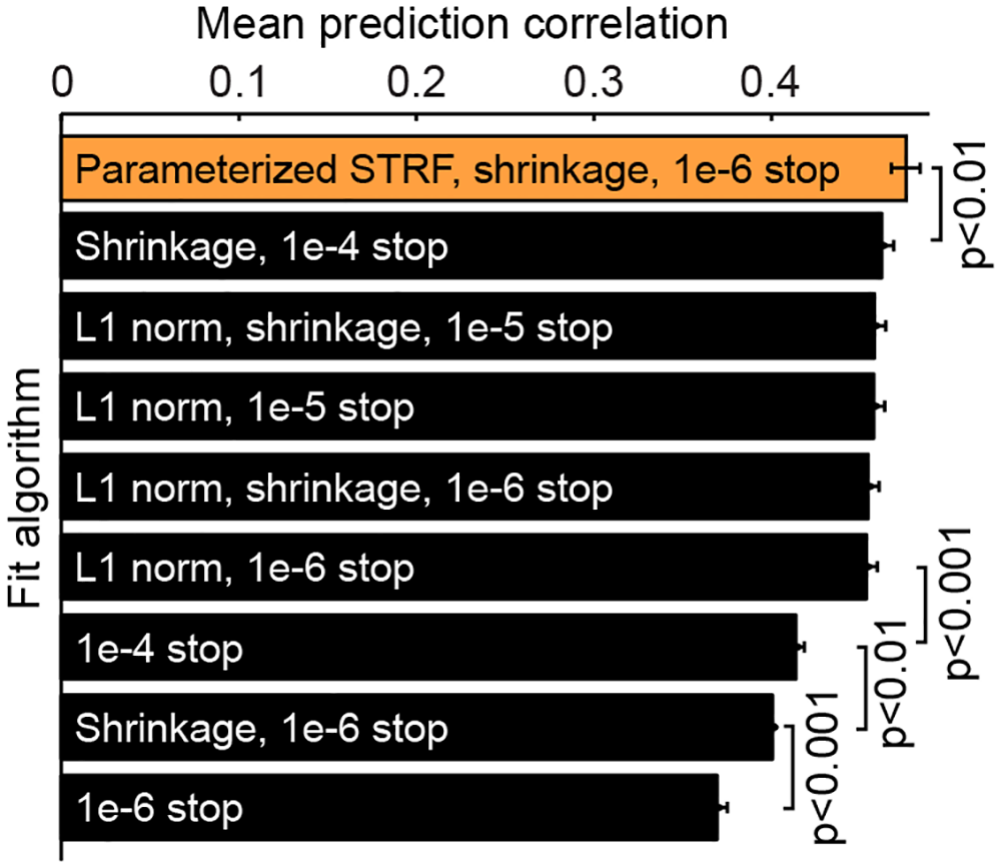
Impact of alternative regularization schemes on STRF performance. Bar chart compares mean prediction correlation of FIR STRFs estimated using alternative regularization schemes (black) to that of parameterized STRFs estimated using the standard algorithm used for the main model comparison (“shrinkage, 1e-6 stop”). Incorporating a shrinkage factors on NMSE measures (“shrinkage”), early stopping (“1e-4 stop” versus “1e-6 stop”), and an L1 norm on parameter estimates (“L1 norm” [14]) all lead to improvements in performance. The parameterized STRF still maintains greater prediction accuracy than the best regularization scheme (“Shrinkage, 1e-4 stop”).

In the current study, we used a single fit algorithm in an attempt to simplify comparisons between different model architectures. It is likely that better fit algorithms exist, particularly for the FIR STRF, where careful regularization can significantly improve model performance [12, 14, 34]. We tested several alternative regularization schemes on performance of the FIR STRF (Fig 14). Several of these alternatives produced improved performance over the standard algorithm used in this study (“shrinkage, 1e-6 stop”, defined above). Incorporating a stricter stop criterion (“1e-4 stop”), an L1 norm, or both into the cost function all produced similar improvements in performance, indicating that choosing a regularization scheme appropriate to the current model architecture is critical for optimizing performance. However, the parameterized model still performed slightly better than even the best alternative FIR STRF fits, consistent with our conclusion that the linear STRF can be approximated by a much lower dimensional architecture than the FIR STRF.

### Ethics statement

Experimental procedures were approved by the Oregon Health and Science University Institutional Animal Care and Use Committee and conformed to standards of the National Institutes of Health.

## Supporting Information

S1 Table Full list of models tested. Summary of performance for 1061 formulations of the linear STRF (see Fig 2), averaged across the *N* = 176 A1 vocalization datasets. Each row indicates parameter count, fit performance (estimation data), test performance (validation data), and test performance after correcting for validation sampling limitations (Eq 23) for a single model. The first 163 rows describe models detailed in this study, and the remainder are additional suboptimal models that were tested. The key on the first two pages indicates how to interpret model names.
(PDF)
