# Supplementary material for: The Essential Complexity of Auditory Receptive Fields
Source: PLoS Comput Biol. 2015 Dec 18;11(12):e1004628. doi: 10.1371/journal.pcbi.1004628 (PMC4684325; doi:10.1371/journal.pcbi.1004628)
Supplement: S1 Table — Summary of performance for 1061 formulations of the linear STRF (see Fig 2), averaged across the N = 176 A1 vocalization datasets. Each row indicates parameter count, fit performance (estimation data), test performance (validation data), and test performance after correcting for validation sampling limitations (Eq 23) for a single model. The first 163 rows describe models detailed in this study, and the remainder are additional suboptimal models that were tested. The key on the first two pages indicates how to interpret model names. (PDF) [file pcbi.1004628.s001.pdf]

Supplementary table: Full model listing

Key to model specifications

Model names parallel the sequence of modules described in the text (Eq. 1). Each string (delimited by underscores) corresponds to one module or fit algorithm (typically the last string in the sequence). Models detailed in the Results are listed first, followed by the entire set tested in the study.

Input filterbank (cochleogram, spanning 200-20000 Hz)

	apgt<C>x<T>	All-pole gammatone filter, C channels, T sampling rate
*	fb<C>ch<T>	Gammatone-like filterbank, 4th order, C channels, T sampling rate (Eq. 12)
	gt<C>ch<T>	Gammatone-like filterbank, 4th order, C channels, T sampling rate
	ozgf<O>fb<C>x<T>	Gammatone-like filterbank, O order, C channels, T sampling rate
	ozgf<O>x<T>	Gammatone-like filterbank, O order, 12 channels, T sampling rate
	szgf<C>x<T>	Two-zero gammatone-like filterbank, 4th order, C channels, T sampling rate
	smrav	Smooth estimation data spike rate in time (std dev=1 bin)

Static nonlinearities (input compression or spike nonlinearity)

	combaverage	Average spectral channels
	combpoly2	Combine spectral channels via second-order polynomial
*	dexp	Asymetric double exponential (Eq. 14)
	dexp3b	Asymetric double exponential with fixed amplitude (Eq. 14, phi_2=20)
	dlog	Natural log
	dlogb	Variable-base log with zero threshold
	dlogbz	Variable-base log with variable threshold
	exp	exp[phi_1 * (x-phi_2)]
	invlin	phi_1 + phi_2 * [1- 1/x]
	invlinz	phi_1 + phi_2 * [1- 1/(phi_3+x)]
	log2n	Log 2 with zero threshold
	lognbz	Variable-base log with variable threshold
*	lognn	Variable-base log with zero threshold
	normp	Positive rectification
	npfnl	Non-parametric nonlinearity with boxcar filter (20 bins, 4 bin smoothing)
	npfnl0	Non-parametric nonlinearity with boxcar filter (20 bins, 4 bin smoothing, force zero minimum)
	npnl	Non-parametric nonlinearity (20 bins)
	npnl2	Non-parametric 2-dimensional nonlinearity (15 x 15 bins)
	npnl2b	Non-parametric 2-dimensional nonlinearity (10 x 10 bins)
	poly1	First-order polynomial (ie, linear rescaling)
	poly2	Second-order polynomial
	poly3	Third-order polynomial
	root2	Square root following zero threshold
	rootnb	Nth root following zero threshold plus variable offset
	rootnbz	Nth root following variable threshold plus variable offset
	sig	Error function sigmoid
	sigcauchy	Cauchy sigmoid
	sigell	Elliot sigmoid
	sigerf	Error function sigmoid
	siglog100	Logistic sigmoid
	sigrich	Richards sigmoid
	sigumbel	Gumbel sigmoid
	sigumber	Gumbel sigmoid, initial condition right shifted
	zexp	Decaying exponential
	zthresh	Variable hard threshold

Temporal filters (or spectro-temporal with no preceding spectral filter)

* ap<P>z<Z>	P-pole, Z-zero filter
ap<P>z<Z>nd	P-pole, Z-zero filter with fixed (15 ms) delay
doublewcg01fir15b	Two-channel Gaussian spectral filter with FIR temporal filter (not summed--used with "comb" static nonlinearities)
* fir<u>	FIR filter, U bins
fir<U>g	Gaussian temporal filter, sampled out to U bins
fir<U>gb	Skewed Gabor temporal filter (DeAngelis et al. 1999), sampled out to U bins
fir<U>m	Gamma function temporal filter, sampled out to U bins
* fir<U>p	Difference of exponential filter sampled out to U bins
lindeberg<A><B>min	Lindeberg kernel A spatial order, B temporal order
lindebergbest15minng	Best Lindeberg kernel
nsepfir<U>	Space-time separable linear filter, duration U
p<P>z<Z>x<N>	Bank of N P-pole, Z-zero filters

Spectral filters

linspec1	Lindeberg spectral filter, order 1
* wc<D>	Non-parametric D-channel spectral filter
* wcg<D>	D-channel Gaussian spectral filter
wcg<D>w	D-channel Gaussian spectral filter, fixed width (0.3 oct)
wcgd<D>	D-channel difference of Gaussians
wcgd<D>a	D-channel difference of Gaussians, narrow initial conditions
wcgr<D>	D-channel square root of Gaussian
wci<D>	D-channel Gaussian spectral filter
* wcm<D>	D-channel Morlet spectral filter
wcm<D>a	D-channel Morlet spectral filter, narrow initial conditions

**Fit algorithms**	("Iter" steps per iteration, "Growth" step size change)	**PreNL**	**Stop**	**Iter**	**Growth**
fit05c	Iterated coordinate descent, normalized MSE min stepsize stop criterion ("Stop")		10^-6	10	10%
fit05d	Iterated coordinate descent		10^-5.5	10	10%
fit05h	Iterated coordinate descent first without static nonlinearity ("PreNL")	10^-4	10^-5	10	10%
fit05i	Iterated coordinate descent first without static nonlinearity	10^-4	10^-4	10	10%
fit05j	Iterated coordinate descent first without static nonlinearity	10^-5	10^-4	10	10%
fit05k	Iterated coordinate descent first without static nonlinearity	10^-4.5	10^-4	10	10%
fit05m	Iterated coordinate descent first without static nonlinearity	10^-4	10^-5.5	10	10%
fit05t	Hybrid non-greedy(FIR)/greedy(no-FIR) coordinate descent first without static nonlinearity	10^-4	10^-6	10	10%
fit05t1	Hybrid coordinate descent first without static nonlinearity	10^-4	10^-6	10	30%
fit05t2	Hybrid coordinate descent first without static nonlinearity	10^-4	10^-6	10	100%
fit05t3	Hybrid coordinate descent first without static nonlinearity	10^-4	10^-6	5	30%
fit05t4	Hybrid coordinate descent first without static nonlinearity	10^-4	10^-6	5	200%
fit05u	Hybrid coordinate descent first without static nonlinearity	10^-4.5	10^-6	20	20%
fit05u1	Hybrid coordinate descent first without static nonlinearity	10^-4.5	10^-5	20	20%
fit05u2	Hybrid coordinate descent first without static nonlinearity	10^-4.5	10^-6	10	20%
fit05u3	Hybrid coordinate descent first without static nonlinearity	10^-4.5	10^-6	20	100%
fit05u4	Hybrid coordinate descent first without static nonlinearity	10^-4.5	10^-6	20	20%
fit05u5	Hybrid coordinate descent first without static nonlinearity	10^-4.5	10^-6	20	100%
fit05u6	Hybrid coordinate descent first without static nonlinearity	10^-4.5	10^-7	20	100%
fit05u7	Hybrid coordinate descent first without static nonlinearity	10^-4.5	10^-7	20	20%

	fit09ua	Hybrid coordinate descent first without static nonlinearity	10^-5	10^-6	20	100%
	fit05ub	Hybrid coordinate descent first without static nonlinearity	10^-5	10^-6	10	100%
*	fit05v	Hybrid coordinate descent first without static nonlinearity	10^-4	10^-6	10	10%
	fit05v1	Hybrid coordinate descent first without static nonlinearity	10^-4	10^-6.5	10	20%
	fit05v2	Hybrid coordinate descent first without static nonlinearity	10^-4	10^-6.5	20	20%
	fit05v3	Hybrid coordinate descent first without static nonlinearity	10^-4	10^-6.5	10	10%
	fit05v4	Hybrid coordinate descent first without static nonlinearity	10^-4	10^-6	10	10%
	fit05v5	Hybrid coordinate descent first without static nonlinearity	10^-4	10^-5.5	10	10%
	fit05v6	Hybrid coordinate descent first without static nonlinearity	10^-4	10^-6	10	50%
	fit05v7	Hybrid coordinate descent first without static nonlinearity	10^-4.5	10^-6	10	10%
	fit05v8	Hybrid coordinate descent first without static nonlinearity	10^-5	10^-6	10	20%
*	fit05w<X><Y>	Hybrid coordinate descent first without static nonlinearity	10^-4	10^-X.Y	10	10%
*	fit05x	Hybrid coordinate descent first without static nonlinearity, no shrinkage, L1-norm (Calabrese et al. 2011)	10^-4	10^-5	10	10%
	fit05x_<X><Y>	Hybrid coordinate descent first without static nonlinearity, no shrinkage, L1-norm (Calabrese et al. 2011)	10^-4	10^-X.Y	10	10%
*	fit05x0	Hybrid coordinate descent first without static nonlinearity, no shrinkage	10^-4	10^-5	10	10%
	fit05x0_<X><Y>	Hybrid coordinate descent first without static nonlinearity, no shrinkage	10^-4	10^-X.Y	10	10%
	fit05x2	Hybrid coordinate descent first without static nonlinearity, no shrinkage, weak L1-norm (Calabrese et al. 2011)	10^-4	10^-5	10	10%
	fit05x2_<X><Y>	Hybrid coordinate descent first without static nonlinearity, no shrinkage, weak L1-norm (Calabrese et al. 2011)	10^-4	10^-X.Y	10	10%
*	fit05x3	Hybrid coordinate descent first without static nonlinearity, L1-norm (Calabrese et al. 2011)	10^-4	10^-5	10	10%
	fit05x3_<X><Y>	Hybrid coordinate descent first without static nonlinearity, L1-norm (Calabrese et al. 2011)	10^-4	10^-X.Y	10	10%
	fit05x4	Hybrid coordinate descent first without static nonlinearity, weak L1-norm (Calabrese et al. 2011)	10^-4	10^-5	10	10%
	fit05x4_<X><Y>	Hybrid coordinate descent first without static nonlinearity, weak L1-norm (Calabrese et al. 2011)	10^-4	10^-X.Y	10	10%
	fit20b	Hybrid non-greedy(FIR)/greedy(no-FIR) coordinate descent, ML cost function		10^-7		
	fit21	Hybrid non-greedy(FIR)/greedy(no-FIR) coordinate descent, coherence cost function		10^-6		
	fitgen<N>	Preliminary genetic algortihm test				

Fit algorithm supplements

prefitrnd	Sample 10 random intial conditions, coordinate descent for 10 steps, 10% growth, choose best.
xvalnmse1	Early stopping by splitting estimation data in half.

#	Parm. count	Raw Fit R	Raw Test R	Corr. Test R	Model specification
1	10	0.209	0.300	0.361	fb18ch100_lognn_wcg01_ap1z0_dexp_fit05v
2	12	0.241	0.354	0.427	fb18ch100_log2_wcg01_ap3z1_dexp_fit05v
3	12	0.254	0.369	0.445	fb18ch100_lognn_wcg01_ap2z1_dexp_fit05v
4	13	0.251	0.367	0.443	fb18ch100_lognn_wcg01_ap2z1_siglog100_fit05v
5	13	0.254	0.370	0.445	fb18ch100_lognn_wcg01_ap3z1_dexp_fit05v
6	13	0.201	0.286	0.343	fb18ch100_lognn_wcg01_fir15gb_dexp_fit05v
7	13	0.260	0.366	0.443	fb18ch100_lognn_wcm01_ap2z1_dexp_fit05v
8	13	0.249	0.365	0.442	fb18ch100_rootn_wcg01_ap3z1_dexp_fit05v
9	14	0.250	0.366	0.441	fb18ch100_lognn_wcg01_ap3z1_siglog100_fit05v
10	14	0.255	0.369	0.445	fb18ch100_lognn_wcg01_ap3z2_dexp_fit05v
11	14	0.219	0.317	0.382	fb18ch100_lognn_wcg01_fir15p_dexp_fit05v
12	14	0.254	0.372	0.448	fb18ch100_lognn_wci01_ap3z1_dexp_fit05v
13	14	0.258	0.373	0.450	fb18ch100_lognn_wcm01_ap3z1_dexp_fit05v
14	15	0.266	0.377	0.453	fb18ch100_lognn_wcg02_ap1z0_dexp_fit05v
15	19	0.278	0.387	0.466	fb18ch100_lognn_wcg02_ap2z1_dexp_fit05v
16	20	0.268	0.383	0.462	fb18ch100_log2_wcg02_ap3z1_dexp_fit05v
17	20	0.260	0.375	0.455	fb18ch100_lognn_wcg01_fir12_dexp_fit05v
18	20	0.273	0.384	0.462	fb18ch100_lognn_wcg02_ap2z1_siglog100_fit05v
19	20	0.229	0.326	0.393	fb18ch100_lognn_wcg02_fir15gb_dexp_fit05v
20	20	0.275	0.382	0.460	fb18ch100_lognn_wcg03_ap1z0_dexp_fit05v
21	21	0.276	0.392	0.472	fb18ch100_lognn_wcg02_ap3z1_dexp_fit05v
22	21	0.279	0.390	0.470	fb18ch100_lognn_wcm02_ap2z1_dexp_fit05v
23	21	0.271	0.389	0.471	fb18ch100_rootn_wcg02_ap3z1_dexp_fit05v
24	22	0.250	0.358	0.432	fb18ch100_log2_wcg01_fir15_dexp_fit05v
25	22	0.273	0.384	0.462	fb18ch100_lognn_wcg02_ap3z1_siglog100_fit05v
26	22	0.259	0.376	0.453	fb18ch100_lognn_wcg02_fir15p_dexp_fit05v
27	23	0.262	0.378	0.459	fb18ch100_lognn_wcg01_fir15_dexp_fit05v
28	23	0.261	0.377	0.456	fb18ch100_lognn_wcg01_fir15_zsoft_fit05v
29	23	0.279	0.389	0.469	fb18ch100_lognn_wcg02_ap3z2_dexp_fit05v
30	23	0.276	0.391	0.471	fb18ch100_lognn_wci02_ap3z1_dexp_fit05v
31	23	0.278	0.386	0.466	fb18ch100_lognn_wcm02_ap3z1_dexp_fit05v
32	24	0.266	0.380	0.460	fb18ch100_lognn_wcm01_fir15_dexp_fit05v
33	25	0.244	0.341	0.413	fb18ch100_lognn_wcg03_ap3z1_fit05v
34	25	0.282	0.389	0.469	fb18ch100_lognn_wcg04_ap1z0_dexp_fit05v
35	26	0.286	0.392	0.473	fb18ch100_lognn_wcg03_ap2z1_dexp_fit05v
36	27	0.282	0.381	0.460	fb18ch100_lognn_wcg03_ap2z1_siglog100_fit05v
37	27	0.244	0.350	0.422	fb18ch100_lognn_wcg03_fir15gb_dexp_fit05v
38	28	0.277	0.385	0.464	fb18ch100_log2_wcg03_ap3z1_dexp_fit05v
39	28	0.264	0.377	0.457	fb18ch100_lognn_wcg01_fir20_dexp_fit05v
40	28	0.264	0.361	0.436	fb18ch100_normp_wcg03_ap3z1_dexp_fit05v
41	28	0.262	0.364	0.440	fb18ch100_wcg03_ap3z1_dexp_fit05v
42	29	0.279	0.393	0.475	fb12ch100_lognn_wcg03_ap3z1_dexp_fit05v
43	29	0.283	0.384	0.463	fb18ch100_dlog_wcg03_ap3z1_dexp_fit05v
44	29	0.286	0.402	0.485	fb18ch100_lognn_wcg03_ap3z1_dexp_fit05v
45	29	0.288	0.386	0.464	fb18ch100_lognn_wcm03_ap2z1_dexp_fit05v
46	29	0.284	0.390	0.470	fb18ch100_logn_wcg03_ap3z1_dexp_fit05v
47	29	0.284	0.390	0.469	fb18ch100_normp_dlog_wcg03_ap3z1_dexp_fit05v
48	29	0.286	0.401	0.483	fb18ch100_normp_lognn_wcg03_ap3z1_dexp_fit05v
49	29	0.286	0.401	0.483	fb18ch100_normp_logn_wcg03_ap3z1_dexp_fit05v
50	29	0.280	0.392	0.473	fb18ch100_normp_rootn_wcg03_ap3z1_dexp_fit05v
51	29	0.264	0.363	0.438	fb18ch100_normp_zthresh_wcg03_ap3z1_dexp_fit05v
52	29	0.281	0.397	0.478	fb18ch100_rootn_wcg03_ap3z1_dexp_fit05v
53	29	0.262	0.359	0.434	fb18ch100_zthresh_wcg03_ap3z1_dexp_fit05v

54	29	0.223	0.340	0.418	fb18ch200_lognn_wcg03_ap3z1_dexp_fit05v
55	29	0.167	0.276	0.333	fb18ch400_lognn_wcg03_ap3z1_dexp_fit05v
56	29	0.286	0.393	0.474	fb24ch100_lognn_wcg03_ap3z1_dexp_fit05v
57	29	0.285	0.403	0.487	fb36ch100_lognn_wcg03_ap3z1_dexp_fit05v
58	30	0.283	0.382	0.461	fb18ch100_dlogb_wcg03_ap3z1_dexp_fit05v
59	30	0.173	0.232	0.278	fb18ch100_exp_wcg03_ap3z1_dexp_fit05v
60	30	0.277	0.367	0.443	fb18ch100_invlin_wcg03_ap3z1_dexp_fit05v
61	30	0.285	0.392	0.472	fb18ch100_lognb_wcg03_ap3z1_dexp_fit05v
62	30	0.278	0.363	0.437	fb18ch100_lognn_wc01_ap3z1_dexp_fit05v
63	30	0.282	0.394	0.475	fb18ch100_lognn_wcg03_ap3z1_siglog100_fit05v
64	30	0.271	0.387	0.467	fb18ch100_lognn_wcg03_fir15p_dexp_fit05v
65	30	0.283	0.385	0.465	fb18ch100_lognn_wcg05_ap1z0_dexp_fit05v
66	30	0.286	0.392	0.472	fb18ch100_normp_dlogb_wcg03_ap3z1_dexp_fit05v
67	30	0.262	0.353	0.427	fb18ch100_normp_exp_wcg03_ap3z1_dexp_fit05v
68	30	0.281	0.381	0.460	fb18ch100_normp_invlin_wcg03_ap3z1_dexp_fit05v
69	30	0.286	0.399	0.481	fb18ch100_normp_lognb_wcg03_ap3z1_dexp_fit05v
70	30	0.265	0.358	0.432	fb18ch100_normp_poly1_wcg03_ap3z1_dexp_fit05v
71	30	0.280	0.390	0.471	fb18ch100_normp_rootnb_wcg03_ap3z1_dexp_fit05v
72	30	0.264	0.356	0.431	fb18ch100_poly1_wcg03_ap3z1_dexp_fit05v
73	30	0.281	0.394	0.476	fb18ch100_rootnb_wcg03_ap3z1_dexp_fit05v
74	31	0.286	0.393	0.473	fb18ch100_dlogbz_wcg03_ap3z1_dexp_fit05v
75	31	0.281	0.388	0.469	fb18ch100_invlinz_wcg03_ap3z1_dexp_fit05v
76	31	0.287	0.395	0.477	fb18ch100_lognbz_wcg03_ap3z1_dexp_fit05v
77	31	0.286	0.395	0.476	fb18ch100_normp_dlogbz_wcg03_ap3z1_dexp_fit05v
78	31	0.283	0.386	0.465	fb18ch100_normp_invlinz_wcg03_ap3z1_dexp_fit05v
79	31	0.287	0.392	0.473	fb18ch100_normp_lognbz_wcg03_ap3z1_dexp_fit05v
80	31	0.272	0.358	0.433	fb18ch100_normp_poly2_wcg03_ap3z1_dexp_fit05v
81	31	0.285	0.400	0.483	fb18ch100_normp_rootnbz_wcg03_ap3z1_dexp_fit05v
82	31	0.269	0.354	0.428	fb18ch100_poly2_wcg03_ap3z1_dexp_fit05v
83	31	0.285	0.396	0.476	fb18ch100_rootnbz_wcg03_ap3z1_dexp_fit05v
84	32	0.286	0.386	0.466	fb18ch100_dexp_wcg03_ap3z1_dexp_fit05v
85	32	0.289	0.389	0.469	fb18ch100_lognn_wcg03_ap3z2_dexp_fit05v
86	32	0.285	0.400	0.483	fb18ch100_lognn_wci03_ap3z1_dexp_fit05v
87	32	0.287	0.393	0.474	fb18ch100_lognn_wcm03_ap3z1_dexp_fit05v
88	32	0.288	0.392	0.473	fb18ch100_normp_dexp_wcg03_ap3z1_dexp_fit05v
89	32	0.276	0.362	0.437	fb18ch100_normp_poly3_wcg03_ap3z1_dexp_fit05v
90	32	0.286	0.381	0.460	fb18ch100_normp_sig_wcg03_ap3z1_dexp_fit05v
91	32	0.267	0.352	0.425	fb18ch100_poly3_wcg03_ap3z1_dexp_fit05v
92	32	0.259	0.352	0.426	fb18ch100_sig_wcg03_ap3z1_dexp_fit05v
93	33	0.289	0.389	0.469	fb18ch100_lognn_wcg04_ap2z1_dexp_fit05v
94	33	0.243	0.341	0.413	fb18ch100_lognn_wcg04_ap3z1_fit05v
95	33	0.245	0.329	0.396	fb18ch100_normp_sigcauchy_wcg03_ap3z1_dexp_fit05v
96	33	0.245	0.330	0.397	fb18ch100_normp_sigell_wcg03_ap3z1_dexp_fit05v
97	33	0.244	0.327	0.392	fb18ch100_normp_sigerf_wcg03_ap3z1_dexp_fit05v
98	33	0.284	0.385	0.466	fb18ch100_normp_siglog_wcg03_ap3z1_dexp_fit05v
99	33	0.289	0.391	0.471	fb18ch100_normp_sigrich_wcg03_ap3z1_dexp_fit05v
100	33	0.255	0.347	0.419	fb18ch100_normp_sigumbel_wcg03_ap3z1_dexp_fit05v
101	33	0.242	0.328	0.396	fb18ch100_normp_sigumber_wcg03_ap3z1_dexp_fit05v
102	33	0.251	0.345	0.418	fb18ch100_sigcauchy_wcg03_ap3z1_dexp_fit05v
103	33	0.246	0.333	0.402	fb18ch100_sigell_wcg03_ap3z1_dexp_fit05v
104	33	0.248	0.337	0.409	fb18ch100_sigerf_wcg03_ap3z1_dexp_fit05v
105	33	0.286	0.387	0.467	fb18ch100_siglog_wcg03_ap3z1_dexp_fit05v
106	33	0.285	0.385	0.465	fb18ch100_sigrich_wcg03_ap3z1_dexp_fit05v
107	33	0.252	0.343	0.416	fb18ch100_sigumbel_wcg03_ap3z1_dexp_fit05v
108	33	0.249	0.340	0.412	fb18ch100_sigumber_wcg03_ap3z1_dexp_fit05v

109	34	0.282	0.393	0.474	fb18ch100_lognn_wcg02_fir12_dexp_fit05v
110	34	0.285	0.384	0.463	fb18ch100_lognn_wcg04_ap2z1_siglog100_fit05v
111	34	0.254	0.362	0.437	fb18ch100_lognn_wcg04_fir15gb_dexp_fit05v
112	36	0.281	0.386	0.465	fb18ch100_log2_wcg04_ap3z1_dexp_fit05v
113	37	0.288	0.396	0.477	fb18ch100_lognn_wcg04_ap3z1_dexp_fit05v
114	37	0.288	0.381	0.461	fb18ch100_lognn_wcm04_ap2z1_dexp_fit05v
115	38	0.268	0.374	0.454	fb18ch100_lognn_wcg01_fir30_dexp_fit05v
116	38	0.285	0.392	0.473	fb18ch100_lognn_wcg04_ap3z1_siglog100_fit05v
117	38	0.275	0.387	0.466	fb18ch100_lognn_wcg04_fir15p_dexp_fit05v
118	39	0.269	0.374	0.452	fb18ch100_log2_wcg02_fir15_dexp_fit05v
119	40	0.284	0.379	0.458	fb18ch100_lognn_wc01_fir15_dexp_fit05v
120	40	0.286	0.394	0.476	fb18ch100_lognn_wcg02_fir15_dexp_fit05v
121	40	0.284	0.397	0.478	fb18ch100_lognn_wcg02_fir15_zsoft_fit05v
122	40	0.293	0.382	0.460	fb18ch100_lognn_wcg05_ap2z1_dexp_fit05v
123	41	0.294	0.382	0.460	fb18ch100_lognn_wcg04_ap3z2_dexp_fit05v
124	41	0.289	0.383	0.461	fb18ch100_lognn_wcg05_ap2z1_siglog100_fit05v
125	41	0.288	0.393	0.474	fb18ch100_lognn_wcm04_ap3z1_dexp_fit05v
126	42	0.284	0.390	0.470	fb18ch100_lognn_wcm02_fir15_dexp_fit05v
127	45	0.293	0.394	0.476	fb18ch100_lognn_wcg05_ap3z1_dexp_fit05v
128	46	0.262	0.347	0.420	fb18ch100_lin_wcg03_ap3z1_dexp_fit05v
129	46	0.288	0.398	0.479	fb18ch100_lognn_wcg05_ap3z1_siglog100_fit05v
130	46	0.280	0.392	0.472	fb18ch100_lognn_wcg05_fir15p_dexp_fit05v
131	46	0.253	0.321	0.387	fb18ch100_normp_lin_wcg03_ap3z1_dexp_fit05v
132	48	0.290	0.395	0.477	fb18ch100_lognn_wcg03_fir12_dexp_fit05v
133	50	0.288	0.391	0.472	fb18ch100_lognn_wcg02_fir20_dexp_fit05v
134	55	0.298	0.391	0.471	fb18ch100_lognn_wc02_ap3z1_dexp_fit05v
135	56	0.279	0.377	0.454	fb18ch100_log2_wcg03_fir15_dexp_fit05v
136	57	0.294	0.395	0.476	fb18ch100_lognn_wcg03_fir15_dexp_fit05v
137	57	0.293	0.393	0.474	fb18ch100_lognn_wcg03_fir15_zsoft_fit05v
138	60	0.296	0.397	0.479	fb18ch100_lognn_wcm03_fir15_dexp_fit05v
139	62	0.293	0.384	0.463	fb12ch100_lognn_wc02_fir15_dexp_fit05v
140	62	0.296	0.393	0.474	fb18ch100_lognn_wcg04_fir12_dexp_fit05v
141	70	0.296	0.386	0.466	fb18ch100_lognn_wcg02_fir30_dexp_fit05v
142	72	0.299	0.392	0.473	fb18ch100_lognn_wcg03_fir20_dexp_fit05v
143	74	0.305	0.385	0.464	fb18ch100_lognn_wc02_fir15_dexp_fit05v
144	74	0.301	0.389	0.469	fb18ch100_lognn_wcg04_fir15_dexp_fit05v
145	78	0.301	0.389	0.469	fb18ch100_lognn_wcm04_fir15_dexp_fit05v
146	80	0.307	0.384	0.462	fb18ch100_lognn_wc03_ap3z1_dexp_fit05v
147	86	0.310	0.376	0.453	fb24ch100_lognn_wc02_fir15_dexp_fit05v
148	91	0.307	0.384	0.463	fb18ch100_lognn_wcg05_fir15_dexp_fit05v
149	96	0.303	0.390	0.471	fb18ch100_lognn_wcm05_fir15_dexp_fit05v
150	104	0.241	0.321	0.396	fb18ch200_lognn_wc02_fir30_dexp_fit05v
151	105	0.308	0.387	0.465	fb18ch100_lognn_wc04_ap3z1_dexp_fit05v
152	108	0.316	0.377	0.454	fb18ch100_lognn_wc03_fir15_dexp_fit05v
153	110	0.318	0.364	0.438	fb36ch100_lognn_wc02_fir15_dexp_fit05v
154	142	0.323	0.364	0.438	fb18ch100_lognn_wc04_fir15_dexp_fit05v
155	144	0.182	0.261	0.315	fb18ch400_lognn_wc02_fir50_dexp_fit05v
156	176	0.328	0.365	0.440	fb18ch100_lognn_wc05_fir15_dexp_fit05v
157	186	0.324	0.351	0.423	fb12ch100_lognn_fir15_dexp_fit05v
158	272	0.305	0.355	0.427	fb18ch100_lognn_fir15_fit05v
159	276	0.354	0.336	0.406	fb18ch100_lognn_fir15_dexp_fit05v
160	366	0.375	0.308	0.371	fb24ch100_lognn_fir15_dexp_fit05v
161	546	0.299	0.261	0.325	fb18ch200_lognn_fir30_dexp_fit05v
162	546	0.408	0.283	0.342	fb36ch100_lognn_fir15_dexp_fit05v
163	906	0.232	0.211	0.258	fb18ch400_lognn_fir50_dexp_fit05v

164	3	0.137	0.188	0.229	fb18ch100_log2n_wcg01w_wc01_fit09c
165	5	0.173	0.249	0.301	fb18ch100_log2n_wcg01w_ap0z0_wc01_fit09c
166	6	0.186	0.265	0.321	fb18ch100_log2n_wcg01w_ap0z0_dexp3b_fit09c
167	6	0.163	0.228	0.275	fb18ch100_log2n_wcg01_ap1z0_wc01_fit09c
168	7	0.188	0.267	0.323	fb18ch100_log2n_wcg01w_ap0z0_dexp_fit09c
169	7	0.189	0.275	0.333	fb18ch100_log2n_wcg01w_ap1z0_dexp3b_fit09c
170	7	0.193	0.270	0.326	fb18ch100_log2n_wcg01_ap0z0_dexp3b_fit09c
171	7	0.192	0.274	0.330	fb18ch100_log2n_wcg01_ap1z0nd_dexp_fit09c
172	7	0.163	0.228	0.276	fb18ch100_log2n_wcg01_ap1z0_zthresh_wc01_fit09c
173	7	0.192	0.276	0.334	fb18ch100_lognn_wcg01w_ap0z0_dexp3b_fit09c
174	7	0.192	0.275	0.332	fb18ch100_lognzn_wcg01w_ap0z0_dexp3b_fit09c
175	8	0.191	0.270	0.327	fb18ch100_log2n_wcg01w_ap1z0_dexp_fit09c
176	8	0.219	0.321	0.386	fb18ch100_log2n_wcg01w_ap1z1_dexp3b_fit09c
177	8	0.190	0.278	0.335	fb18ch100_log2n_wcg01w_ap2z0_dexp3b_fit09c
178	8	0.196	0.273	0.330	fb18ch100_log2n_wcg01_ap0z0_dexp_fit09c
179	8	0.196	0.277	0.333	fb18ch100_log2n_wcg01_ap1z0_dexp3b_fit09c
180	8	0.202	0.276	0.334	fb18ch100_log2n_wcgd01_ap0z0_dexp3b_fit09c
181	8	0.194	0.276	0.334	fb18ch100_lognn_wcg01w_ap0z0_dexp_fit09c
182	8	0.196	0.284	0.342	fb18ch100_lognn_wcg01w_ap1z0_dexp3b_fit09c
183	8	0.201	0.283	0.342	fb18ch100_lognn_wcg01_ap0z0_dexp3b_fit09c
184	8	0.192	0.269	0.326	fb18ch100_lognzn_wcg01w_ap0z0_dexp_fit09c
185	8	0.198	0.280	0.338	fb18ch100_lognzn_wcg01w_ap1z0_dexp3b_fit09c
186	8	0.202	0.282	0.341	fb18ch100_lognzn_wcg01_ap0z0_dexp3b_fit09c
187	8	0.204	0.292	0.351	fb18ch100_normp_wcg01_ap1z0_dexp_fit09c
188	9	0.228	0.328	0.395	fb18ch100_log2n_wcg01w_ap1z1_dexp_fit09c
189	9	0.191	0.275	0.332	fb18ch100_log2n_wcg01w_ap2z0_dexp_fit09c
190	9	0.227	0.338	0.408	fb18ch100_log2n_wcg01w_ap2z1_dexp3b_fit09c
191	9	0.199	0.280	0.338	fb18ch100_log2n_wcg01_ap1z0_dexp_fit09c
192	9	0.231	0.332	0.399	fb18ch100_log2n_wcg01_ap1z1_dexp3b_fit09c
193	9	0.197	0.279	0.335	fb18ch100_log2n_wcg01_ap2z0_dexp3b_fit09c
194	9	0.233	0.341	0.409	fb18ch100_log2n_wcg01_ap2z1_dexp3a_fit09c
195	9	0.225	0.327	0.393	fb18ch100_log2n_wcg01_ap2z1_dexp3c_fit09c
196	9	0.206	0.283	0.342	fb18ch100_log2n_wcgd01_ap0z0_dexp_fit09c
197	9	0.206	0.284	0.343	fb18ch100_log2n_wcgd01_ap1z0_dexp3b_fit09c
198	9	0.198	0.287	0.346	fb18ch100_lognn_wcg01w_ap1z0_dexp_fit09c
199	9	0.224	0.324	0.390	fb18ch100_lognn_wcg01w_ap1z1_dexp3b_fit09c
200	9	0.196	0.287	0.347	fb18ch100_lognn_wcg01w_ap2z0_dexp3b_fit09c
201	9	0.203	0.286	0.346	fb18ch100_lognn_wcg01_ap0z0_dexp_fit09c
202	9	0.204	0.292	0.351	fb18ch100_lognn_wcg01_ap1z0_dexp3b_fit09c
203	9	0.209	0.290	0.351	fb18ch100_lognn_wcgd01_ap0z0_dexp3b_fit09c
204	9	0.199	0.280	0.339	fb18ch100_lognzn_wcg01w_ap1z0_dexp_fit09c
205	9	0.223	0.315	0.380	fb18ch100_lognzn_wcg01w_ap1z1_dexp3b_fit09c
206	9	0.206	0.285	0.344	fb18ch100_lognzn_wcg01_ap0z0_dexp_fit09c
207	9	0.206	0.287	0.347	fb18ch100_lognzn_wcg01_ap1z0_dexp3b_fit09c
208	9	0.236	0.343	0.414	fb18ch100_normp_wcg01_ap1z1_dexp_fit09c
209	10	0.235	0.343	0.414	fb18ch100_log2n_wcg01w_ap2z1_dexp_fit09c
210	10	0.226	0.336	0.404	fb18ch100_log2n_wcg01w_ap3z1_dexp3b_fit09c
211	10	0.239	0.338	0.406	fb18ch100_log2n_wcg01_ap1z1_dexp_fit09c
212	10	0.199	0.281	0.339	fb18ch100_log2n_wcg01_ap2z0_dexp_fit09c
213	10	0.236	0.345	0.415	fb18ch100_log2n_wcg01_ap2z1_dexp3b_fit09c
214	10	0.210	0.292	0.352	fb18ch100_log2n_wcgd01_ap1z0_dexp_fit09c
215	10	0.234	0.328	0.395	fb18ch100_log2n_wcgd01_ap1z1_dexp3b_fit09c
216	10	0.233	0.329	0.398	fb18ch100_lognn_wcg01w_ap1z1_dexp_fit09c
217	10	0.198	0.286	0.346	fb18ch100_lognn_wcg01w_ap2z0_dexp_fit09c
218	10	0.229	0.336	0.405	fb18ch100_lognn_wcg01w_ap2z1_dexp3b_fit09c

219	10	0.207	0.296	0.357	fb18ch100_lognn_wcg01_ap1z0_dexp_fit09c
220	10	0.238	0.339	0.408	fb18ch100_lognn_wcg01_ap1z1_dexp3b_fit09c
221	10	0.207	0.296	0.357	fb18ch100_lognn_wcg01_ap2z0_dexp3b_fit09c
222	10	0.215	0.296	0.358	fb18ch100_lognn_wcgd01_ap0z0_dexp_fit09c
223	10	0.213	0.296	0.358	fb18ch100_lognn_wcgd01_ap1z0_dexp3b_fit09c
224	10	0.231	0.331	0.399	fb18ch100_lognzn_wcg01w_ap1z1_dexp_fit09c
225	10	0.232	0.337	0.406	fb18ch100_lognzn_wcg01w_ap2z1_dexp3b_fit09c
226	10	0.209	0.292	0.352	fb18ch100_lognzn_wcg01_ap1z0_dexp_fit09c
227	10	0.236	0.339	0.409	fb18ch100_lognzn_wcg01_ap1z1_dexp3b_fit09c
228	10	0.209	0.284	0.343	fb18ch100_lognzn_wcgd01_ap0z0_dexp3b_fit09c
229	10	0.240	0.350	0.422	fb18ch100_normp_wcg01_ap2z1_dexp_fit09c
230	10	0.250	0.356	0.431	gt18ch100_logn_lindebergbest15minng_siglog100b_fitgen06
231	10	0.157	0.235	0.290	ozgf04x200_rootnb_wc01_p1z1_zthresh_fit09
232	11	0.234	0.341	0.411	fb18ch100_log2n_wcg01w_ap3z1_dexp_fit09c
233	11	0.245	0.356	0.429	fb18ch100_log2n_wcg01_ap2z1_dexp_fit09c
234	11	0.243	0.354	0.427	fb18ch100_log2n_wcg01_ap2z1_sigrich_fit09c
235	11	0.236	0.349	0.420	fb18ch100_log2n_wcg01_ap3z1_dexp3b_fit09c
236	11	0.246	0.339	0.409	fb18ch100_log2n_wcgd01_ap1z1_dexp_fit09c
237	11	0.240	0.345	0.417	fb18ch100_log2n_wcgd01_ap2z1_dexp3b_fit09c
238	11	0.216	0.309	0.371	fb18ch100_log2n_wcgd01_ap3z1_wc01_fit09c
239	11	0.243	0.357	0.430	fb18ch100_log2n_wcgr01_ap2z1_dexp_fit09c
240	11	0.239	0.348	0.420	fb18ch100_lognn_wcg01w_ap2z1_dexp_fit09c
241	11	0.231	0.344	0.414	fb18ch100_lognn_wcg01w_ap3z1_dexp3b_fit09c
242	11	0.248	0.348	0.420	fb18ch100_lognn_wcg01_ap1z1_dexp_fit09c
243	11	0.207	0.299	0.360	fb18ch100_lognn_wcg01_ap2z0_dexp_fit09c
244	11	0.242	0.351	0.423	fb18ch100_lognn_wcg01_ap2z1_dexp3b_fit09c
245	11	0.208	0.296	0.357	fb18ch100_lognn_wcg01_fir15g_dexp_fit09c
246	11	0.219	0.298	0.360	fb18ch100_lognn_wcgd01_ap1z0_dexp_fit09c
247	11	0.242	0.342	0.412	fb18ch100_lognn_wcgd01_ap1z1_dexp3b_fit09c
248	11	0.217	0.296	0.356	fb18ch100_lognn_wcm01_ap1z0_dexp_fit09c
249	11	0.241	0.350	0.422	fb18ch100_lognzn_wcg01w_ap2z1_dexp_fit09c
250	11	0.228	0.331	0.398	fb18ch100_lognzn_wcg01w_ap3z1_dexp3b_fit09c
251	11	0.244	0.342	0.413	fb18ch100_lognzn_wcg01_ap1z1_dexp_fit09c
252	11	0.243	0.346	0.417	fb18ch100_lognzn_wcg01_ap2z1_dexp3b_fit09c
253	11	0.213	0.289	0.348	fb18ch100_lognzn_wcgd01_ap0z0_dexp_fit09c
254	11	0.214	0.295	0.355	fb18ch100_lognzn_wcgd01_ap1z0_dexp3b_fit09c
255	11	0.241	0.354	0.427	fb18ch100_normp_wcg01_ap2z2_dexp_fit09c
256	11	0.236	0.348	0.420	fb18ch100_normp_wcg01_ap3z1_dexp_fit09c
257	11	0.167	0.233	0.283	gt18ch100_logn_lindeberg0015min_siglog100b_fit05c
258	11	0.184	0.255	0.307	gt18ch100_logn_lindeberg0015min_siglog100b_fit09c
259	11	0.213	0.294	0.355	gt18ch100_logn_lindeberg0015min_siglog100b_fitgen05
260	11	0.211	0.292	0.352	gt18ch100_logn_lindeberg0015min_siglog100b_fitgen06
261	11	0.249	0.359	0.435	gt18ch100_logn_lindeberg0115min_siglog100b_fitgen06
262	11	0.250	0.363	0.438	gt18ch100_logn_lindeberg0215min_siglog100b_fitgen06
263	11	0.232	0.317	0.383	gt18ch100_logn_lindeberg1015min_siglog100b_fitgen06
264	11	0.239	0.328	0.395	gt18ch100_logn_lindeberg1115min_siglog100b_fitgen06
265	11	0.246	0.349	0.422	gt18ch100_logn_lindeberg1215min_siglog100b_fitgen06
266	11	0.228	0.317	0.381	gt18ch100_logn_lindeberg2015min_siglog100b_fitgen06
267	11	0.240	0.343	0.414	gt18ch100_logn_lindeberg2115min_siglog100b_fitgen06
268	11	0.236	0.342	0.413	gt18ch100_logn_lindeberg2215min_siglog100b_fitgen06
269	12	0.244	0.356	0.429	fb18ch100_log2n_wcg01_ap3z1_dexp_fit09c
270	12	0.244	0.358	0.431	fb18ch100_log2n_wcg01_ap3z1_sigrich_fit09c
271	12	0.252	0.366	0.442	fb18ch100_log2n_wcgd01_ap2z1_dexp_fit09c
272	12	0.242	0.350	0.422	fb18ch100_log2n_wcgd01_ap3z1_dexp3b_fit09c
273	12	0.243	0.355	0.428	fb18ch100_log2n_wcgr01_ap2z1_sigrich_fit09c

274	12	0.245	0.359	0.433	fb18ch100_log2n_wcgr01_ap3z1_dexp_fit09c
275	12	0.252	0.365	0.440	fb18ch100_lognbn_wcg01_ap2z1_dexp_fit09c
276	12	0.240	0.350	0.421	fb18ch100_lognn_wcg01w_ap3z1_dexp_fit09c
277	12	0.253	0.365	0.439	fb18ch100_lognn_wcg01_ap2z1_dexp_fit09c
278	12	0.252	0.366	0.441	fb18ch100_lognn_wcg01_ap2z1_sigrich_fit09c
279	12	0.251	0.357	0.430	fb18ch100_lognn_wcg01_ap2z2_dexp_fit09c
280	12	0.244	0.358	0.430	fb18ch100_lognn_wcg01_ap3z1_dexp3b_fit09c
281	12	0.250	0.366	0.441	fb18ch100_lognn_wcg01_ap3z1_dexp_anneal_anneal_fit09c
282	12	0.252	0.369	0.444	fb18ch100_lognn_wcg01_ap3z1_dexp_anneal_fit09c
283	12	0.246	0.361	0.436	fb18ch100_lognn_wcg01_ap3z1_dexp_fit09g
284	12	0.252	0.369	0.444	fb18ch100_lognn_wcg01_ap3z1_dexp_fit09i
285	12	0.157	0.226	0.271	fb18ch100_lognn_wcg01_ap3z1_dexp_fit09j
286	12	0.147	0.209	0.253	fb18ch100_lognn_wcg01_ap3z1_dexp_fit09l
287	12	0.148	0.215	0.259	fb18ch100_lognn_wcg01_ap3z1_dexp_fit09m
288	12	0.205	0.296	0.356	fb18ch100_lognn_wcg01_fir15m_dexp_fit09c
289	12	0.250	0.349	0.421	fb18ch100_lognn_wcgd01_ap1z1_dexp_fit09c
290	12	0.246	0.347	0.419	fb18ch100_lognn_wcgd01_ap2z1_dexp3b_fit09c
291	12	0.251	0.364	0.439	fb18ch100_lognn_wcgr01_ap2z1_dexp_fit09c
292	12	0.209	0.297	0.357	fb18ch100_lognn_wcm01_fir15g_dexp_fit09c
293	12	0.241	0.347	0.419	fb18ch100_lognzn_wcg01w_ap3z1_dexp_fit09c
294	12	0.254	0.365	0.441	fb18ch100_lognzn_wcg01_ap2z1_dexp_fit09c
295	12	0.243	0.352	0.423	fb18ch100_lognzn_wcg01_ap3z1_dexp3b_fit09c
296	12	0.220	0.300	0.361	fb18ch100_lognzn_wcgd01_ap1z0_dexp_fit09c
297	12	0.242	0.336	0.406	fb18ch100_lognzn_wcgd01_ap1z1_dexp3b_fit09c
298	12	0.242	0.355	0.429	fb18ch100_normp_wcg01_ap3z2_dexp_fit09c
299	12	0.211	0.299	0.360	fb18ch100_normp_wcg02_ap1z0_dexp_fit09c
300	13	0.254	0.363	0.438	fb18ch100_log2n_wcgd01_ap2z1_sigrich_fit09c
301	13	0.251	0.363	0.438	fb18ch100_log2n_wcgd01_ap3z1_dexp_fit09c
302	13	0.243	0.356	0.429	fb18ch100_log2n_wcgr01_ap3z1_sigrich_fit09c
303	13	0.253	0.368	0.444	fb18ch100_lognbn_wcg01_ap3z1_dexp_fit09c
304	13	0.251	0.364	0.439	fb18ch100_lognbn_wcgr01_ap2z1_dexp_fit09c
305	13	0.255	0.365	0.441	fb18ch100_lognbzn_wcg01_ap2z1_dexp_fit09c
306	13	0.252	0.368	0.444	fb18ch100_lognn_wcg01_ap3z1_dexp_fit05vb
307	13	0.252	0.367	0.443	fb18ch100_lognn_wcg01_ap3z1_dexp_fit05vc
308	13	0.253	0.366	0.442	fb18ch100_lognn_wcg01_ap3z1_dexp_fit09c
309	13	0.241	0.354	0.426	fb18ch100_lognn_wcg01_ap3z1_dexp_fit20a
310	13	0.116	0.167	0.201	fb18ch100_lognn_wcg01_ap3z1_dexp_fit21
311	13	0.252	0.370	0.446	fb18ch100_lognn_wcg01_ap3z1_sigrich_fit09c
312	13	0.251	0.359	0.433	fb18ch100_lognn_wcg01_ap3z2_dexp_fit09c
313	13	0.250	0.366	0.440	fb18ch100_lognn_wcg01_ap4z1_dexp_fit09c
314	13	0.259	0.369	0.445	fb18ch100_lognn_wcgd01_ap2z1_dexp_fit09c
315	13	0.247	0.356	0.431	fb18ch100_lognn_wcgd01_ap3z1_dexp3b_fit09c
316	13	0.252	0.368	0.444	fb18ch100_lognn_wcgr01_ap2z1_sigrich_fit09c
317	13	0.203	0.288	0.346	fb18ch100_lognn_wcm01_fir15m_dexp_fit09c
318	13	0.253	0.367	0.442	fb18ch100_lognzn_wcg01_ap3z1_dexp_fit09c
319	13	0.249	0.345	0.416	fb18ch100_lognzn_wcgd01_ap1z1_dexp_fit09c
320	13	0.247	0.349	0.422	fb18ch100_lognzn_wcgd01_ap2z1_dexp3b_fit09c
321	14	0.249	0.360	0.434	fb18ch100_log2n_wcgd01_ap3z1_sigrich_fit09c
322	14	0.259	0.369	0.445	fb18ch100_lognbn_wcgd01_ap2z1_dexp_fit09c
323	14	0.252	0.366	0.441	fb18ch100_lognbn_wcgr01_ap3z1_dexp_fit09c
324	14	0.253	0.367	0.442	fb18ch100_lognbzn_wcg01_ap3z1_dexp_fit09c
325	14	0.254	0.370	0.446	fb18ch100_lognn_wcg01_ap4z2_dexp_fit09c
326	14	0.216	0.310	0.373	fb18ch100_lognn_wcg01_fir15p_dexp_fit09c
327	14	0.257	0.365	0.440	fb18ch100_lognn_wcgd01_ap2z1_sigrich_fit09c
328	14	0.253	0.367	0.442	fb18ch100_lognn_wcgr01_ap3z1_dexp_fit09c

329	14	0.251	0.368	0.443	fb18ch100_lognn_wcgr01_ap3z1_sigrich_fit09c
330	14	0.256	0.355	0.428	fb18ch100_lognn_wcm01_ap3z1_dexp_fit05h_fit19g
331	14	0.257	0.358	0.433	fb18ch100_lognn_wcm01_ap3z1_dexp_fit09c
332	14	0.260	0.364	0.439	fb18ch100_lognzn_wcgd01_ap2z1_dexp_fit09c
333	14	0.248	0.354	0.427	fb18ch100_lognzn_wcgd01_ap3z1_dexp3b_fit09c
334	14	0.247	0.359	0.433	fb18ch100_normp_wcg02_ap1z1_dexp_fit09c
335	14	0.162	0.229	0.277	gt12ch100_xvalnmse1_logfree_lindeberg00_20_siglog100_fit05
336	14	0.184	0.258	0.311	gt12ch100_xvalnmse1_logfree_lindeberg00_20_siglog100_fit15rnd
337	14	0.139	0.199	0.241	gt12ch100_xvalnmse1_logfree_lindeberg01_20_siglog100_fit05
338	14	0.200	0.280	0.338	gt12ch100_xvalnmse1_logfree_lindeberg01_20_siglog100_fit15rnd
339	14	0.134	0.176	0.210	gt12ch100_xvalnmse1_logfree_lindeberg02_20_siglog100_fit05
340	14	0.200	0.282	0.341	gt12ch100_xvalnmse1_logfree_lindeberg02_20_siglog100_fit15rnd
341	14	0.113	0.151	0.180	gt12ch100_xvalnmse1_logfree_lindeberg10_20_siglog100_fit05
342	14	0.179	0.246	0.297	gt12ch100_xvalnmse1_logfree_lindeberg10_20_siglog100_fit15rnd
343	14	0.084	0.091	0.109	gt12ch100_xvalnmse1_logfree_lindeberg11_20_siglog100_fit05
344	14	0.187	0.254	0.306	gt12ch100_xvalnmse1_logfree_lindeberg11_20_siglog100_fit15rnd
345	14	0.098	0.122	0.146	gt12ch100_xvalnmse1_logfree_lindeberg12_20_siglog100_fit05
346	14	0.193	0.258	0.312	gt12ch100_xvalnmse1_logfree_lindeberg12_20_siglog100_fit15rnd
347	14	0.079	0.135	0.161	gt12ch100_xvalnmse1_logfree_lindeberg20_20_siglog100_fit05
348	14	0.198	0.273	0.329	gt12ch100_xvalnmse1_logfree_lindeberg20_20_siglog100_fit15rnd
349	14	0.082	0.141	0.169	gt12ch100_xvalnmse1_logfree_lindeberg21_20_siglog100_fit05
350	14	0.198	0.273	0.328	gt12ch100_xvalnmse1_logfree_lindeberg21_20_siglog100_fit15rnd
351	14	0.150	0.205	0.247	gt12ch100_xvalnmse1_logfree_lindeberg22_20_siglog100_fit05
352	14	0.198	0.273	0.329	gt12ch100_xvalnmse1_logfree_lindeberg22_20_siglog100_fit15rnd
353	14	0.157	0.230	0.277	gt12ch100_xvalnmse2_logfree_lindeberg00_20_siglog100_fit05
354	14	0.179	0.262	0.315	gt12ch100_xvalnmse2_logfree_lindeberg00_20_siglog100_fit15rnd
355	14	0.153	0.234	0.282	gt12ch100_xvalnmse2_logfree_lindeberg01_20_siglog100_fit05
356	14	0.199	0.289	0.348	gt12ch100_xvalnmse2_logfree_lindeberg01_20_siglog100_fit15rnd
357	14	0.149	0.219	0.263	gt12ch100_xvalnmse2_logfree_lindeberg02_20_siglog100_fit05
358	14	0.194	0.284	0.342	gt12ch100_xvalnmse2_logfree_lindeberg02_20_siglog100_fit15rnd
359	14	0.120	0.179	0.214	gt12ch100_xvalnmse2_logfree_lindeberg10_20_siglog100_fit05
360	14	0.182	0.256	0.307	gt12ch100_xvalnmse2_logfree_lindeberg10_20_siglog100_fit15rnd
361	14	0.089	0.117	0.140	gt12ch100_xvalnmse2_logfree_lindeberg11_20_siglog100_fit05
362	14	0.184	0.255	0.307	gt12ch100_xvalnmse2_logfree_lindeberg11_20_siglog100_fit15rnd
363	14	0.061	0.071	0.083	gt12ch100_xvalnmse2_logfree_lindeberg12_20_siglog100_fit05
364	14	0.187	0.256	0.307	gt12ch100_xvalnmse2_logfree_lindeberg12_20_siglog100_fit15rnd
365	14	0.083	0.119	0.142	gt12ch100_xvalnmse2_logfree_lindeberg20_20_siglog100_fit05
366	14	0.197	0.284	0.342	gt12ch100_xvalnmse2_logfree_lindeberg20_20_siglog100_fit15rnd
367	14	0.098	0.158	0.189	gt12ch100_xvalnmse2_logfree_lindeberg21_20_siglog100_fit05
368	14	0.195	0.275	0.331	gt12ch100_xvalnmse2_logfree_lindeberg21_20_siglog100_fit15rnd
369	14	0.152	0.224	0.269	gt12ch100_xvalnmse2_logfree_lindeberg22_20_siglog100_fit05
370	14	0.192	0.268	0.322	gt12ch100_xvalnmse2_logfree_lindeberg22_20_siglog100_fit15rnd
371	14	0.191	0.287	0.345	gt18ch100_linspec0_p1z1_siglog100_fit05
372	14	0.225	0.328	0.396	gt18ch100_linspec0_p1z1_siglog100_fit15rnd
373	14	0.179	0.260	0.313	gt18ch100_linspec1_p1z1_siglog100_fit05
374	14	0.221	0.320	0.386	gt18ch100_linspec1_p1z1_siglog100_fit15rnd
375	14	0.152	0.197	0.236	gt18ch100_linspec2_p1z1_siglog100_fit05
376	14	0.222	0.318	0.383	gt18ch100_linspec2_p1z1_siglog100_fit15rnd
377	14	0.181	0.262	0.327	gt18ch100_logfree_lindeberg00_20_siglog100_fit05
378	14	0.210	0.304	0.366	gt18ch100_logfree_lindeberg00_20_siglog100_fit15rnd
379	14	0.194	0.287	0.357	gt18ch100_logfree_lindeberg01_20_siglog100_fit05
380	14	0.225	0.327	0.394	gt18ch100_logfree_lindeberg01_20_siglog100_fit15rnd
381	14	0.105	0.148	0.180	gt18ch100_logfree_lindeberg02_20_siglog100_fit05
382	14	0.218	0.314	0.379	gt18ch100_logfree_lindeberg02_20_siglog100_fit15rnd
383	14	0.081	0.111	0.135	gt18ch100_logfree_lindeberg10_20_siglog100_fit05

384	14	0.216	0.299	0.361	gt18ch100_logfree_lindeberg10_20_siglog100_fit15rnd
385	14	0.064	0.079	0.092	gt18ch100_logfree_lindeberg11_20_siglog100_fit05
386	14	0.214	0.300	0.362	gt18ch100_logfree_lindeberg11_20_siglog100_fit15rnd
387	14	0.028	0.048	0.056	gt18ch100_logfree_lindeberg12_20_siglog100_fit05
388	14	0.213	0.298	0.359	gt18ch100_logfree_lindeberg12_20_siglog100_fit15rnd
389	14	0.055	0.108	0.134	gt18ch100_logfree_lindeberg20_20_siglog100_fit05
390	14	0.210	0.296	0.357	gt18ch100_logfree_lindeberg20_20_siglog100_fit15rnd
391	14	0.063	0.108	0.133	gt18ch100_logfree_lindeberg21_20_siglog100_fit05
392	14	0.216	0.296	0.356	gt18ch100_logfree_lindeberg21_20_siglog100_fit15rnd
393	14	0.160	0.229	0.284	gt18ch100_logfree_lindeberg22_20_siglog100_fit05
394	14	0.214	0.300	0.359	gt18ch100_logfree_lindeberg22_20_siglog100_fit15rnd
395	14	0.223	0.313	0.377	gt18ch100_logfree_lindeberg_siglog100_bestorder
396	14	0.198	0.295	0.354	gt18ch100_logn_lindeberg0015_siglog100b_fit05c
397	14	0.200	0.295	0.354	gt18ch100_logn_lindeberg0015_siglog100b_fit05g_fit05c
398	14	0.202	0.285	0.346	gt18ch100_logn_lindeberg0015_siglog100b_fitgen02
399	14	0.203	0.301	0.362	gt18ch100_logn_lindeberg0115_siglog100b_fit05c
400	14	0.206	0.301	0.363	gt18ch100_logn_lindeberg0115_siglog100b_fit05g_fit05c
401	14	0.197	0.293	0.353	gt18ch100_logn_lindeberg0215_siglog100b_fit05c
402	14	0.201	0.290	0.349	gt18ch100_logn_lindeberg0215_siglog100b_fit05g_fit05c
403	14	0.186	0.259	0.310	gt18ch100_logn_lindeberg0215_siglog100b_fitgen02
404	14	0.178	0.253	0.303	gt18ch100_logn_lindeberg1015_siglog100b_fit05c
405	14	0.178	0.251	0.301	gt18ch100_logn_lindeberg1015_siglog100b_fit05g_fit05c
406	14	0.188	0.254	0.305	gt18ch100_logn_lindeberg1115_siglog100b_fit05c
407	14	0.186	0.252	0.303	gt18ch100_logn_lindeberg1115_siglog100b_fit05g_fit05c
408	14	0.161	0.215	0.260	gt18ch100_logn_lindeberg1115_siglog100b_fitgen02
409	14	0.178	0.251	0.303	gt18ch100_logn_lindeberg1215_siglog100b_fit05c
410	14	0.181	0.251	0.302	gt18ch100_logn_lindeberg1215_siglog100b_fit05g_fit05c
411	14	0.162	0.220	0.265	gt18ch100_logn_lindeberg1215_siglog100b_fitgen02
412	14	0.151	0.188	0.223	gt18ch100_logn_lindeberg2015_siglog100b_fit05c
413	14	0.154	0.191	0.226	gt18ch100_logn_lindeberg2015_siglog100b_fit05g_fit05c
414	14	0.174	0.234	0.282	gt18ch100_logn_lindeberg2015_siglog100b_fitgen02
415	14	0.149	0.193	0.233	gt18ch100_logn_lindeberg2115_siglog100b_fit05c
416	14	0.149	0.198	0.238	gt18ch100_logn_lindeberg2115_siglog100b_fit05g_fit05c
417	14	0.158	0.207	0.248	gt18ch100_logn_lindeberg2115_siglog100b_fitgen02
418	14	0.137	0.184	0.220	gt18ch100_logn_lindeberg2215_siglog100b_fit05c
419	14	0.139	0.185	0.220	gt18ch100_logn_lindeberg2215_siglog100b_fit05g_fit05c
420	14	0.167	0.221	0.267	gt18ch100_logn_lindeberg2215_siglog100b_fitgen02
421	14	0.152	0.234	0.289	gt36ch200_logfree_lindeberg00_40_siglog_fit15rnd
422	14	0.169	0.250	0.309	ozgf04x200_lognbz_wc01_p1z1_dexp_fit09
423	15	0.259	0.370	0.447	fb18ch100_lognbn_wcgd01_ap3z1_dexp_fit09c
424	15	0.231	0.327	0.394	fb18ch100_lognn_wcg01_ap1z0x3_dexp_fit09c
425	15	0.229	0.322	0.387	fb18ch100_lognn_wcg01_fir15p_siglog100_fit05h_fit19g
426	15	0.259	0.361	0.435	fb18ch100_lognn_wcg02_ap1z0_dexp_fit09c
427	15	0.259	0.362	0.435	fb18ch100_lognn_wcg02_ap1z1_dexp_fit09c
428	15	0.260	0.370	0.446	fb18ch100_lognn_wcgd01_ap3z1_dexp_fit09c
429	15	0.258	0.371	0.447	fb18ch100_lognn_wcgd01_ap3z1_sigrich_fit09c
430	15	0.232	0.323	0.390	fb18ch100_lognn_wcm01_fir15p_dexp_fit05h_fit19g
431	15	0.216	0.307	0.370	fb18ch100_lognn_wcm01_fir15p_dexp_fit09c
432	15	0.259	0.373	0.450	fb18ch100_lognzn_wcgd01_ap3z1_dexp_fit09c
433	16	0.257	0.369	0.444	fb18ch100_log2n_wcg02_ap2z1_dexp_fit09c
434	16	0.252	0.364	0.440	fb18ch100_lognn_wcg02_fir15g_dexp_fit09c
435	16	0.249	0.359	0.433	fb18ch100_normp_wcg02_ap2z1_dexp_fit09c
436	17	0.256	0.367	0.441	fb18ch100_log2n_wcg02_ap2z1_sigrich_fit09c
437	17	0.260	0.373	0.450	fb18ch100_lognn_wcg02_ap2z0_dexp_fit09c
438	17	0.241	0.324	0.389	fb18ch100_lognn_wcm02_ap1z0_dexp_fit09c

439	18	0.256	0.364	0.438	fb18ch100_log2n_wcg02_ap3z1_dexp_fit09c
440	18	0.262	0.378	0.454	fb18ch100_lognbn_wcg02_ap2z1_dexp_fit09c
441	18	0.264	0.378	0.454	fb18ch100_lognn_wcg02_ap2z1_sigrich_fit09c
442	18	0.244	0.349	0.421	fb18ch100_lognn_wcg02_fir15m_dexp_fit09c
443	18	0.242	0.337	0.405	fb18ch100_lognn_wcm02_fir15g_dexp_fit09c
444	18	0.252	0.357	0.430	fb18ch100_normp_wcg02_ap2z2_dexp_fit09c
445	18	0.245	0.359	0.433	fb18ch100_normp_wcg02_ap3z1_dexp_fit09c
446	19	0.112	0.148	0.181	fb18ch100_log2n_wc01
447	19	0.175	0.238	0.289	fb18ch100_log2n_wc01_fit09c
448	19	0.256	0.365	0.438	fb18ch100_log2n_wcg02_ap3z1_sigrich_fit09c
449	19	0.265	0.375	0.451	fb18ch100_lognbzn_wcg02_ap2z1_dexp_fit09c
450	19	0.237	0.342	0.412	fb18ch100_lognn_wcg01_fir15_fit05c
451	19	0.258	0.364	0.439	fb18ch100_lognn_wcg01_fir15_npnl_fit05c
452	19	0.274	0.383	0.462	fb18ch100_lognn_wcg02_ap2z1_dexp_fit09c
453	19	0.265	0.376	0.452	fb18ch100_lognn_wcg02_ap2z2_dexp_fit09c
454	19	0.112	0.149	0.181	gt18ch100_log2n_wc01
455	20	0.263	0.378	0.455	fb18ch100_lognbn_wcg02_ap3z1_dexp_fit09c
456	20	0.243	0.345	0.416	fb18ch100_lognn_wcg02_ap3z1_siglog100_fit09c
457	20	0.259	0.376	0.451	fb18ch100_lognn_wcg02_ap3z1_sigrich_fit09c
458	20	0.274	0.381	0.460	fb18ch100_lognn_wcg03_ap1z0_dexp_fit09c
459	20	0.237	0.327	0.393	fb18ch100_lognn_wcm02_fir15m_dexp_fit09c
460	20	0.263	0.373	0.449	fb18ch100_lognzn_wcg02_ap3z1_dexp_fit09c
461	20	0.255	0.363	0.438	fb18ch100_normp_wcg02_ap3z2_dexp_fit09c
462	20	0.149	0.198	0.245	ozgf01fb12x200_dlog_wc01_p1z1_zthresh_fit10
463	20	0.176	0.260	0.362	ozgf01fb12x200_logn_wc01_p1z1_zthresh_fit10
464	20	0.178	0.266	0.360	ozgf01fb12x200_rootn_wc01_p1z1_zthresh_fit10
465	21	0.156	0.241	0.334	apgt04x200_npfnl0_fir20_fit10
466	21	0.129	0.199	0.274	apgt04x200_npfnl_fir20_fit10
467	21	0.164	0.251	0.349	apgt04x200_npnl_fir20_fit10
468	21	0.263	0.372	0.447	fb18ch100_lognbzn_wcg02_ap3z1_dexp_fit09c
469	21	0.276	0.387	0.466	fb18ch100_lognn_wcg02_ap3z1_dexp_fit05vb
470	21	0.276	0.390	0.470	fb18ch100_lognn_wcg02_ap3z1_dexp_fit05vc
471	21	0.275	0.386	0.466	fb18ch100_lognn_wcg02_ap3z1_dexp_fit09c
472	21	0.259	0.372	0.448	fb18ch100_lognn_wcg02_ap3z1_dexp_fit20a
473	21	0.113	0.157	0.194	fb18ch100_lognn_wcg02_ap3z1_dexp_fit21
474	21	0.276	0.392	0.472	fb18ch100_lognn_wcg02_ap3z1_dexp_fit23
475	21	0.275	0.388	0.469	fb18ch100_lognn_wcg02_ap3z1_dexp_fit23a
476	21	0.275	0.389	0.469	fb18ch100_lognn_wcg02_ap3z1_dexp_fit23b
477	21	0.273	0.389	0.469	fb18ch100_lognn_wcg02_ap3z1_dexp_fit23c
478	21	0.274	0.389	0.469	fb18ch100_lognn_wcg02_ap3z1_dexp_fit23d
479	21	0.260	0.373	0.450	fb18ch100_lognn_wcg03_fir15g_dexp_fit09c
480	21	0.148	0.198	0.245	ozgf01fb12x200_dlogb_wc01_p1z1_zthresh_fit10
481	21	0.169	0.251	0.311	ozgf01fb12x200_invlin_wc01_p1z1_zthresh_fit10
482	21	0.176	0.259	0.359	ozgf01fb12x200_lognb_wc01_p1z1_zthresh_fit10
483	21	0.178	0.267	0.372	ozgf01fb12x200_rootnb_wc01_p1z1_zthresh_fit10
484	22	0.163	0.251	0.350	apgt04x200_dlog_fir20_fit10
485	22	0.149	0.236	0.327	apgt04x200_lin_fir20_fit10
486	22	0.163	0.251	0.349	apgt04x200_logn_fir20_fit10
487	22	0.154	0.229	0.318	apgt04x200_rootn_fir20_fit10
488	22	0.149	0.235	0.325	apgt04x200_zthresh_fir20_fit10
489	22	0.256	0.371	0.447	fb18ch100_lognn_wcg02_fir15p_dexp_fit09c
490	22	0.149	0.192	0.238	ozgf01fb12x200_dlogbz_wc01_p1z1_zthresh_fit10
491	22	0.168	0.254	0.316	ozgf01fb12x200_invlinz_wc01_p1z1_zthresh_fit10
492	22	0.182	0.271	0.366	ozgf01fb12x200_lognbz_wc01_p1z1_zthresh_fit10
493	22	0.180	0.273	0.337	ozgf01fb12x200_rootnbz_wc01_p1z1_zthresh_fit10

494	23	0.164	0.252	0.350	apgt04x200_dlogb_fir20_fit10
495	23	0.139	0.212	0.292	apgt04x200_exp_fir20_fit10
496	23	0.157	0.242	0.337	apgt04x200_invlin_fir20_fit10
497	23	0.147	0.234	0.324	apgt04x200_poly1_fir20_fit10
498	23	0.153	0.228	0.315	apgt04x200_rootnb_fir20_fit10
499	23	0.263	0.376	0.456	fb18ch100_lognn_wcg01_fir15_dexp_fit05vb
500	23	0.255	0.375	0.450	fb18ch100_lognn_wcg02_fir15p_siglog100_fit05h_fit05c
501	23	0.273	0.388	0.468	fb18ch100_lognn_wcg02_fir15p_siglog100_fit05h_fit19g
502	23	0.257	0.383	0.461	fb18ch100_lognn_wcg02_fir30p_siglog100_fit05h
503	23	0.275	0.284	0.344	fb18ch100_lognn_wcg03_ap1z1_dexp_fit09c
504	23	0.274	0.386	0.465	fb18ch100_lognn_wcg03_ap2z0_dexp_fit09c
505	23	0.274	0.387	0.467	fb18ch100_lognn_wcgr02_ap3z1_dexp_fit09c
506	23	0.277	0.371	0.449	fb18ch100_lognn_wcm02_ap3z1_dexp_fit05h_fit19g
507	23	0.273	0.372	0.450	fb18ch100_lognn_wcm02_ap3z1_dexp_fit09c
508	23	0.254	0.336	0.404	fb18ch100_lognn_wcm03_ap1z0_dexp_fit09c
509	23	0.236	0.327	0.394	gt12ch100_wc01_p1z1_siglog100_fit10
510	23	0.168	0.238	0.294	ozgf01fb12x200_dlog_wc01_p1z1_dexp_fit10
511	23	0.180	0.259	0.361	ozgf01fb12x200_logn_wc01_p1z1_dexp_fit10
512	23	0.143	0.206	0.282	ozgf01fb12x200_logn_wc01_p1z1_sig_fit10
513	23	0.183	0.264	0.357	ozgf01fb12x200_rootn_wc01_p1z1_dexp_fit10
514	24	0.169	0.257	0.358	apgt04x200_dlogbz_fir20_fit10
515	24	0.164	0.244	0.339	apgt04x200_invlinz_fir20_fit10
516	24	0.156	0.241	0.335	apgt04x200_poly2_fir20_fit10
517	24	0.166	0.248	0.344	apgt04x200_rootnbz_fir20_fit10
518	24	0.260	0.381	0.460	fb18ch100_lognn_wcg01_fir15_siglog100_fit05h_fit05c
519	24	0.256	0.366	0.442	fb18ch100_lognn_wcg03_fir15m_dexp_fit09c
520	24	0.266	0.381	0.462	fb18ch100_lognn_wcm01_fir15_dexp_fit05vb
521	24	0.268	0.367	0.443	fb18ch100_lognn_wcm02_fir15p_dexp_fit05h_fit19g
522	24	0.243	0.339	0.411	fb18ch100_lognn_wcm02_fir15p_dexp_fit09c
523	24	0.250	0.351	0.423	fb18ch100_lognn_wcm03_fir15g_dexp_fit09c
524	24	0.195	0.294	0.362	gt12ch200_logn_wc01_p1z1_siglog_fit10
525	24	0.167	0.236	0.291	ozgf01fb12x200_dlogb_wc01_p1z1_dexp_fit10
526	24	0.168	0.236	0.291	ozgf01fb12x200_dlog_wc01_p1z1_siglog_fit10
527	24	0.159	0.221	0.272	ozgf01fb12x200_dlog_wc01_p1z1_sigrich_fit10
528	24	0.172	0.256	0.316	ozgf01fb12x200_invlin_wc01_p1z1_dexp_fit10
529	24	0.180	0.257	0.357	ozgf01fb12x200_lognb_wc01_p1z1_dexp_fit10
530	24	0.143	0.208	0.284	ozgf01fb12x200_lognb_wc01_p1z1_sig_fit10
531	24	0.153	0.213	0.292	ozgf01fb12x200_logn_wc01_p1z1_sigcauchy_fit10
532	24	0.151	0.210	0.291	ozgf01fb12x200_logn_wc01_p1z1_sigell_fit10
533	24	0.162	0.233	0.322	ozgf01fb12x200_logn_wc01_p1z1_sigerf_fit10
534	24	0.179	0.258	0.360	ozgf01fb12x200_logn_wc01_p1z1_siglog_fit10
535	24	0.175	0.250	0.350	ozgf01fb12x200_logn_wc01_p1z1_sigrich_fit10
536	24	0.158	0.230	0.317	ozgf01fb12x200_logn_wc01_p1z1_sigumbel_fit10
537	24	0.104	0.149	0.210	ozgf01fb12x200_logn_wc01_p1z1_sigumber_fit10
538	24	0.183	0.264	0.368	ozgf01fb12x200_rootnb_wc01_p1z1_dexp_fit10
539	24	0.148	0.216	0.297	ozgf01fb12x200_rootnb_wc01_p1z1_sig_fit10
540	24	0.162	0.231	0.321	ozgf01fb12x200_rootn_wc01_p1z1_sigcauchy_fit10
541	24	0.179	0.266	0.370	ozgf01fb12x200_rootn_wc01_p1z1_siglog_fit10
542	24	0.181	0.268	0.350	ozgf01fb12x200_rootn_wc01_p1z1_sigrich_fit10
543	24	0.117	0.162	0.228	ozgf01fb12x200_rootn_wc01_p1z1_sigumber_fit10
544	24	0.128	0.180	0.223	ozgf04fb12x200_lognbz_wc01_p1z0_dexp_fit09
545	25	0.169	0.251	0.349	apgt04x200_dexp_fir20_fit10
546	25	0.159	0.243	0.338	apgt04x200_poly3_fir20_fit10
547	25	0.168	0.250	0.348	apgt04x200_sig_fir20_fit10
548	25	0.070	0.231	0.319	apgt04x200_zsoft_fir20_fit10

549	25	0.243	0.337	0.409	fb18ch100_lognn_wcg03_ap3z1_fit09c
550	25	0.255	0.361	0.436	fb18ch100_lognn_wcg03_ap3z1_npfnl0_fit09c
551	25	0.250	0.355	0.428	fb18ch100_lognn_wcg03_ap3z1_npfnl_fit09c
552	25	0.239	0.336	0.404	fb18ch100_lognn_wcg03_ap3z1_npnl_fit09c
553	25	0.279	0.387	0.467	fb18ch100_lognn_wcg04_ap1z0_dexp_fit09c
554	25	0.277	0.385	0.464	fb18ch100_lognn_wcgd02_ap3z1_dexp_fit09c
555	25	0.259	0.378	0.456	fb18ch100_lognn_wcgr01_fir15_siglog100_fit05h_fit05c
556	25	0.264	0.381	0.460	fb18ch100_lognn_wcm01_fir15_siglog100_fit05h_fit05c
557	25	0.161	0.231	0.288	ozgf01fb12x200_dlogbz_wc01_p1z1_dexp_fit10
558	25	0.168	0.236	0.290	ozgf01fb12x200_dlogb_wc01_p1z1_siglog_fit10
559	25	0.156	0.215	0.264	ozgf01fb12x200_dlogb_wc01_p1z1_sigrich_fit10
560	25	0.179	0.272	0.337	ozgf01fb12x200_invlinz_wc01_p1z1_dexp_fit10
561	25	0.183	0.266	0.329	ozgf01fb12x200_invlin_wc01_p1z1_siglog_fit10
562	25	0.184	0.271	0.333	ozgf01fb12x200_invlin_wc01_p1z1_sigrich_fit10
563	25	0.187	0.276	0.380	ozgf01fb12x200_lognbz_wc01_p1z1_dexp_fit10
564	25	0.151	0.215	0.295	ozgf01fb12x200_lognb_wc01_p1z1_sigcauchy_fit10
565	25	0.150	0.211	0.291	ozgf01fb12x200_lognb_wc01_p1z1_sigell_fit10
566	25	0.163	0.235	0.325	ozgf01fb12x200_lognb_wc01_p1z1_sigerf_fit10
567	25	0.180	0.261	0.363	ozgf01fb12x200_lognb_wc01_p1z1_siglog_fit10
568	25	0.175	0.250	0.350	ozgf01fb12x200_lognb_wc01_p1z1_sigrich_fit10
569	25	0.157	0.227	0.313	ozgf01fb12x200_lognb_wc01_p1z1_sigumbel_fit10
570	25	0.104	0.149	0.210	ozgf01fb12x200_lognb_wc01_p1z1_sigumber_fit10
571	25	0.187	0.278	0.343	ozgf01fb12x200_rootnbz_wc01_p1z1_dexp_fit10
572	25	0.175	0.258	0.359	ozgf01fb12x200_rootnb_wc01_p1z1_sigerf_fit10
573	25	0.178	0.267	0.329	ozgf01fb12x200_rootnb_wc01_p1z1_siglog_fit10
574	25	0.181	0.269	0.376	ozgf01fb12x200_rootnb_wc01_p1z1_sigrich_fit10
575	25	0.193	0.283	0.349	ozgf04fb12x200_lognbz_wc01_p1z1_dexp_fit09
576	25	0.111	0.161	0.198	ozgf04fb12x200_lognbz_wc01_p2z0_dexp_fit09
577	26	0.173	0.259	0.359	apgt04x200_fir20_siglog100_fit10
578	26	0.170	0.253	0.352	apgt04x200_sigcauchy_fir20_fit10
579	26	0.171	0.253	0.352	apgt04x200_sigell_fir20_fit10
580	26	0.169	0.252	0.351	apgt04x200_sigerf_fir20_fit10
581	26	0.166	0.248	0.345	apgt04x200_siglog100_fir20_fit10
582	26	0.168	0.249	0.345	apgt04x200_siglog_fir20_fit10
583	26	0.167	0.247	0.343	apgt04x200_sigrich_fir20_fit10
584	26	0.169	0.253	0.352	apgt04x200_sigumbel_fir20_fit10
585	26	0.168	0.251	0.351	apgt04x200_sigumber_fir20_fit10
586	26	0.284	0.387	0.466	fb18ch100_lognn_wcg03_ap2z1_dexp_fit09c
587	26	0.136	0.179	0.215	fb18ch100_lognn_wcg03_ap3z1_dlog_fit09c
588	26	0.243	0.337	0.409	fb18ch100_lognn_wcg03_ap3z1_lin_fit09c
589	26	0.157	0.212	0.255	fb18ch100_lognn_wcg03_ap3z1_logn_fit09c
590	26	0.139	0.183	0.222	fb18ch100_lognn_wcg03_ap3z1_rootn_fit09c
591	26	0.257	0.359	0.432	fb18ch100_lognn_wcg03_ap3z1_zthresh_fit09c
592	26	0.270	0.385	0.465	fb18ch100_lognn_wcg04_fir15g_dexp_fit09c
593	26	0.262	0.378	0.456	fb18ch100_lognn_wcgd01_fir15_siglog100_fit05h_fit05c
594	26	0.173	0.256	0.316	ozgf01fb12x200_dlogbz_wc01_p1z1_siglog_fit10
595	26	0.168	0.243	0.299	ozgf01fb12x200_dlogbz_wc01_p1z1_sigrich_fit10
596	26	0.188	0.281	0.349	ozgf01fb12x200_invlinz_wc01_p1z1_siglog_fit10
597	26	0.190	0.280	0.346	ozgf01fb12x200_invlinz_wc01_p1z1_sigrich_fit10
598	26	0.186	0.279	0.371	ozgf01fb12x200_lognbz_wc01_p1z1_siglog_fit10
599	26	0.190	0.281	0.375	ozgf01fb12x200_lognbz_wc01_p1z1_sigrich_fit10
600	26	0.177	0.261	0.324	ozgf01fb12x200_rootnbz_wc01_p1z1_siglog_fit10
601	26	0.187	0.279	0.346	ozgf01fb12x200_rootnbz_wc01_p1z1_sigrich_fit10
602	26	0.257	0.350	0.422	ozgf04fb12x100_lognbz_wc01_p2z1_dexp_fit09
603	26	0.200	0.302	0.373	ozgf04fb12x200_lognbz_wc01_p2z1_dexp_fit09

604	26	0.113	0.179	0.221	ozgf04fb12x200_lognbz_wc01_p3z0_dexp_fit09
605	26	0.174	0.262	0.364	ozgf04x200_fir20_siglog100_fit10
606	26	0.174	0.259	0.359	szgf24x200_fir20_siglog100_fit10
607	27	0.172	0.257	0.358	apgt04x200_lin_fir20_siglog_fit10
608	27	0.169	0.256	0.356	apgt04x200_logn_fir20_siglog_fit10
609	27	0.262	0.360	0.432	fb18ch100_lognn_wcg02_dexppc_ap3z1_dexp_fit09c
610	27	0.221	0.312	0.377	fb18ch100_lognn_wcg03_ap3z1_dlogb_fit09c
611	27	0.236	0.326	0.394	fb18ch100_lognn_wcg03_ap3z1_exp_fit09c
612	27	0.250	0.349	0.421	fb18ch100_lognn_wcg03_ap3z1_invlin_fit09c
613	27	0.211	0.298	0.358	fb18ch100_lognn_wcg03_ap3z1_lognb_fit09c
614	27	0.261	0.360	0.434	fb18ch100_lognn_wcg03_ap3z1_poly1_fit09c
615	27	0.239	0.330	0.401	fb18ch100_lognn_wcg03_ap3z1_rootnb_fit09c
616	27	0.246	0.338	0.407	fb18ch100_lognn_wcm03_fir15m_dexp_fit09c
617	27	0.271	0.372	0.448	fb18ch100_lognzn_wcg03_ap3z1_dexp_fit09c
618	27	0.194	0.284	0.350	ozgf04fb12x200_lognbz_wc01_p1z0x2_dexp_fit09
619	27	0.195	0.284	0.350	ozgf04fb12x200_lognbz_wc01_p2z2_dexp_fit09
620	27	0.171	0.253	0.313	ozgf04fb12x200_lognbz_wc01_p3z1_dexp_fit09
621	28	0.169	0.256	0.356	apgt04x200_lognb_fir20_siglog_fit10
622	28	0.169	0.250	0.346	apgt04x200_poly1_fir20_siglog_fit10
623	28	0.218	0.313	0.377	fb18ch100_lognn_wcg03_ap3z1_dlogbz_fit09c
624	28	0.268	0.372	0.448	fb18ch100_lognn_wcg03_ap3z1_invlinz_fit09c
625	28	0.211	0.292	0.350	fb18ch100_lognn_wcg03_ap3z1_lognbz_fit09c
626	28	0.266	0.374	0.451	fb18ch100_lognn_wcg03_ap3z1_poly2_fit09c
627	28	0.276	0.389	0.469	fb18ch100_lognn_wcg03_ap3z1_rootnbz_fit09c
628	28	0.202	0.305	0.377	ozgf04fb12x200_lognbz_wc01_p3z2_dexp_fit09
629	29	0.178	0.264	0.369	apgt04x200_lognbz_fir20_siglog_fit10
630	29	0.165	0.243	0.337	apgt04x200_poly2_fir20_siglog_fit10
631	29	0.277	0.303	0.365	fb18ch100_lognn_wcg03_ap2z2_dexp_fit09c
632	29	0.283	0.399	0.481	fb18ch100_lognn_wcg03_ap3z1i_dexp_fit09c
633	29	0.263	0.378	0.455	fb18ch100_lognn_wcg03_ap3z1_dexp_fit05h
634	29	0.269	0.385	0.464	fb18ch100_lognn_wcg03_ap3z1_dexp_fit05h_fit05c
635	29	0.286	0.401	0.483	fb18ch100_lognn_wcg03_ap3z1_dexp_fit05h_fit09c
636	29	0.289	0.393	0.474	fb18ch100_lognn_wcg03_ap3z1_dexp_fit05h_fit19g
637	29	0.209	0.284	0.342	fb18ch100_lognn_wcg03_ap3z1_dexp_fit05h_fit21
638	29	0.287	0.393	0.474	fb18ch100_lognn_wcg03_ap3z1_dexp_fit05i_fit09c
639	29	0.285	0.394	0.475	fb18ch100_lognn_wcg03_ap3z1_dexp_fit05j_fit09c
640	29	0.265	0.380	0.457	fb18ch100_lognn_wcg03_ap3z1_dexp_fit05k
641	29	0.270	0.388	0.468	fb18ch100_lognn_wcg03_ap3z1_dexp_fit05m
642	29	0.264	0.380	0.457	fb18ch100_lognn_wcg03_ap3z1_dexp_fit05n
643	29	0.262	0.377	0.454	fb18ch100_lognn_wcg03_ap3z1_dexp_fit05o
644	29	0.274	0.390	0.470	fb18ch100_lognn_wcg03_ap3z1_dexp_fit05p
645	29	0.277	0.392	0.473	fb18ch100_lognn_wcg03_ap3z1_dexp_fit05q
646	29	0.280	0.395	0.476	fb18ch100_lognn_wcg03_ap3z1_dexp_fit05r
647	29	0.287	0.401	0.483	fb18ch100_lognn_wcg03_ap3z1_dexp_fit05t
648	29	0.285	0.394	0.474	fb18ch100_lognn_wcg03_ap3z1_dexp_fit05t1
649	29	0.284	0.394	0.474	fb18ch100_lognn_wcg03_ap3z1_dexp_fit05t2
650	29	0.285	0.396	0.477	fb18ch100_lognn_wcg03_ap3z1_dexp_fit05t3
651	29	0.280	0.388	0.468	fb18ch100_lognn_wcg03_ap3z1_dexp_fit05t4
652	29	0.282	0.392	0.473	fb18ch100_lognn_wcg03_ap3z1_dexp_fit05u
653	29	0.282	0.392	0.473	fb18ch100_lognn_wcg03_ap3z1_dexp_fit05u1
654	29	0.280	0.391	0.472	fb18ch100_lognn_wcg03_ap3z1_dexp_fit05u2
655	29	0.282	0.396	0.477	fb18ch100_lognn_wcg03_ap3z1_dexp_fit05u3
656	29	0.283	0.390	0.470	fb18ch100_lognn_wcg03_ap3z1_dexp_fit05u4
657	29	0.283	0.396	0.477	fb18ch100_lognn_wcg03_ap3z1_dexp_fit05u5
658	29	0.286	0.394	0.475	fb18ch100_lognn_wcg03_ap3z1_dexp_fit05u6

659	29	0.286	0.395	0.476	fb18ch100_lognn_wcg03_ap3z1_dexp_fit05u7
660	29	0.283	0.396	0.477	fb18ch100_lognn_wcg03_ap3z1_dexp_fit05ua
661	29	0.281	0.397	0.477	fb18ch100_lognn_wcg03_ap3z1_dexp_fit05ub
662	29	0.284	0.394	0.475	fb18ch100_lognn_wcg03_ap3z1_dexp_fit05v1
663	29	0.284	0.397	0.478	fb18ch100_lognn_wcg03_ap3z1_dexp_fit05v2
664	29	0.287	0.398	0.480	fb18ch100_lognn_wcg03_ap3z1_dexp_fit05v3
665	29	0.286	0.401	0.484	fb18ch100_lognn_wcg03_ap3z1_dexp_fit05v4
666	29	0.284	0.398	0.480	fb18ch100_lognn_wcg03_ap3z1_dexp_fit05v5
667	29	0.286	0.396	0.478	fb18ch100_lognn_wcg03_ap3z1_dexp_fit05v7
668	29	0.285	0.397	0.479	fb18ch100_lognn_wcg03_ap3z1_dexp_fit05v8
669	29	0.284	0.390	0.469	fb18ch100_lognn_wcg03_ap3z1_dexp_fit05vb
670	29	0.284	0.393	0.474	fb18ch100_lognn_wcg03_ap3z1_dexp_fit05vc
671	29	0.250	0.364	0.438	fb18ch100_lognn_wcg03_ap3z1_dexp_fit05w30
672	29	0.263	0.382	0.460	fb18ch100_lognn_wcg03_ap3z1_dexp_fit05w35
673	29	0.270	0.390	0.470	fb18ch100_lognn_wcg03_ap3z1_dexp_fit05w40
674	29	0.277	0.395	0.476	fb18ch100_lognn_wcg03_ap3z1_dexp_fit05w45
675	29	0.282	0.400	0.482	fb18ch100_lognn_wcg03_ap3z1_dexp_fit05w50
676	29	0.284	0.401	0.484	fb18ch100_lognn_wcg03_ap3z1_dexp_fit05w55
677	29	0.286	0.401	0.484	fb18ch100_lognn_wcg03_ap3z1_dexp_fit05w60
678	29	0.288	0.402	0.485	fb18ch100_lognn_wcg03_ap3z1_dexp_fit05w65
679	29	0.224	0.323	0.393	fb18ch100_lognn_wcg03_ap3z1_dexp_fit05x
680	29	0.275	0.392	0.473	fb18ch100_lognn_wcg03_ap3z1_dexp_fit05x0
681	29	0.266	0.383	0.462	fb18ch100_lognn_wcg03_ap3z1_dexp_fit05x0_40
682	29	0.224	0.323	0.393	fb18ch100_lognn_wcg03_ap3z1_dexp_fit05x2
683	29	0.173	0.249	0.303	fb18ch100_lognn_wcg03_ap3z1_dexp_fit05x2_40
684	29	0.263	0.380	0.456	fb18ch100_lognn_wcg03_ap3z1_dexp_fit05x2_60
685	29	0.218	0.316	0.382	fb18ch100_lognn_wcg03_ap3z1_dexp_fit05x3
686	29	0.168	0.244	0.296	fb18ch100_lognn_wcg03_ap3z1_dexp_fit05x3_40
687	29	0.218	0.316	0.383	fb18ch100_lognn_wcg03_ap3z1_dexp_fit05x4
688	29	0.168	0.244	0.296	fb18ch100_lognn_wcg03_ap3z1_dexp_fit05x4_40
689	29	0.173	0.249	0.303	fb18ch100_lognn_wcg03_ap3z1_dexp_fit05x_40
690	29	0.285	0.394	0.474	fb18ch100_lognn_wcg03_ap3z1_dexp_fit09c
691	29	0.270	0.380	0.457	fb18ch100_lognn_wcg03_ap3z1_dexp_fit20a
692	29	0.272	0.380	0.457	fb18ch100_lognn_wcg03_ap3z1_dexp_fit20b
693	29	0.104	0.134	0.165	fb18ch100_lognn_wcg03_ap3z1_dexp_fit21
694	29	0.286	0.402	0.485	fb18ch100_lognn_wcg03_ap3z1_dexp_fit23
695	29	0.283	0.396	0.477	fb18ch100_lognn_wcg03_ap3z1_dexp_fit23a
696	29	0.283	0.393	0.474	fb18ch100_lognn_wcg03_ap3z1_dexp_fit23b
697	29	0.281	0.395	0.477	fb18ch100_lognn_wcg03_ap3z1_dexp_fit23c
698	29	0.281	0.393	0.473	fb18ch100_lognn_wcg03_ap3z1_dexp_fit23d
699	29	0.267	0.370	0.446	fb18ch100_lognn_wcg03_ap3z1_poly3_fit09c
700	29	0.255	0.354	0.427	fb18ch100_lognn_wcg03_ap3z1_sig_fit09c
701	29	0.284	0.391	0.471	fb18ch100_lognn_wcg03_ap3z1_zsoft_fit09c
702	29	0.279	0.388	0.468	fb18ch100_lognn_wcg04_ap2z0_dexp_fit09c
703	29	0.269	0.350	0.421	fb18ch100_lognn_wcm04_ap1z0_dexp_fit09c
704	29	0.222	0.336	0.413	fb18ch200_lognn_wcg03_ap3z1_dexp_fit09c
705	29	0.166	0.273	0.329	fb18ch400_lognn_wcg03_ap3z1_dexp_fit09c
706	29	0.284	0.387	0.467	fb24ch100_lognn_wcg03_ap3z1_dexp_fit09c
707	29	0.283	0.394	0.475	fb36ch100_lognn_wcg03_ap3z1_dexp_fit09c
708	29	0.224	0.323	0.390	gt18ch100_wc01_p1z1_siglog100_fit05
709	29	0.238	0.337	0.405	gt18ch100_wc01_p1z1_siglog100_fit15rnd
710	29	0.229	0.320	0.386	gt18ch100_wc01_p1z1_siglog100_fitgen00
711	29	0.239	0.336	0.405	gt18ch100_wc01_p1z1_siglog100_prefitrnd_fit05c
712	29	0.191	0.281	0.345	ozgf04fb12x200_lognbz_wc01_p3z3_dexp_fit09
713	30	0.174	0.257	0.357	apgt04x200_poly3_fir20_siglog_fit10

714	30	0.267	0.370	0.445	fb18ch100_lognn_wcg03_ap3z1_sigcauchy_fit09c
715	30	0.269	0.373	0.450	fb18ch100_lognn_wcg03_ap3z1_sigell_fit09c
716	30	0.241	0.334	0.402	fb18ch100_lognn_wcg03_ap3z1_sigerf_fit09c
717	30	0.277	0.384	0.462	fb18ch100_lognn_wcg03_ap3z1_siglog100_fit09c
718	30	0.283	0.390	0.470	fb18ch100_lognn_wcg03_ap3z1_siglog_fit09c
719	30	0.284	0.391	0.472	fb18ch100_lognn_wcg03_ap3z1_sigrich_fit09c
720	30	0.253	0.346	0.418	fb18ch100_lognn_wcg03_ap3z1_sigumbel_fit09c
721	30	0.248	0.350	0.422	fb18ch100_lognn_wcg03_ap3z1_sigumber_fit09c
722	30	0.261	0.372	0.448	fb18ch100_lognn_wcg03_fir15p_dexp_fit09c
723	30	0.240	0.337	0.410	fb18ch100_lognn_wcg04_fir15m_dexp_fit09c
724	30	0.284	0.381	0.460	fb18ch100_lognn_wcg05_ap1z0_dexp_fit09c
725	30	0.255	0.355	0.427	fb18ch100_lognn_wcm04_fir15g_dexp_fit09c
726	30	0.199	0.292	0.360	ozgf04fb12x200_lognbz_wc01_p1z0x3_dexp_fit09
727	31	0.261	0.372	0.449	fb18ch100_lognbn_wcg03_fir15p_dexp_fit09c
728	31	0.283	0.395	0.476	fb18ch100_lognn_wcg03_fir15p_siglog100_fit05h_fit19g
729	31	0.198	0.295	0.364	ozgf04fb12x200_lognbz_wc01_p4z4_dexp_fit09
730	32	0.263	0.382	0.460	fb18ch100_lognbn_wcg03_fir15p_siglog100_fit05d
731	32	0.262	0.383	0.460	fb18ch100_lognbn_wcg03_fir15p_siglog100_fit05h_fit05c
732	32	0.267	0.387	0.465	fb18ch100_lognbn_wcg03_fir15p_siglog100_fit05h_fit05d
733	32	0.285	0.392	0.473	fb18ch100_lognbn_wcg03_fir15p_siglog100_fit05h_fit19b
734	32	0.285	0.393	0.474	fb18ch100_lognbn_wcg03_fir15p_siglog100_fit05h_fit19f
735	32	0.283	0.393	0.474	fb18ch100_lognbn_wcg03_fir15p_siglog100_fit05h_fit19g
736	32	0.283	0.391	0.471	fb18ch100_lognbn_wcg03_fir15p_siglog100_fit05j_fit19f
737	32	0.276	0.386	0.466	fb18ch100_lognbn_wcg03_fir15p_siglog100_fit05j_fit19g
738	32	0.262	0.379	0.456	fb18ch100_lognbn_wcg03_fir15p_siglog100_fit09c
739	32	0.285	0.386	0.464	fb18ch100_lognn_wcg03_ap3z2_dexp_fit09c
740	32	0.283	0.392	0.472	fb18ch100_lognn_wcg03_ap4z1_dexp_fit09c
741	32	0.289	0.373	0.450	fb18ch100_lognn_wcm03_ap3z1_dexp_fit05h_fit19g
742	32	0.285	0.375	0.452	fb18ch100_lognn_wcm03_ap3z1_dexp_fit09c
743	33	0.287	0.385	0.464	fb18ch100_lognn_wcg04_ap2z1_dexp_fit09c
744	33	0.279	0.376	0.453	fb18ch100_lognn_wcm03_fir15p_dexp_fit05h_fit19g
745	33	0.252	0.346	0.420	fb18ch100_lognn_wcm03_fir15p_dexp_fit09c
746	33	0.201	0.292	0.361	ozgf04fb12x200_lognbz_wc01_p1z0x4_dexp_fit09
747	34	0.239	0.333	0.402	fb18ch100_lognn_wcm04_fir15m_dexp_fit09c
748	34	0.237	0.340	0.409	gt12ch100_logfree_wc01_fir15_dexp_fit05a
749	34	0.159	0.254	0.356	gt12ch200_wc01_fir20_fit10
750	35	0.286	0.396	0.476	fb18ch100_lognn_wcg03_ap4z2_dexp_fit09c
751	35	0.284	0.396	0.478	fb18ch100_lognn_wcg05_ap2z0_dexp_fit09c
752	35	0.287	0.385	0.464	fb18ch100_lognn_wcg06_ap1z0_dexp_fit09c
753	35	0.222	0.337	0.415	fb18ch200_lognn_wcg05_ap2z0_dexp_fit09c
754	35	0.166	0.272	0.327	fb18ch400_lognn_wcg05_ap2z0_dexp_fit09c
755	35	0.285	0.390	0.469	fb24ch100_lognn_wcg05_ap2z0_dexp_fit09c
756	35	0.283	0.393	0.474	fb36ch100_lognn_wcg05_ap2z0_dexp_fit09c
757	35	0.234	0.334	0.401	gt12ch100_lognbn_wc01_fir15_dexp_fit05a
758	35	0.185	0.280	0.390	gt12ch200_logn_wc01_fir20_fit10
759	36	0.257	0.363	0.438	fb18ch100_lognn_wcg02_fir15_fit05c
760	37	0.245	0.347	0.418	fb18ch100_lognn_doublewcg01fir15b_combaverage_fit05c
761	37	0.202	0.276	0.334	fb18ch100_lognn_doublewcg01fir15b_npnl2b_fit05c
762	37	0.200	0.273	0.330	fb18ch100_lognn_doublewcg01fir15b_npnl2_fit05c
763	37	0.256	0.359	0.432	fb18ch100_lognn_doublewcg01fir15_combaverage_fit05c
764	37	0.101	0.125	0.146	fb18ch100_lognn_doublewcg01fir15_npnl2b_fit05c
765	37	0.100	0.118	0.142	fb18ch100_lognn_doublewcg01fir15_npnl2_fit05c
766	37	0.288	0.396	0.477	fb18ch100_lognn_wcg04_ap3z1_dexp_fit05vb
767	37	0.288	0.396	0.477	fb18ch100_lognn_wcg04_ap3z1_dexp_fit05vc
768	37	0.288	0.393	0.474	fb18ch100_lognn_wcg04_ap3z1_dexp_fit09c

769	37	0.274	0.384	0.462	fb18ch100_lognn_wcg04_ap3z1_dexp_fit20a
770	37	0.282	0.394	0.475	fb18ch100_lognn_wcg04_ap3z1_dexp_fit23c
771	37	0.282	0.391	0.471	fb18ch100_lognn_wcg04_ap3z1_dexp_fit23d
772	38	0.269	0.387	0.466	fb18ch100_lognn_wcg04_fir15p_dexp_fit09c
773	39	0.288	0.394	0.476	fb18ch100_lognn_wcg04_fir15p_siglog100_fit05h_fit19g
774	39	0.202	0.303	0.421	gt12ch200_logn_wc01_fir20_dexp_fit10
775	39	0.192	0.292	0.406	ozgf04fb12x200_logn_wc01_fir20_dexp_fit10
776	40	0.284	0.382	0.462	fb18ch100_lognn_wc01_fir15_dexp_fit05vb
777	40	0.284	0.383	0.463	fb18ch100_lognn_wc01_fir15_dexp_fit05vc
778	40	0.285	0.394	0.475	fb18ch100_lognn_wcg02_fir15_dexp_fit05vb
779	40	0.292	0.383	0.463	fb18ch100_lognn_wcg05_ap2z1_dexp_fit09c
780	40	0.258	0.366	0.441	fb18ch100_normp_logn_wcg02a_fir15_dexp_fit09
781	40	0.259	0.369	0.444	fb18ch100_normp_logn_wcg02_fir15_dexp_fit09
782	40	0.189	0.293	0.408	gt12ch200_logfree_wc01_fir20_siglog100_fit05
783	40	0.199	0.302	0.422	gt12ch200_logn_wc01_fir20_siglog_fit10
784	40	0.236	0.298	0.358	gt18ch100_logn_nsepfir15_siglog100b_fit05c
785	40	0.220	0.278	0.333	gt18ch100_logn_nsepfir15_siglog100b_fit05g_fit05c
786	40	0.236	0.295	0.355	gt18ch100_logn_nsepfir15_siglog100b_fitgen02
787	40	0.193	0.292	0.408	ozgf04fb12x200_logn_wc01_fir20_siglog_fit10
788	41	0.280	0.376	0.454	fb18ch100_lognn_wc01_fir15_siglog100_fit05h_fit05c
789	41	0.277	0.394	0.475	fb18ch100_lognn_wcg02_fir15_siglog100b_fit05c
790	41	0.276	0.393	0.475	fb18ch100_lognn_wcg02_fir15_siglog100_fit05h
791	41	0.281	0.394	0.475	fb18ch100_lognn_wcg02_fir15_siglog100_fit05h_fit05c
792	41	0.285	0.390	0.471	fb18ch100_lognn_wcg06_ap2z0_dexp_fit09c
793	41	0.294	0.378	0.455	fb18ch100_lognn_wcm04_ap3z1_dexp_fit09c
794	41	0.273	0.391	0.473	fb18ch100_normp_logn_wcg02a_fir15_siglog100_fit05g_fit05c
795	41	0.272	0.393	0.474	fb18ch100_normp_logn_wcg02_fir15_siglog100_fit05g_fit05c
796	41	0.268	0.391	0.472	fb18ch100_normp_logn_wcg02_root2_fir15_siglog100_fit05g_fit05c
797	41	0.271	0.390	0.471	fb18ch100_normp_logn_wcgr02_fir15_siglog100_fit05g_fit05c
798	41	0.200	0.297	0.415	gt12ch200_lognbz_wc01_fir20_dexp_fit10
799	41	0.209	0.277	0.341	gt12ch200_logn_wc02_p1z1_siglog_fit10
800	41	0.218	0.285	0.343	gt18ch100_logn_sepfir15_siglog100b_fit05c
801	41	0.212	0.283	0.342	gt18ch100_logn_sepfir15_siglog100b_fit05g_fit05c
802	41	0.222	0.265	0.320	gt18ch100_logn_sepfir15_siglog100b_fitgen02
803	41	0.195	0.295	0.412	ozgf01fb12x200a_lognbz_wc01_fir20_dexp_fit10
804	41	0.189	0.290	0.404	ozgf01fb12x200b_lognbz_wc01_fir20_dexp_fit10
805	41	0.197	0.297	0.414	ozgf01fb12x200c_lognbz_wc01_fir20_dexp_fit10
806	41	0.195	0.295	0.412	ozgf01fb12x200_lognbz_wc01_fir20_dexp_fit10
807	41	0.200	0.301	0.418	ozgf04fb12x200_lognbz_wc01_fir20_dexp_fit10
808	42	0.280	0.395	0.477	fb18ch100_lognbn_wcg02_fir15_siglog100_fit05h_fit05c
809	42	0.259	0.359	0.433	fb18ch100_lognn_wcm04_fir15p_dexp_fit09c
810	42	0.248	0.352	0.424	fb18ch100_normp_logn_wcm02a_fir15_dexp_fit09
811	42	0.254	0.365	0.441	fb18ch100_normp_logn_wcm02_fir15_dexp_fit09
812	42	0.204	0.300	0.419	gt12ch200_lognbz_wc01_fir20_siglog_fit10
813	42	0.189	0.285	0.398	ozgf04fb12x200_dlogbz_wc01_fir20_siglog_fit10
814	43	0.280	0.396	0.478	fb18ch100_lognn_wcgr02_fir15_siglog100_fit05h_fit05c
815	43	0.282	0.393	0.475	fb18ch100_lognn_wcm02_fir15_siglog100_fit05h_fit05c
816	43	0.272	0.392	0.472	fb18ch100_normp_logn_wcm02a_fir15_siglog100_fit05g_fit05c
817	43	0.270	0.385	0.464	fb18ch100_normp_logn_wcm02_fir15_siglog100_fit05g_fit05c
818	44	0.253	0.361	0.435	fb18ch100_normp_logn_wcgd02a_fir15_dexp_fit09
819	44	0.252	0.364	0.440	fb18ch100_normp_logn_wcgd02_fir15_dexp_fit09
820	44	0.277	0.360	0.433	ozgf04fb12x100_lognbz_wc02_p2z1_dexp_fit09
821	44	0.275	0.362	0.436	ozgf04fb12x100_lognbz_wc02_p2z1_dexp_prefitrnd_fit09
822	45	0.292	0.399	0.481	fb18ch100_lognn_wcg05_ap3z1_dexp_fit05vb
823	45	0.293	0.398	0.480	fb18ch100_lognn_wcg05_ap3z1_dexp_fit05vc

824	45	0.293	0.393	0.474	fb18ch100_lognn_wcg05_ap3z1_dexp_fit09c
825	45	0.279	0.389	0.470	fb18ch100_lognn_wcgd02_fir15_siglog100_fit05h_fit05c
826	45	0.271	0.389	0.470	fb18ch100_normp_logn_wcgd02a_fir15_siglog100_fit05g_fit05c
827	45	0.269	0.384	0.464	fb18ch100_normp_logn_wcgd02_fir15_siglog100_fit05g_fit05c
828	45	0.274	0.366	0.440	gt18ch100_logfree_nsepfir20_siglog100_prefitrnd_fit05c
829	45	0.210	0.308	0.380	gt18ch200_logn_wc01_fir20_dexp_fit09
830	46	0.218	0.296	0.356	gt18ch100_logfree_sepfir20_siglog100_fit05
831	46	0.261	0.358	0.430	gt18ch100_logfree_sepfir20_siglog100_prefitrnd_fit05
832	46	0.265	0.377	0.456	fb18ch100_lognn_doublewcg01fir15b_combpoly2_fit05c
833	46	0.244	0.348	0.420	fb18ch100_lognn_doublewcg01fir15b_combpoly2_fit09c
834	46	0.265	0.377	0.454	fb18ch100_lognn_doublewcg01fir15_combpoly2_fit05c
835	46	0.246	0.352	0.425	fb18ch100_lognn_doublewcg01fir15_combpoly2_fit09c
836	46	0.269	0.366	0.441	gt18ch100_logfree_sepfir20_siglog100_prefitrnd_fit05c
837	46	0.262	0.368	0.461	gt18ch100_logfree_wc01_fir20_siglog100_fit05
838	46	0.265	0.364	0.440	gt18ch100_logfree_wc01_fir20_siglog100_prefitrnd_fit05
839	47	0.260	0.354	0.427	fb18ch100_lognbz_wc01_fir20_dexp_fit09
840	47	0.296	0.378	0.456	fb18ch100_lognn_wcg06_ap2z1_dexp_fit09c
841	47	0.260	0.355	0.428	fb18ch100_norm_lognbz_wc01_fir20_dexp_fit09
842	47	0.262	0.356	0.430	gt18ch100_lognbz_wc01_fir20_dexp_fit09
843	47	0.208	0.301	0.372	gt18ch200_lognbz_wc01_fir20_dexp_fit09
844	47	0.115	0.161	0.223	gt36ch200_logfree_wc01_p1z0_siglog100_fit10
845	47	0.248	0.332	0.399	ozgf04fb18x100_lognbz_wc01_fir20_dexp_fit09
846	47	0.207	0.301	0.372	ozgf04fb18x200a_lognbz_wc01_fir20_dexp_fit09
847	47	0.207	0.302	0.372	ozgf04fb18x200b_lognbz_wc01_fir20_dexp_fit09
848	47	0.209	0.307	0.379	ozgf04fb18x200c_lognbz_wc01_fir20_dexp_fit09
849	47	0.208	0.306	0.377	ozgf04fb18x200d_lognbz_wc01_fir20_dexp_fit09
850	47	0.207	0.305	0.377	ozgf04fb18x200e_lognbz_wc01_fir20_dexp_fit09
851	47	0.207	0.303	0.374	ozgf04fb18x200f_lognbz_wc01_fir20_dexp_fit09
852	47	0.206	0.304	0.376	ozgf04fb18x200g_lognbz_wc01_fir20_dexp_fit09
853	47	0.204	0.296	0.365	ozgf04fb18x200_lognbz_wc01_fir20_dexp_fit09
854	48	0.216	0.310	0.431	gt36ch200_logfree_wc01_p1z1_siglog100_fit10
855	48	0.116	0.161	0.224	gt36ch200_logfree_wc01_p2z0_siglog100_fit10
856	48	0.211	0.299	0.369	gt36ch200_logn_wc01_p1z1_siglog_fit10
857	49	0.177	0.253	0.351	gt36ch200_logfree_wc01_p2z1_siglog100_fit10
858	49	0.084	0.141	0.198	gt36ch200_logfree_wc01_p3z0_siglog100_fit10
859	50	0.220	0.303	0.419	gt36ch200_logfree_wc01_p1z0x2_siglog100_fit10
860	50	0.220	0.312	0.435	gt36ch200_logfree_wc01_p2z2_siglog100_fit10
861	50	0.122	0.184	0.258	gt36ch200_logfree_wc01_p3z1_siglog100_fit10
862	51	0.198	0.286	0.399	gt36ch200_logfree_wc01_p3z2_siglog100_fit10
863	51	0.199	0.298	0.368	ozgf04fb12x200_lognbz_wc01_fir30_dexp_fit09
864	52	0.123	0.193	0.267	gt36ch200_logfree_wc01_p2z0x2_siglog100_fit10
865	52	0.219	0.312	0.435	gt36ch200_logfree_wc01_p3z3_siglog100_fit10
866	53	0.265	0.370	0.444	fb18ch100_lognn_wcg03_fir15_fit05c
867	54	0.220	0.309	0.430	gt36ch200_logfree_wc01_p4z4_siglog100_fit10
868	57	0.295	0.393	0.474	fb18ch100_lognn_wcg03_fir15_dexp_fit05vb
869	57	0.242	0.355	0.427	fb18ch100_lognn_wcg03_fir15_dexp_fit05w30
870	57	0.263	0.381	0.458	fb18ch100_lognn_wcg03_fir15_dexp_fit05w35
871	57	0.274	0.391	0.470	fb18ch100_lognn_wcg03_fir15_dexp_fit05w40
872	57	0.279	0.395	0.476	fb18ch100_lognn_wcg03_fir15_dexp_fit05w45
873	57	0.286	0.398	0.480	fb18ch100_lognn_wcg03_fir15_dexp_fit05w50
874	57	0.291	0.398	0.480	fb18ch100_lognn_wcg03_fir15_dexp_fit05w55
875	57	0.294	0.395	0.476	fb18ch100_lognn_wcg03_fir15_dexp_fit23
876	57	0.294	0.396	0.477	fb18ch100_lognn_wcg03_fir15_dexp_fit23a
877	57	0.295	0.391	0.472	fb18ch100_lognn_wcg03_fir15_dexp_fit23b
878	57	0.293	0.396	0.478	fb18ch100_lognn_wcg03_fir15_dexp_fit23c

879	58	0.284	0.394	0.475	fb18ch100_lognn_wcg03_fir15_siglog100_fit05h
880	58	0.288	0.396	0.477	fb18ch100_lognn_wcg03_fir15_siglog100_fit05h_fit05c
881	58	0.221	0.331	0.408	fb18ch200_lognn_wcg03_fir15_siglog100_fit05h_fit05c
882	58	0.155	0.251	0.303	fb18ch400_lognn_wcg03_fir15_siglog100_fit05h_fit05c
883	58	0.288	0.396	0.477	fb24ch100_lognn_wcg03_fir15_siglog100_fit05h_fit05c
884	58	0.289	0.395	0.476	fb36ch100_lognn_wcg03_fir15_siglog100_fit05h_fit05c
885	58	0.216	0.288	0.355	gt12ch200_logn_wc03_p1z1_siglog_fit10
886	59	0.293	0.392	0.472	fb18ch100_lognbn_wcg03_fir15_siglog100_fit05d
887	59	0.288	0.396	0.478	fb18ch100_lognbn_wcg03_fir15_siglog100_fit05h_fit05c
888	59	0.292	0.393	0.474	fb18ch100_lognbn_wcg03_fir15_siglog100_fit05h_fit05d
889	59	0.298	0.385	0.464	fb18ch100_lognbn_wcg03_fir15_siglog100_fit05h_fit19b
890	59	0.295	0.389	0.469	fb18ch100_lognbn_wcg03_fir15_siglog100_fit05h_fit19f
891	59	0.293	0.395	0.477	fb18ch100_lognbn_wcg03_fir15_siglog100_fit09c
892	61	0.290	0.398	0.480	fb18ch100_lognn_wcm03_fir15_siglog100_fit05h_fit05c
893	62	0.283	0.359	0.432	ozgf04fb12x100_lognbz_wc03_p2z1_dexp_fit09
894	63	0.264	0.359	0.434	gt12ch100_lognbz_wc02_fir15_siglog100_fit05
895	63	0.264	0.362	0.437	gt12ch100_lognbz_wc02_fir15_siglog100_fit05a
896	63	0.275	0.365	0.441	gt12ch100_lognbz_wc02_fir15_siglog100_fit05a_fit05c
897	63	0.275	0.364	0.440	gt12ch100_lognbz_wc02_fir15_siglog100_fit05a_fit05s
898	63	0.218	0.312	0.434	gt36ch200_log2_wc01_fir20_siglog100_fit10
899	63	0.216	0.311	0.433	gt36ch200_root2_wc01_fir20_siglog100_fit10
900	63	0.206	0.300	0.418	gt36ch200_wc01_fir20_siglog100_fit10
901	64	0.286	0.393	0.474	fb18ch100_lognn_wcgd03_fir15_siglog100_fit05h_fit05c
902	64	0.218	0.311	0.434	gt36ch200_dlog_wc01_fir20_siglog100_fit10
903	64	0.205	0.299	0.417	gt36ch200_lin_wc01_fir20_siglog100_fit10
904	64	0.219	0.316	0.440	gt36ch200_logfree_wc01_fir20_siglog100_fit10
905	64	0.219	0.316	0.440	gt36ch200_logn_wc01_fir20_siglog100_fit10
906	64	0.216	0.309	0.382	gt36ch200_logn_wc01_fir20_siglog_fit10
907	64	0.215	0.308	0.430	gt36ch200_rootfree_wc01_fir20_siglog100_fit10
908	64	0.215	0.308	0.430	gt36ch200_rootn_wc01_fir20_siglog100_fit10
909	64	0.207	0.301	0.418	gt36ch200_zthresh_wc01_fir20_siglog100_fit10
910	65	0.219	0.314	0.439	gt36ch200_dlogb_wc01_fir20_siglog100_fit10
911	65	0.203	0.287	0.401	gt36ch200_exp_wc01_fir20_siglog100_fit10
912	65	0.209	0.305	0.425	gt36ch200_invlin_wc01_fir20_siglog100_fit10
913	65	0.220	0.315	0.439	gt36ch200_lognb_wc01_fir20_siglog100_fit10
914	65	0.207	0.299	0.417	gt36ch200_poly1_wc01_fir20_siglog100_fit10
915	65	0.215	0.308	0.429	gt36ch200_rootnb_wc01_fir20_siglog100_fit10
916	66	0.221	0.311	0.432	gt36ch200_dlogbz_wc01_fir20_siglog100_fit10
917	66	0.176	0.250	0.345	gt36ch200_invlinz_wc01_fir20_siglog100_fit10
918	66	0.220	0.311	0.435	gt36ch200_lognbz_wc01_fir20_siglog100_fit10
919	66	0.194	0.275	0.382	gt36ch200_poly2_wc01_fir20_siglog100_fit10
920	66	0.219	0.309	0.430	gt36ch200_rootnbz_wc01_fir20_siglog100_fit10
921	67	0.171	0.262	0.367	gt12ch200_wc02_fir20_fit10
922	67	0.210	0.303	0.421	gt36ch200_dexp_wc01_fir20_siglog100_fit10
923	67	0.193	0.268	0.373	gt36ch200_poly3_wc01_fir20_siglog100_fit10
924	67	0.212	0.309	0.431	gt36ch200_sig_wc01_fir20_siglog100_fit10
925	67	0.219	0.308	0.429	gt36ch200_zexp_wc01_fir20_siglog100_fit10
926	68	0.198	0.292	0.407	gt12ch200_logn_wc02_fir20_fit10
927	68	0.223	0.306	0.427	gt36ch200_sigcauchy_wc01_fir20_siglog100_fit10
928	68	0.222	0.308	0.430	gt36ch200_sigell_wc01_fir20_siglog100_fit10
929	68	0.213	0.305	0.425	gt36ch200_sigerf_wc01_fir20_siglog100_fit10
930	68	0.212	0.311	0.433	gt36ch200_siglog_wc01_fir20_siglog100_fit10
931	68	0.206	0.303	0.421	gt36ch200_sigrich_wc01_fir20_siglog100_fit10
932	68	0.214	0.305	0.425	gt36ch200_sigumbel_wc01_fir20_siglog100_fit10
933	68	0.215	0.302	0.422	gt36ch200_sigumber_wc01_fir20_siglog100_fit10

934	70	0.268	0.370	0.445	fb18ch100_lognn_wcg04_fir15_fit05c
935	73	0.259	0.355	0.443	gt12ch100_logfree_wc02_fir20_siglog100_fit05
936	73	0.214	0.312	0.436	gt12ch200_logn_wc02_fir20_siglog_fit10
937	74	0.305	0.382	0.460	fb18ch100_lognn_wc02_fir15_dexp_fit05vb
938	74	0.305	0.380	0.458	fb18ch100_lognn_wc02_fir15_dexp_fit05vc
939	74	0.261	0.368	0.442	fb18ch100_lognn_wc02_fir15_dexp_fit05w30
940	74	0.267	0.378	0.454	fb18ch100_lognn_wc02_fir15_dexp_fit05w35
941	74	0.279	0.386	0.464	fb18ch100_lognn_wc02_fir15_dexp_fit05w40
942	74	0.288	0.393	0.473	fb18ch100_lognn_wc02_fir15_dexp_fit05w45
943	74	0.295	0.392	0.472	fb18ch100_lognn_wc02_fir15_dexp_fit05w50
944	74	0.301	0.389	0.469	fb18ch100_lognn_wc02_fir15_dexp_fit05w55
945	74	0.305	0.385	0.464	fb18ch100_lognn_wc02_fir15_dexp_fit05w60
946	74	0.309	0.379	0.456	fb18ch100_lognn_wc02_fir15_dexp_fit05w65
947	74	0.264	0.370	0.446	fb18ch100_lognn_wc02_fir15_dexp_fit05x
948	74	0.308	0.377	0.455	fb18ch100_lognn_wc02_fir15_dexp_fit05x0
949	74	0.300	0.386	0.465	fb18ch100_lognn_wc02_fir15_dexp_fit05x0_40
950	74	0.282	0.386	0.464	fb18ch100_lognn_wc02_fir15_dexp_fit05x2
951	74	0.258	0.364	0.436	fb18ch100_lognn_wc02_fir15_dexp_fit05x2_40
952	74	0.295	0.391	0.471	fb18ch100_lognn_wc02_fir15_dexp_fit05x2_60
953	74	0.264	0.369	0.446	fb18ch100_lognn_wc02_fir15_dexp_fit05x3
954	74	0.257	0.363	0.434	fb18ch100_lognn_wc02_fir15_dexp_fit05x3_40
955	74	0.281	0.387	0.465	fb18ch100_lognn_wc02_fir15_dexp_fit05x4
956	74	0.257	0.363	0.436	fb18ch100_lognn_wc02_fir15_dexp_fit05x4_40
957	74	0.242	0.346	0.417	fb18ch100_lognn_wc02_fir15_dexp_fit05x_40
958	74	0.301	0.393	0.473	fb18ch100_lognn_wcg04_fir15_dexp_fit05vb
959	74	0.268	0.361	0.436	fb18ch100_normp_logn_wc02_fir15_dexp_fit09
960	74	0.270	0.363	0.441	fb18ch100_norm_logn_wc02_fir15_dexp_fit09
961	74	0.281	0.360	0.436	fb18ch100_norm_logn_wc02_fir15_norm_dexp_fit09
962	74	0.221	0.316	0.441	gt36ch200_logfree_wc01_fir30_siglog100_fit10
963	75	0.299	0.390	0.469	fb18ch100_lognn_wc02_fir15_siglog100_fit05h_fit05c
964	75	0.288	0.396	0.477	fb18ch100_lognn_wcg04_fir15_siglog100_fit05h
965	75	0.293	0.394	0.476	fb18ch100_lognn_wcg04_fir15_siglog100_fit05h_fit05c
966	75	0.285	0.388	0.468	fb18ch100_normp_logn_wc02_fir15_siglog100_fit05g_fit05c
967	75	0.278	0.370	0.446	fb18ch100_norm_logn_wc02_fir15_siglog100_fit05g_fit05c
968	75	0.278	0.366	0.442	fb18ch100_norm_logn_wc02_fir15_siglog100_fit09
969	75	0.226	0.328	0.404	fb18ch200_lognn_wc02_fir15_siglog100_fit05h_fit05c
970	75	0.161	0.251	0.304	fb18ch400_lognn_wc02_fir15_siglog100_fit05h_fit05c
971	79	0.294	0.395	0.476	fb18ch100_lognn_wcm04_fir15_siglog100_fit05h_fit05c
972	84	0.223	0.315	0.440	gt36ch200_logfree_wc01_fir40_siglog100_fit10
973	85	0.279	0.378	0.473	gt18ch100_logfree_wc02_fir20_siglog100_fit05
974	86	0.273	0.359	0.434	fb18ch100_norm_lognbz_wc02_fir20_dexp_fit09
975	87	0.271	0.371	0.446	fb18ch100_lognn_wcg05_fir15_fit05c
976	87	0.304	0.379	0.457	fb24ch100_lognn_wc02_fir15_siglog100_fit05h_fit05c
977	88	0.272	0.364	0.439	gt12ch100_logfree_wc03_fir15_siglog100_fit05
978	89	0.228	0.296	0.363	gt36ch200_logn_wc02_p1z1_siglog_fit10
979	92	0.298	0.394	0.474	fb18ch100_lognn_wcg05_fir15_siglog100_fit05h_fit05c
980	94	0.222	0.315	0.440	gt36ch200_logfree_wc01_fir50_siglog100_fit10
981	100	0.175	0.262	0.367	gt12ch200_wc03_fir20_fit10
982	101	0.202	0.293	0.409	gt12ch200_logn_wc03_fir20_fit10
983	104	0.274	0.372	0.448	fb18ch100_lognn_wcg06_fir15_fit05c
984	105	0.234	0.328	0.403	fb18ch200_lognn_wc02_fir30_siglog100_fit05h_fit05c
985	106	0.274	0.368	0.444	gt12ch100_logfree_wc03c_fir20_siglog100_fit05c
986	106	0.249	0.327	0.409	gt12ch100_logfree_wc03_fir20_siglog100_crossvalidatedfit05
987	106	0.249	0.329	0.411	gt12ch100_logfree_wc03_fir20_siglog100_crossvalidatedfit05b
988	106	0.262	0.360	0.451	gt12ch100_logfree_wc03_fir20_siglog100_fit05a

989	106	0.264	0.366	0.458	gt12ch100_logfree_wc03_fir20_siglog100_fit05b
990	106	0.274	0.367	0.460	gt12ch100_logfree_wc03_fir20_siglog100_fit05c
991	106	0.251	0.328	0.410	gt12ch100_xvalnmse1_logfree_wc03_fir20_siglog100_fit05
992	106	0.254	0.310	0.385	gt12ch100_xvalnmse1_logfree_wc03_fir20_siglog100_fit10
993	106	0.249	0.329	0.411	gt12ch100_xvalnmse2_logfree_wc03_fir20_siglog100_fit05
994	106	0.251	0.318	0.397	gt12ch100_xvalnmse2_logfree_wc03_fir20_siglog100_fit10
995	106	0.219	0.312	0.436	gt12ch200_logn_wc03_fir20_siglog_fit10
996	106	0.286	0.381	0.460	gt18ch100_logfree_wc03_fir15_siglog100_fit05
997	108	0.315	0.372	0.449	fb18ch100_lognn_wc03_fir15_dexp_fit05vb
998	108	0.315	0.374	0.451	fb18ch100_lognn_wc03_fir15_dexp_fit05vc
999	109	0.308	0.384	0.463	fb18ch100_lognn_wc03_fir15_siglog100_fit05h_fit05c
1000	111	0.313	0.377	0.454	fb36ch100_lognn_wc02_fir15_siglog100_fit05h_fit05c
1001	121	0.231	0.316	0.442	gt36ch200_logfree_wc02_fir20_siglog100_fit10
1002	121	0.233	0.321	0.396	gt36ch200_logn_wc02_fir20_siglog_fit10
1003	124	0.287	0.382	0.479	gt18ch100_logfree_wc03_fir20_siglog100_fit05
1004	130	0.237	0.286	0.352	gt36ch200_logn_wc03_p1z1_siglog_fit10
1005	142	0.323	0.371	0.447	fb18ch100_lognn_wc04_fir15_dexp_fit05vb
1006	142	0.323	0.370	0.445	fb18ch100_lognn_wc04_fir15_dexp_fit05vc
1007	143	0.312	0.383	0.462	fb18ch100_lognn_wc04_fir15_siglog100_fit05h_fit05c
1008	145	0.174	0.268	0.322	fb18ch400_lognn_wc02_fir50_siglog100_fit05h_fit05c
1009	163	0.290	0.380	0.476	gt18ch100_logfree_wc04_fir20_siglog100_fit05
1010	176	0.328	0.365	0.440	fb18ch100_lognn_wc05_fir15_dexp_fit05vc
1011	178	0.236	0.312	0.432	gt36ch200_logfree_wc03_fir20_siglog100_fit10
1012	178	0.238	0.316	0.392	gt36ch200_logn_wc03_fir20_siglog_fit10
1013	181	0.254	0.340	0.410	gt12ch100_log2_fir15_fit05
1014	186	0.281	0.356	0.428	gt12ch100_log2_fir15_siglog100_fit05
1015	187	0.288	0.362	0.436	gt12ch100_logfree_fir15_siglog100_fit05
1016	241	0.257	0.341	0.427	gt12ch100_log2_fir20_fit05
1017	241	0.170	0.252	0.353	gt12ch200_fir20_fit10
1018	246	0.288	0.352	0.440	gt12ch100_log2_fir20_siglog100_fit05
1019	247	0.318	0.344	0.415	gt12ch100_logfree_fir20_siglog100_fit05c
1020	247	0.314	0.351	0.423	gt12ch100_logfree_fir20_siglog100_fit10
1021	271	0.267	0.356	0.428	gt18ch100_log2_fir15_fit05
1022	272	0.296	0.366	0.440	fb18ch100_lognn_fir15_fit05c
1023	276	0.354	0.334	0.402	fb18ch100_lognn_fir15_dexp_fit05vb
1024	276	0.276	0.374	0.450	fb18ch100_lognn_fir15_dexp_fit05w30
1025	276	0.281	0.380	0.457	fb18ch100_lognn_fir15_dexp_fit05w35
1026	276	0.299	0.389	0.468	fb18ch100_lognn_fir15_dexp_fit05w40
1027	276	0.314	0.382	0.461	fb18ch100_lognn_fir15_dexp_fit05w45
1028	276	0.328	0.372	0.448	fb18ch100_lognn_fir15_dexp_fit05w50
1029	276	0.343	0.356	0.429	fb18ch100_lognn_fir15_dexp_fit05w55
1030	276	0.354	0.336	0.406	fb18ch100_lognn_fir15_dexp_fit05w60
1031	276	0.362	0.317	0.382	fb18ch100_lognn_fir15_dexp_fit05w65
1032	276	0.304	0.385	0.463	fb18ch100_lognn_fir15_dexp_fit05x
1033	276	0.367	0.310	0.374	fb18ch100_lognn_fir15_dexp_fit05x0
1034	276	0.346	0.347	0.419	fb18ch100_lognn_fir15_dexp_fit05x0_40
1035	276	0.307	0.382	0.459	fb18ch100_lognn_fir15_dexp_fit05x2
1036	276	0.272	0.376	0.452	fb18ch100_lognn_fir15_dexp_fit05x2_40
1037	276	0.335	0.366	0.440	fb18ch100_lognn_fir15_dexp_fit05x2_60
1038	276	0.302	0.386	0.464	fb18ch100_lognn_fir15_dexp_fit05x3
1039	276	0.271	0.373	0.448	fb18ch100_lognn_fir15_dexp_fit05x3_40
1040	276	0.305	0.383	0.460	fb18ch100_lognn_fir15_dexp_fit05x4
1041	276	0.270	0.373	0.447	fb18ch100_lognn_fir15_dexp_fit05x4_40
1042	276	0.269	0.373	0.448	fb18ch100_lognn_fir15_dexp_fit05x_40
1043	276	0.300	0.369	0.444	gt18ch100_log2_fir15_siglog100_fit05

1044	277	0.338	0.365	0.440	fb18ch100_lognn_fir15_siglog100_fit05h_fit05c
1045	277	0.252	0.312	0.385	fb18ch200_lognn_fir15_siglog100_fit05h_fit05c
1046	277	0.175	0.242	0.293	fb18ch400_lognn_fir15_siglog100_fit05h_fit05c
1047	277	0.310	0.377	0.454	gt18ch100_logfree_fir15_siglog100_fit05
1048	277	0.284	0.362	0.435	gt18ch100_logn_fir15_siglog100b_fit05c
1049	277	0.294	0.367	0.442	gt18ch100_logn_fir15_siglog100b_fit05g_fit05c
1050	277	0.336	0.346	0.416	gt18ch100_logn_fir15_siglog100b_fit05h_fit05c
1051	277	0.320	0.371	0.448	gt18ch100_logn_fir15_siglog100b_fit09
1052	277	0.346	0.344	0.414	gt18ch100_logn_fir15_siglog100b_fit09c
1053	277	0.356	0.335	0.403	gt18ch100_logn_fir15_siglog100b_fitgen06
1054	278	0.338	0.364	0.439	fb18ch100_lognbn_fir15_siglog100_fit05h_fit05c
1055	361	0.270	0.354	0.426	gt18ch100_log2_fir20_fit05
1056	366	0.307	0.364	0.437	gt18ch100_log2_fir20_siglog100_fit05
1057	367	0.354	0.359	0.432	fb24ch100_lognn_fir15_siglog100_fit05h_fit05c
1058	367	0.318	0.371	0.463	gt18ch100_logfree_fir20_siglog100_fit05
1059	547	0.277	0.300	0.371	fb18ch200_lognn_fir30_siglog100_fit05h_fit05c
1060	547	0.378	0.342	0.412	fb36ch100_lognn_fir15_siglog100_fit05h_fit05c
1061	907	0.211	0.241	0.291	fb18ch400_lognn_fir50_siglog100_fit05h_fit05c
